# Spatial and temporal epidemiology of malaria in extra-Amazonian regions of Brazil

**DOI:** 10.1186/s12936-015-0934-6

**Published:** 2015-10-15

**Authors:** Camila Lorenz, Flávia Virginio, Breno S. Aguiar, Lincoln Suesdek, Francisco Chiaravalloti-Neto

**Affiliations:** Instituto Butantan, Avenida Vital Brasil, 1500, São Paulo, CEP 05509-300 Brazil; Biologia da Relação Patógeno-Hospedeiro-Instituto de Ciências Biomédicas-USP, São Paulo, Brazil; Instituto de Medicina Tropical, Avenida Dr Enéas Carvalho de Aguiar, 470, São Paulo, CEP 05403-000 Brazil; Departamento de Epidemiologia, Faculdade de Saúde Pública, Universidade de São Paulo, Av Dr Arnaldo, 715, São Paulo, CEP 05509-300 Brazil

**Keywords:** Malaria, Imported, Autochthonous, Introduced, Epidemiology, Bromeliad-malaria, Outbreaks, *Plasmodium falciparum*, *Plasmodium vivax*

## Abstract

**Background:**

Mosquitoes, *Plasmodium* parasites, and humans live in sympatry in some extra-Amazonian regions of Brazil. Recent migrations of people from Amazonia and other countries to extra-Amazonian regions have led to many malaria outbreaks. Lack of relevant expertise among health professionals in non-endemic areas can lead to a neglect of the disease, which can be dangerous given its high fatality rate. Therefore, understanding the spatial and temporal epidemiology of malaria is essential for developing strategies for disease control and elimination. This study aimed to characterize imported (IMP) and autochthonous/introduced (AU/IN) cases in the extra-Amazonian regions and identify risk areas and groups.

**Methods:**

Epidemiological data collected between 2007 and 2014 were obtained from the Notifiable Diseases Information System of the Ministry of Health (SINAN) and from the Department of the Unified Health System (DATASUS). High malaria risk areas were determined using the Local Indicator of Spatial Association. IMP and AU/IN malaria incidence rates were corrected by Local Empirical Bayesian rates.

**Results:**

A total of 6092 malaria cases (IMP: 5416, 88.9 %; AU/IN: 676, 11.1 %) was recorded in the extra-Amazonian regions in 2007–2014. The highest numbers of IMP and AU/IN cases were registered in 2007 (n = 862) and 2010 (n = 149), respectively. IMP cases were more frequent than AU/IN cases in all states except for Espírito Santo. Piauí, Espírito Santo, and Paraná states had high incidences of AU/IN malaria. The majority of infections were by *Plasmodium falciparum* in northeast and southeast regions, while *Plasmodium vivax* was the predominant species in the south and mid-west showed cases of dual infection. AU/IN malaria cases were concentrated in the coastal region of Brazil, which contains the Atlantic Forest and hosts the *Anopheles* transmitters. Several malaria clusters were also associated with the Brazilian Pantanal biome and regions bordering the Amazonian biome.

**Conclusion:**

Malaria is widespread outside the Amazonian region of Brazil, including in more urbanized and industrialized states. This fact is concerning because these highly populated areas retain favourable conditions for spreading of the parasites and vectors. Control measures for both IMP and AU/IN malaria are essential in these high-risk areas.

**Electronic supplementary material:**

The online version of this article (doi:10.1186/s12936-015-0934-6) contains supplementary material, which is available to authorized users.

## Background

Malaria remains a major public health problem in Brazil, with approximately 145,000 cases reported in 2014 [1]. Most of the malaria cases occur in the Brazilian Amazonia region (the Amazon Biome), an endemic area of malaria in which the mosquito *Anopheles darlingi* is incriminated as the main vector [2]. However, recent migrations of people from the Amazonian region and/or other countries to the extra-Amazonian regions led to outbreaks of secondary imported cases (i.e. introduced malaria) [3–6]. It is important to note that mosquitoes, *Plasmodium*, and humans also live in sympatry in some extra-Amazonian regions. An example of this is the dynamic “bromeliad malaria” [7], in which autochthonous cases are associated with the Atlantic Forest biome where *Kerteszia* subgenus is considered the primary vector of malaria [8].

In Brazil, mainly three *Plasmodium* species are associated with native human malaria cases: *Plasmodium vivax*, *Plasmodium falciparum,* and *Plasmodium malariae* [1]. The relative incidence of each *P. vivax* and *P. falciparum* malaria was approximately 50 % in 1988 [9]. A shift occurred after 1990, when 44.3 % of cases were due to *P. falciparum*, and by 2014 *P. vivax* became the predominant species in the Amazonian region, with only 16.18 % of cases being due to *P. falciparum*. Although *P. vivax* causes a less dangerous type of malaria associated with low mortality, the morbidity in endemic communities is very high, which makes it similar in this regard to *P. falciparum* [9].

Only 19 % of all malaria cases in the extra-Amazonian regions are diagnosed and treated within 48 h after symptoms onset, in contrast to 60 % of malaria cases in the Amazonian region. This may explain the high proportion of severe malaria cases in non-endemic areas. Accordingly, the malaria fatality rate in the extra-Amazonian regions is higher than in the Amazonian region [6]. Furthermore, the lack of expertise among health professionals of non-endemic areas in diagnosis and management of malaria [6] and poor general public knowledge serve as aggravating factors in disease treatment and control [10, 11]. For instance, malaria is frequently misdiagnosed as dengue in the city of Rio de Janeiro [12].

Understanding the epidemiology of malaria in the temporal and spatial dimensions is essential for planning control strategies and disease elimination. Therefore, the aims of this study were to: (a) characterize cases of autochthonous/introduced (AU/IN) and imported (IMP) malaria in the extra-Amazonian regions in 2007–2014; (b) assess the dynamics of the etiological agents of malaria in Brazil (Amazonian and extra-Amazonian regions) and from other countries; and (c) detect risk areas and groups.

## Methods

**Fig. 1:**
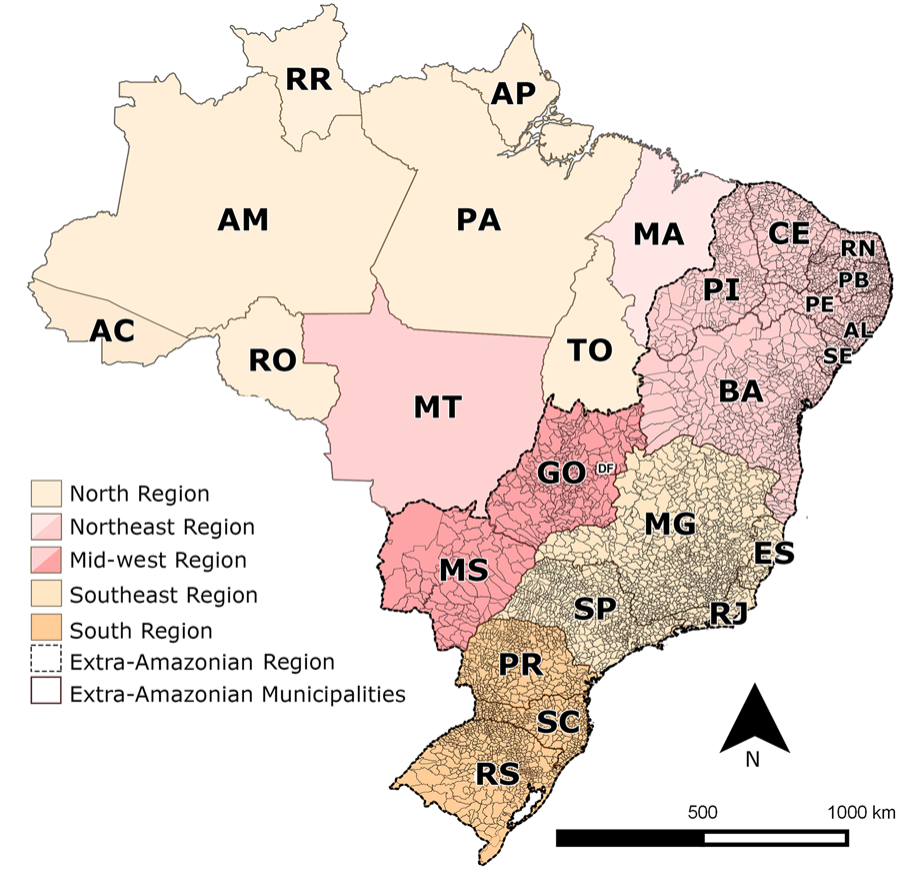
Map of Brazil showing the States belonging to the extra-Amazonian region. Modified from IBGE [13]

This was a descriptive study to evaluate the occurrence of AU/IN and IMP malaria in the following Brazilian states of the extra-Amazonian regions (Fig. 1): south: Rio Grande do Sul (RS), Santa Catarina (SC), and Paraná (PR); southeast: São Paulo (SP), Rio de Janeiro (RJ), Espírito Santo (ES), and Minas Gerais (MG); northeast: Piauí (PI), Ceará (CE), Rio Grande do Norte (RN), Paraiba (PB), Pernambuco (PE), Alagoas (AL), Sergipe (SE), and Bahia (BA); and mid-west: Goiás(GO), Mato Grosso do Sul (MS), and Distrito Federal (DF). These states together comprise 4762 municipalities [13].

According to the epidemiological malaria classification of the World Health Organization [14], the cases of this study were classified into (1) IMP: cases of malaria contracted outside the area where the diagnosis was made; (2) AU: cases of malaria contracted in the city or county where the diagnosis was made; and (3) IN: direct secondary cases if they constituted the first local transmission link after a known IMP case. AU and IN cases were combined because available data do not permit one to distinguish between them; IMP cases from the Amazonian region and from other countries were also pooled for the same reason.

Reports of malaria cases were obtained from the Notifiable Diseases Information System (SINAN), which is publicly available from the Department of the Unified Health System (DATASUS), Brazilian Ministry of Health (see Additional file 1). The following information about malaria cases that occurred between 2007 and 2014 was collected: case classification (AU/IN or IMP), parasitological analysis result (*P. vivax*, *P. falciparum*, or both), sex, age group, year of occurrence, residence municipality, country of infection, and occurrence of death. Parasitological analysis results positive for *P. falciparum* or [*P. falciparum* + *P. falciparum* gametocyte] were considered to represent *P. falciparum* infection. Furthermore, numbers of inhabitants according to state and year were obtained from the Brazilian Institute of Geography and Statistics [13].

The information on municipality and year was incorporated into the georeferenced map of the municipalities belonging to the extra-Amazonian regions, which was made available by the IBGE in latitude-longitude projection and Datum SIRGAS 2000. Incidence rates were calculated according to the year and case classification in the entire extra-Amazonian region and in the individual states during the study period. The proportional distribution of malaria cases by *Plasmodium* type, geographical region, and malaria mortality rates for all the extra-Amazonian regions according to year for the entire study period was obtained in DATASUS [15].

The incidence rates of AU/IN and IMP malaria and mortality rates were calculated throughout the study period for all the municipalities of the extra-Amazonian regions. Spatial aggregation patterns of these rates were assessed using the Local Moran Index or Local Indicator of Spatial Association (LISA), which allowed identifying significant spatial clusters (α < 0.05). Among the 4 possible groups identified by LISA, the group was chosen that is used to classify locations according to the “high–high” category (i.e. spatial units with high malaria rates surrounded by units with high malaria rates, which can be characterized as high-risk cluster areas) [16].

AU/IN and IMP malaria incidence rates were obtained for the following three stages according to municipalities: beginning (2007), middle (2010), and ending (2014). To correct for distortions in the incidence rates caused by random fluctuations resulting from small populations of some municipalities, the Local Empirical Bayesian rate was used to re-estimate the settlement rate based on neighbours and municipality population [17, 18]. The neighbourhood criterion was contiguity (i.e. locations that share a side or point were defined as neighbours).

The graphs were created using the software Statistica 7.0 [19] and Microsoft Excel (Microsoft, 1987). To construct maps and detect spatial clusters, we used QGIS 2.8.3.Wien, TerraView 4.1.0, GeoDa 1.6.7.9, and Adobe Photoshop 1.6 (Adobe Systems, San Jose 165, CA, USA).

## Results

### Temporal epidemiology

**Fig. 2:**
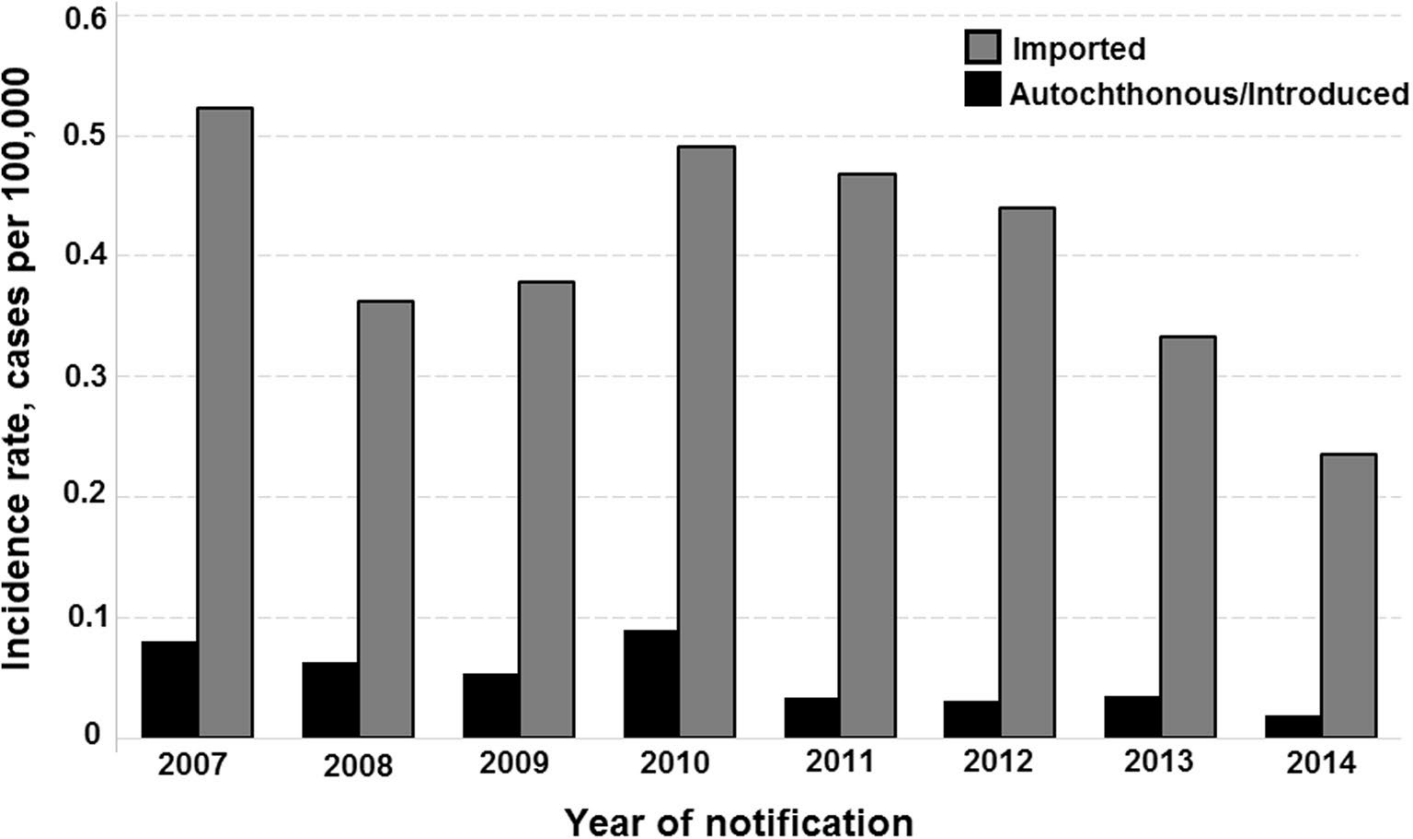
Incidence rates of autochthonous/introduced and imported malaria from 2007 to 2014 in extra-Amazonian region of Brazil

Between 2007 and 2014, 6092 malaria cases (IMP: 5416, 88.9 %; AU/IN: 676, 11.1 %) were recorded in the extra-Amazonian regions. All cases were confirmed by thick blood smears. The years with the highest number of IMP and AU/IN cases were 2007 (862 cases, incidence rate of 0.52 cases per 100,000 inhabitants-year) and 2010 (149 cases, incidence rate of 0.09 cases per 100,000 inhabitants-year), respectively (Fig. 2). In 2014, both IMP and AU/IN malaria had the lowest incidence rates.

**Fig. 3:**
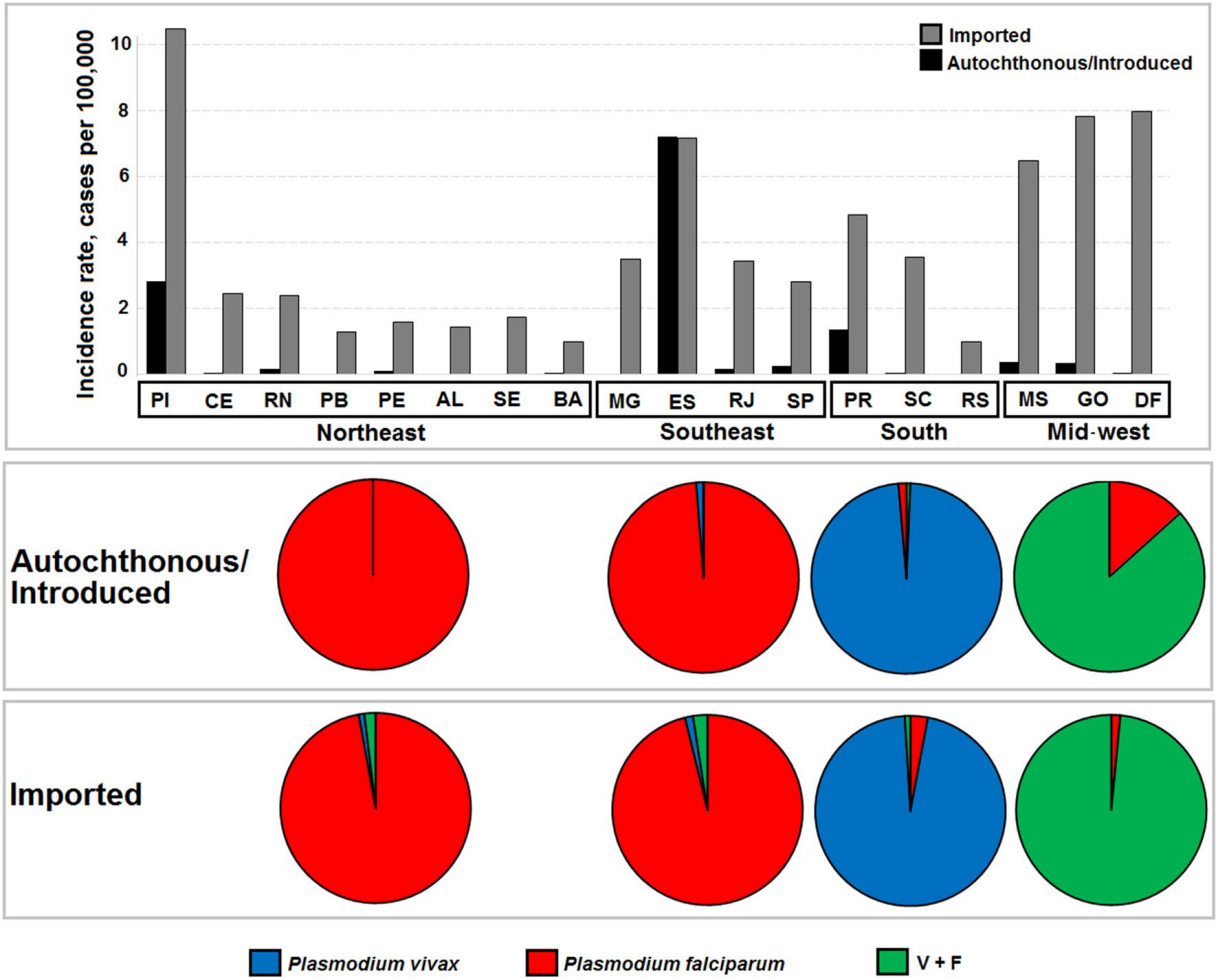
Incidence rates of autochthonous/introduced and imported malaria cases (per 100,000 inhabitants/year) by state from 2007 to 2014 in extra-Amazonian region of Brazil and proportional presence of *Plasmodium* in each region. The incidence rate was calculated based on follow raw data: autochthonous/introduced cases by municipality of infection; imported cases by residence municipality. Positive parasitological results to *P. vivax* (*blue*), *P. falciparum* (*red*) and double-infection (*green*)

IMP cases were more frequent than AU/IN cases in all states except ES (Fig. 3). The mid-west region had the highest IMP malaria incidence rates between 2007 and 2014. PI also had more IMP cases than other northeast states. The incidence rates of AU/IN malaria were higher than the average total rate in the extra-Amazonian regions (0.05 cases/100,000 inhabitants-year) in PR, ES, and PI. According to parasitological examinations, the patterns of *P. vivax* and *P. falciparum* infections were similar among the reported cases in the northeast and southeast regions. A large number of *P. falciparum* infections were observed as IMP and AU/IN cases in these regions. *Plasmodium vivax* infection was predominant in the south, representing 98 % of the total AU cases and 97 % of the IMP cases. A high number of double-infection cases (i.e. with two species of *Plasmodium* present in patient’s blood) were observed in the mid-west region.

**Fig. 4:**
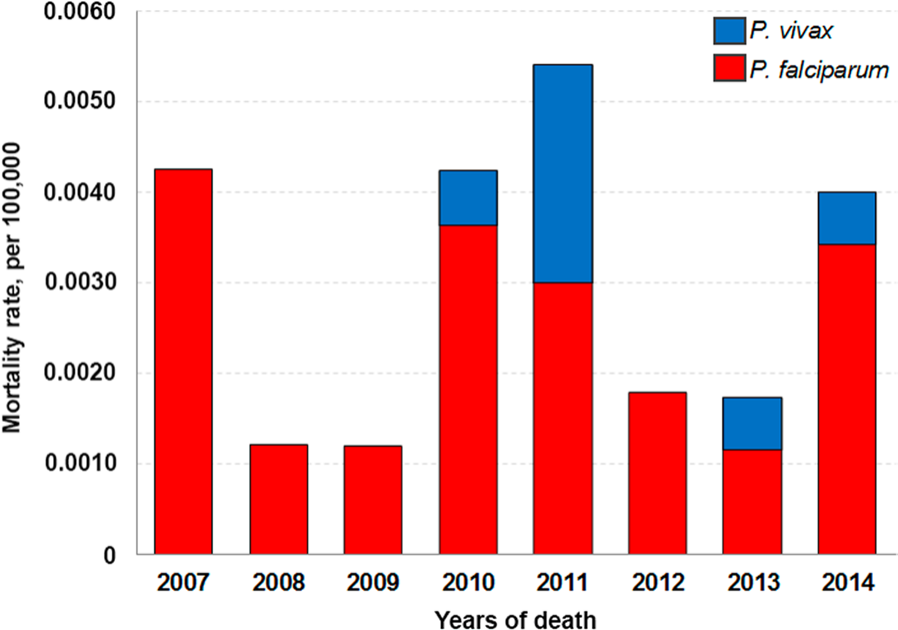
Mortality rate by species of *Plasmodium* from 2007 to 2014 in extra-Amazonian region of Brazil. Data of 2007–2013 were obtained from Mortality Information System (SIM/DATASUS); data of 2014 were obtained from SVS-MS Epidemiological Bulletin [1]

During the eight-year study period, there were 40 deaths from malaria in the extra-Amazonian regions. The overall mortality rate varied widely between the years (Fig. 4) and did not follow the distribution of malaria cases, peaking in 2011 (9 deaths, 0.0054 deaths per 100,000 inhabitants/year). Men accounted for 80 % of IMP and 75 % of AU/IN malaria cases. The highest incidence for both types of malaria infections was in the 20–30 years age group.

**Fig. 5:**
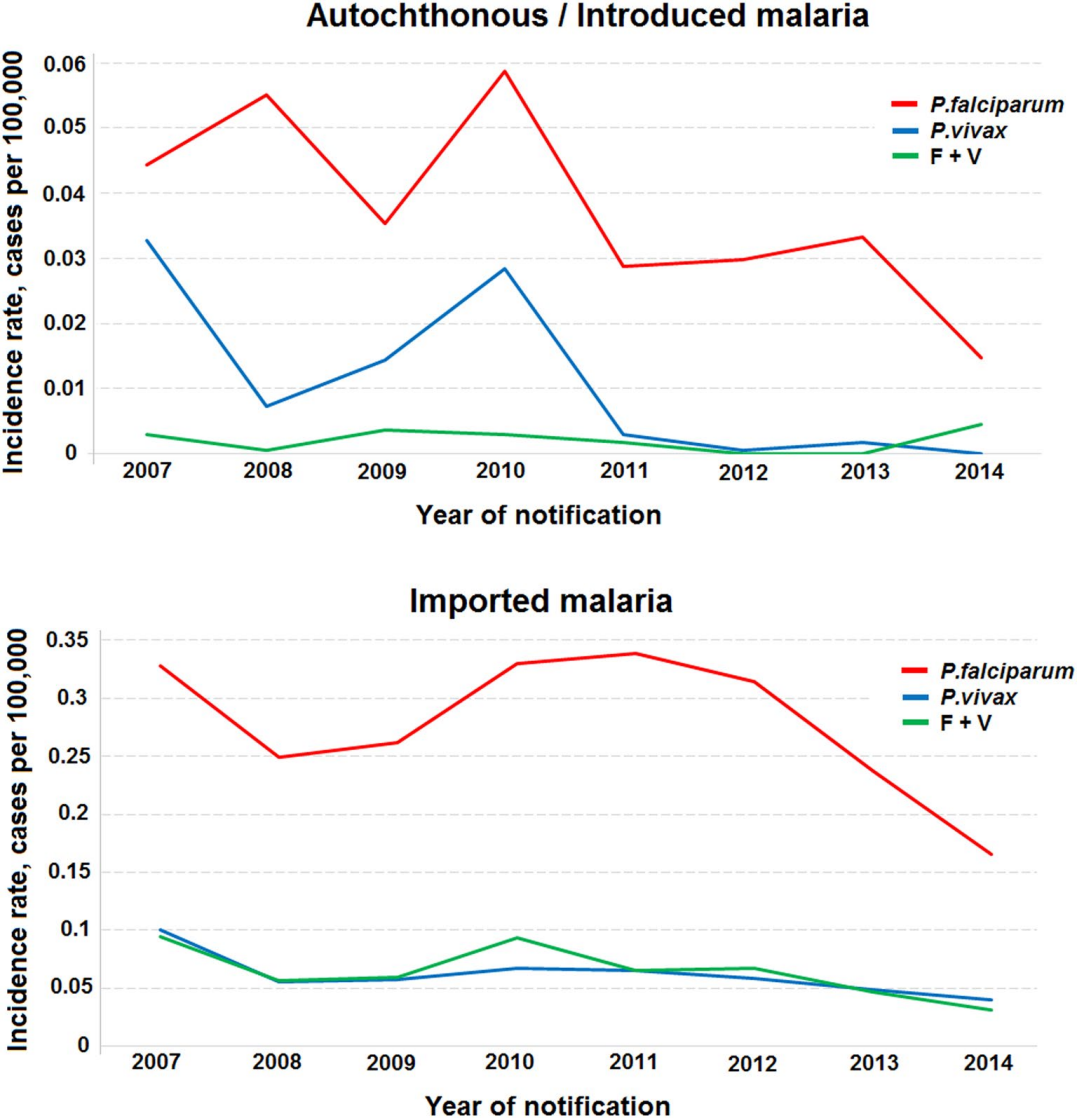
Incidence rates of autochthonous/introduced and imported malaria cases by parasitological result from 2007 to 2014 in extra-Amazonian region of Brazil. *P. vivax* (*blue*), *P. falciparum* (*red*) and double-infection (*green*)

**Fig. 6:**
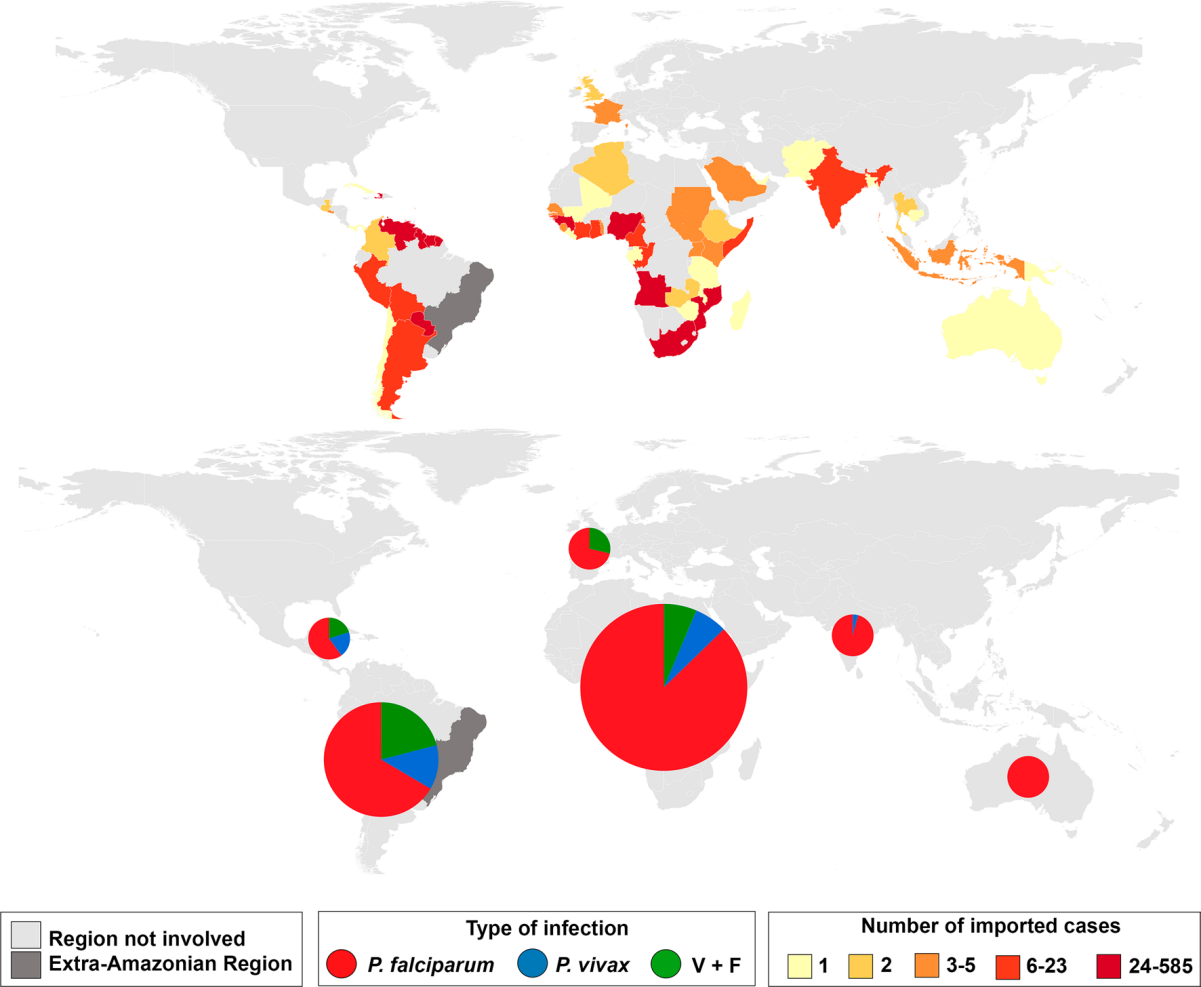
Imported malaria cases from other countries to extra-Amazonian region in the period of 2007–2014. North America: 51 cases; South America: 520 cases; Africa: 1,100 cases; Europe: 7 cases; Asia: 24 cases; Oceania: 1 case. *P. vivax* (*blue*), *P. falciparum* (*red*) and double-infection (*green*)

Over the last few years, *P. falciparum* has been responsible for the majority of IMP and AU/IN malaria cases in the extra-Amazonian regions (Fig. 5). In addition, a significant decrease in incidence rate in recent years is evident. IMP cases originated from other countries to extra-Amazonian region are shown in Fig. 6. Africa was the main source of foreign IMP cases in the eight-year study period (1110), followed by South America (520). Most IMP cases were caused by *P. falciparum*.

### Spatial epidemiology

**Fig. 7:**
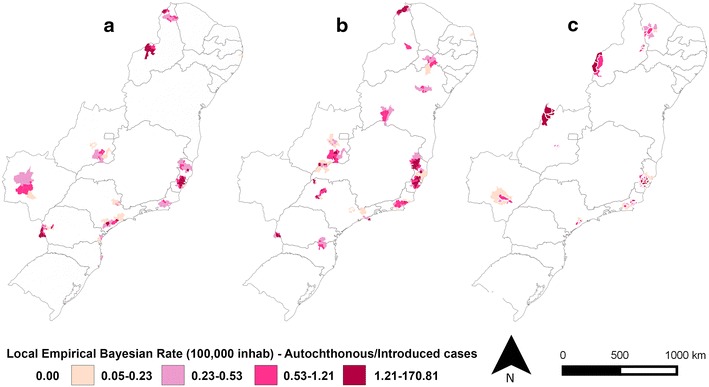
Bayesian incidence rates of autochthonous/introduced (AU/IN) malaria in **a** 2007, **b** 2010 (year with most cases) and **c** 2014 in extra-Amazonian region of Brazil. In this analysis the species of *Plasmodium* were grouped

**Fig. 8:**
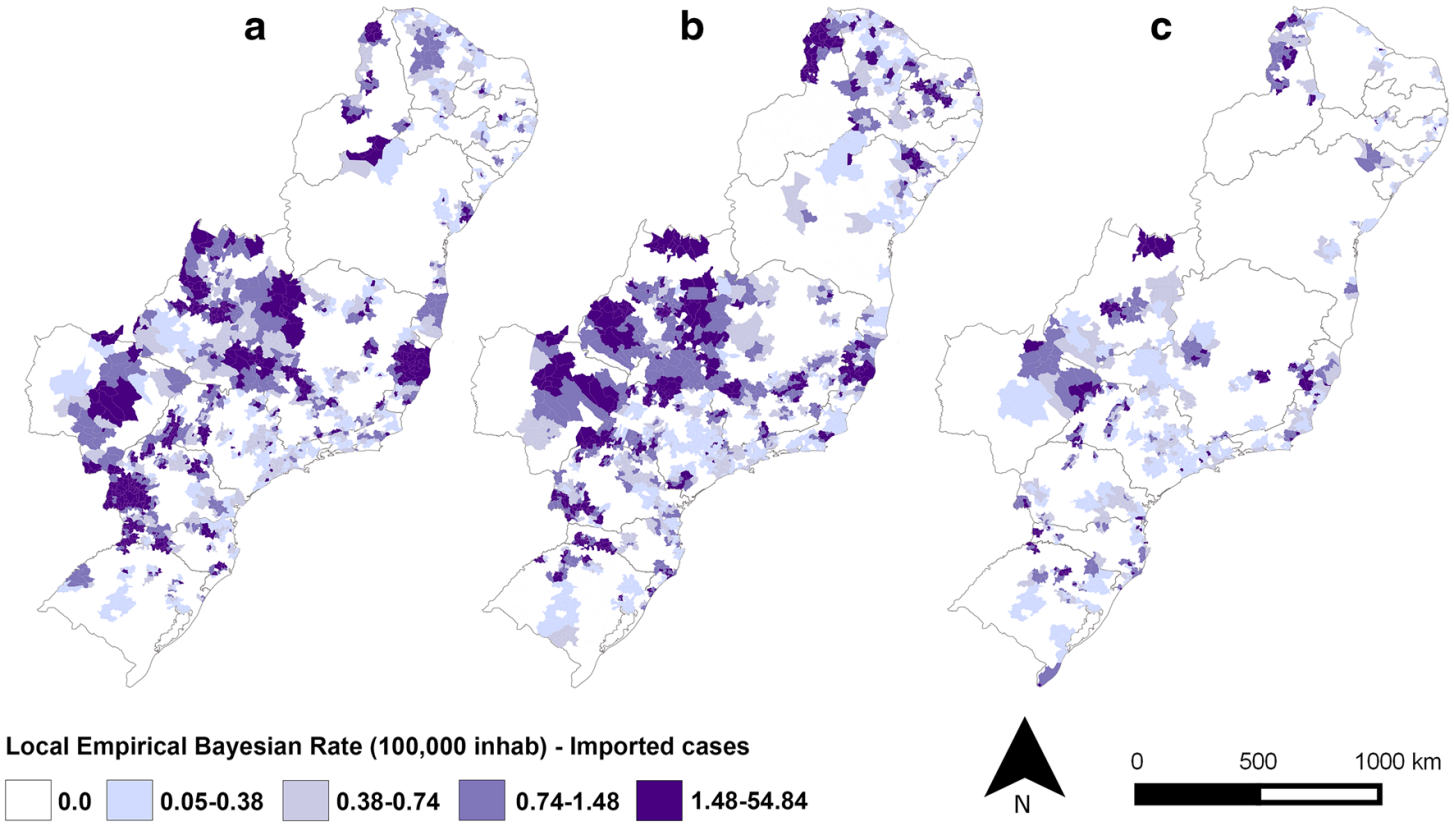
Bayesian incidence rates of imported (IMP) malaria in **a** 2007, **b** 2010 and **c** 2014 in extra-Amazonian region of Brazil. In this analysis the species of *Plasmodium* were grouped

The IMP and AU/IN malaria incidence rates in the extra-Amazonian regions varied within the 8-year study period (Figs. 7, 8), with the lowest values for both observed in 2014. The incidence rates of AU/IN cases were lower than those of IMP cases in the majority of municipalities analysed. The highest occurrence of AU/IN cases was in 2010, with >100 cases per 100,000 inhabitants-year after Bayesian correction in one municipality (São Miguel do Iguaçu in PR).

**Fig. 9:**
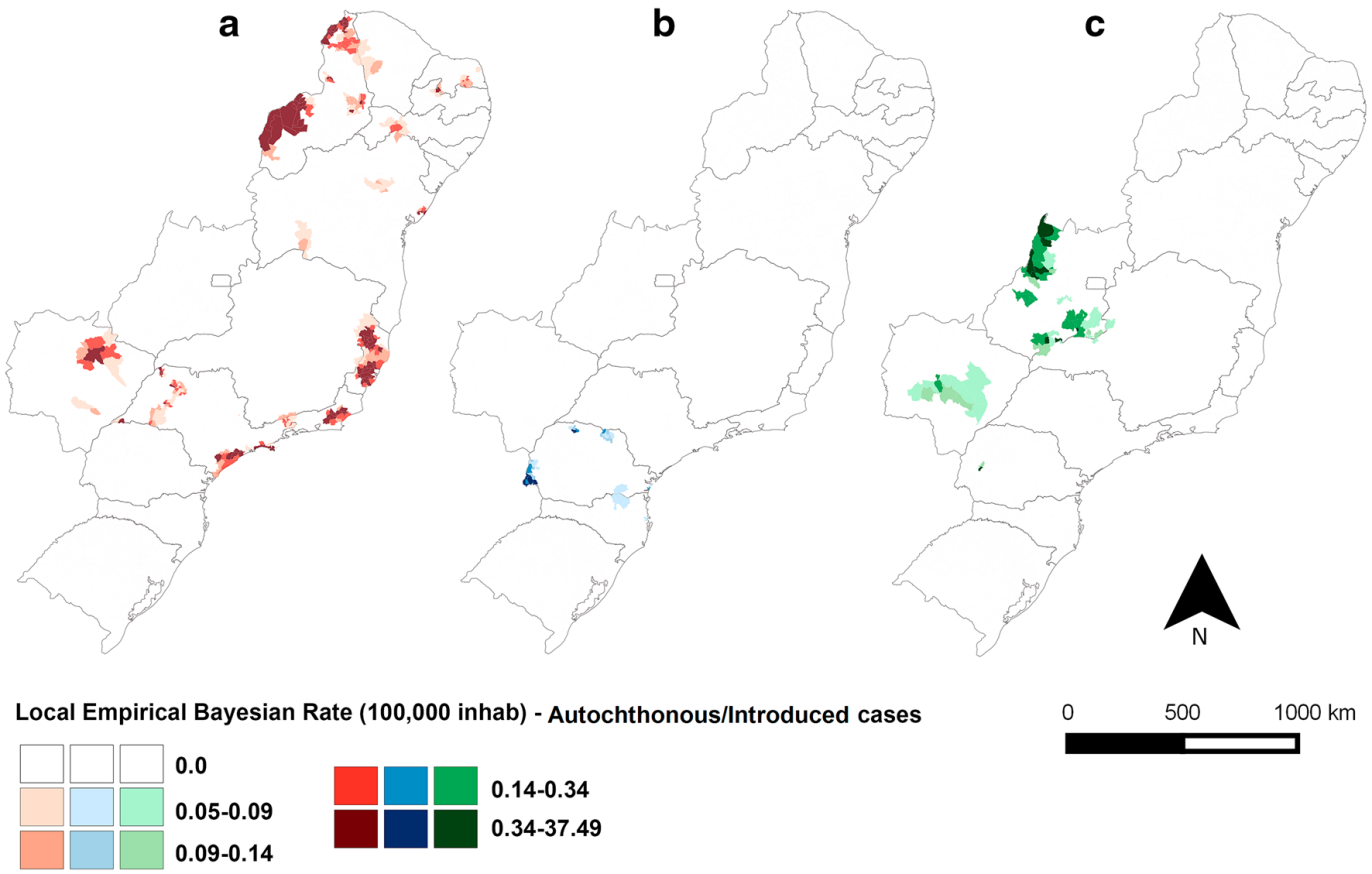
Bayesian incidence rates of autochthonous/introduced (AU/IN) malaria separate by species of *Plasmodium*: **a** *P. falciparum*, **b** *P. vivax* and **c** double-infection, in extra-Amazonian region of Brazil in the period of 2007–2014

**Fig. 10:**
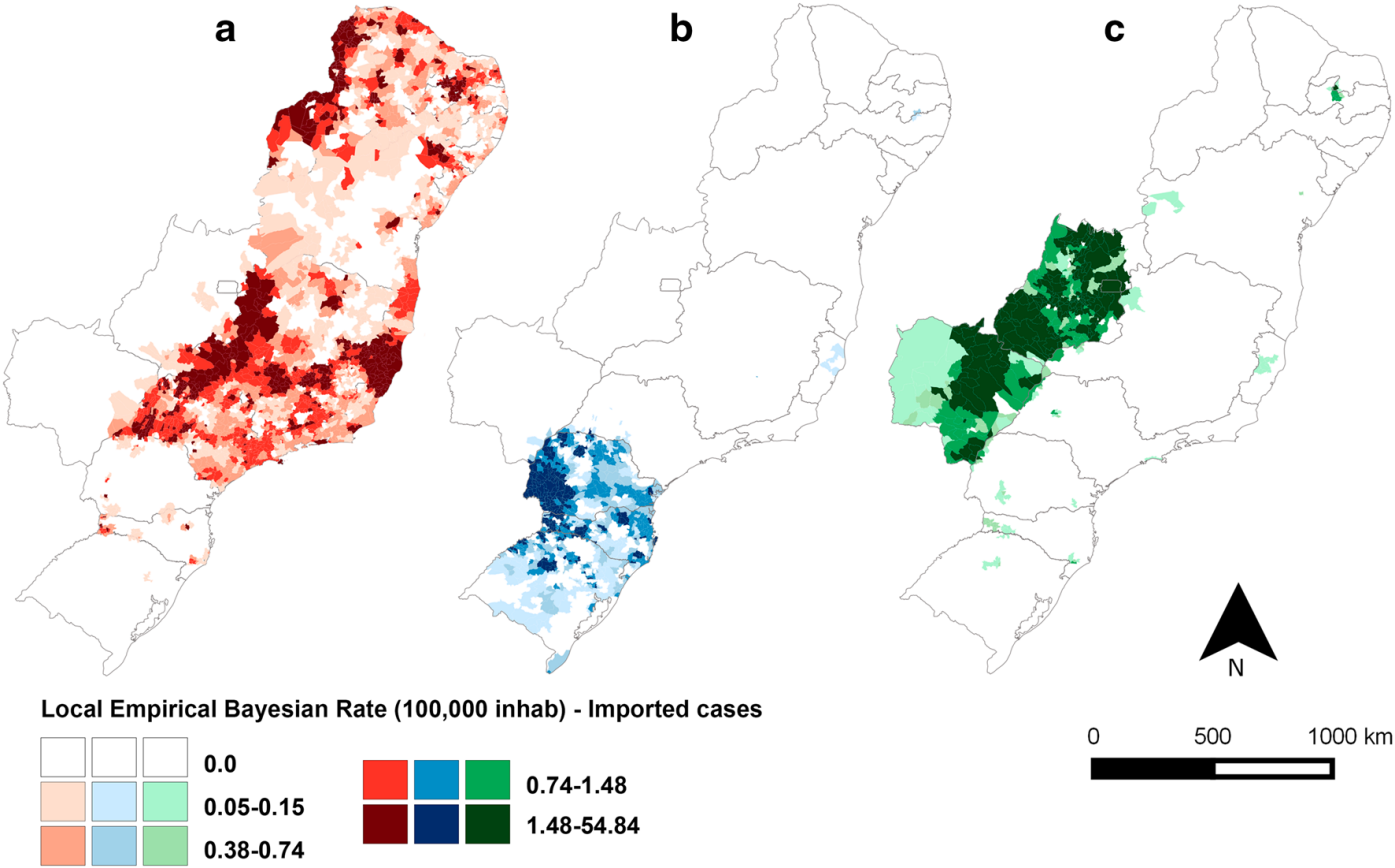
Bayesian incidence rates of imported (IMP) malaria separate by species of *Plasmodium*: **a** *P. falciparum*, **b** *P. vivax* and **c** double-infection, in extra-Amazonian region of Brazil in the period of 2007–2014

When the cases were grouped according to the species of *Plasmodium* (Figs. 9, 10), a clear separation was observed between the states for both IMP and AU/IN infections: *P. vivax* malaria was concentrated in the south (mainly in PR), double-infection malaria occurred predominantly in the mid-west, and *P. falciparum* cases were registered throughout the rest of the country. Similar spatial patterns were found in the IMP and AU/IN maps because the types of *Plasmodium* infection were the same, suggesting that IMP cases may have initiated the AU/IN cases (outbreaks).

**Fig. 11:**
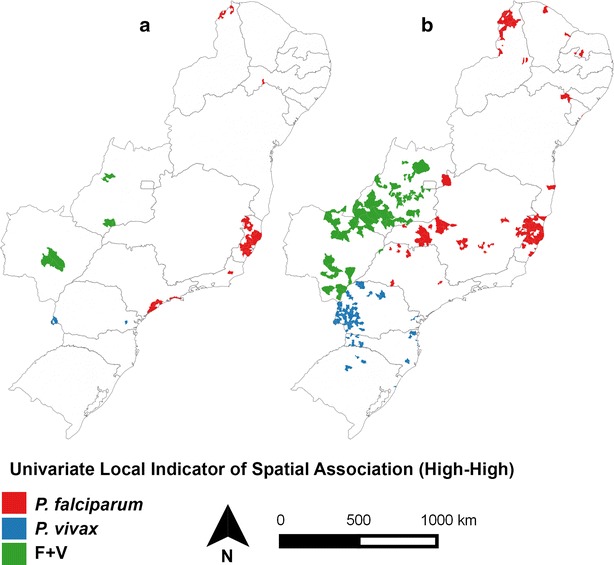
Map of municipalities in extra-Amazonian regions of Brazil classified, according to LISA, with high risk for occurrence of **a** autochthonous/introduced (AU/IN) malaria and **b** imported (IMP) malaria according species of *Plasmodium*. *P. vivax* (*blue*), *P. falciparum* (*red*) and double-infection (*green*)

High-risk clusters distributed in the extra-Amazonian regions were also grouped according to the types of *Plasmodium* infection during 2007–2014 (Fig. 11). An important high-risk area for AU/IN malaria was situated in SP and ES, which represent the Atlantic Forest biome. IMP cases were spread throughout all the Brazilian regions.

## Discussion

### Autochthonous/introduced and imported cases in extra-Amazonian regions

The number of IMP and AU/IN malaria cases in the Brazilian extra-Amazonian regions has declined in recent years. This coincides with the reduction in the number of malaria cases in the Amazonian region observed between 2007 and [1, 6] and with the 37 % drop in the worldwide incidence of malaria since 2000 [20]. This general trend reflects the 20-fold increase in global investments into eradication of the disease in the last 15 years [20]. Specifically in Brazil, the following preventive measures adopted by the population have been the main factor responsible for the decrease in the number of cases: use of personal protective equipment against insect bites, especially in risk areas; use of insecticide-treated mosquito nets; use of nets on doors and windows; use of repellent; and avoiding bathing sites during periods of higher activity of mosquitoes [21]. Although 99 % of malaria cases recorded in Brazil occurred in the Legal Amazon [22], the extra-Amazonian areas deserve special attention because of their large populations (about 87 % of the Brazilian population as reported by the IBGE in 2015 [13]). Furthermore, regions with mosquitoes, *Plasmodium,* and humans living in sympatry would facilitate AU/IN outbreaks.

The higher number of IMP malaria cases than AU/IN cases registered in the extra-Amazonian regions, with the exception of ES, could be a consequence of the increased migration of workers to endemic regions due to recent economic development and construction of hydroelectric plants [6]. In addition, the level of international migration to Brazil has been high in the last years. Thus, approximately 455,000 people migrated from foreign countries during 2000–2010. This resulted in significant increases in numbers of international migrants in GO, ES, and MG [13]. There was also a recent increase in the number of malaria cases imported from Africa in SP and RJ [23]. These patterns of migration to different regions of the country, lack of clinical management skills among health professionals, and limited number of locations for malaria diagnosis have created a serious public health concern in the non-endemic areas.

For cases of AU/IN malaria, there are two different contexts of transmission that were grouped in this study: outbreaks from IMP cases (introduced malaria) and bromeliad malaria [7]. Outbreaks usually occur when there is a combination of three factors: migration from the Amazonian region, presence of competent vector (usually *Anopheles darlingi*), and a susceptible population group. In such environment, one IMP case can lead to several IN cases. On the other hand, the frequency of bromeliads malaria remains virtually constant in the Atlantic Forest, with *An. cruzii* serving as the primary vector [24–27]. As an example, a high incidence rate of AU malaria observed in Espírito Santo can be explained by the topography and climatic characteristics that favour mosquito breeding. *An. darlingi* and *Anopheles aquasalis* have been incriminated as vectors of IN malaria in Espírito Santo, with the former present within the state and the latter restricted to maritime regions [28].

### Spatial epidemiology and risk groups

It was possible to outline the social profile of the subpopulation predominantly infected with malaria in the extra-Amazonian regions. Thus, the highest percentage of cases occurred in men of economically active age (20–39 years), suggesting that employment-driven displacement of population is one of the reasons for IMP malaria infections in the endemic areas. In addition, about 2400 men, mostly young adults aged 20–29 years, migrated between Brazilian states in 2005–2010 [29]. Similar results for both endemic and non-endemic regions were found in other published studies [30–38]. Furthermore, men are expected to have a higher rate of AU infections because they tend to visit native forests more often than women [39, 40]. Knowledge of these variables is critical to identifying the populations at risk and increasing awareness among medical professionals.

**Fig. 12:**
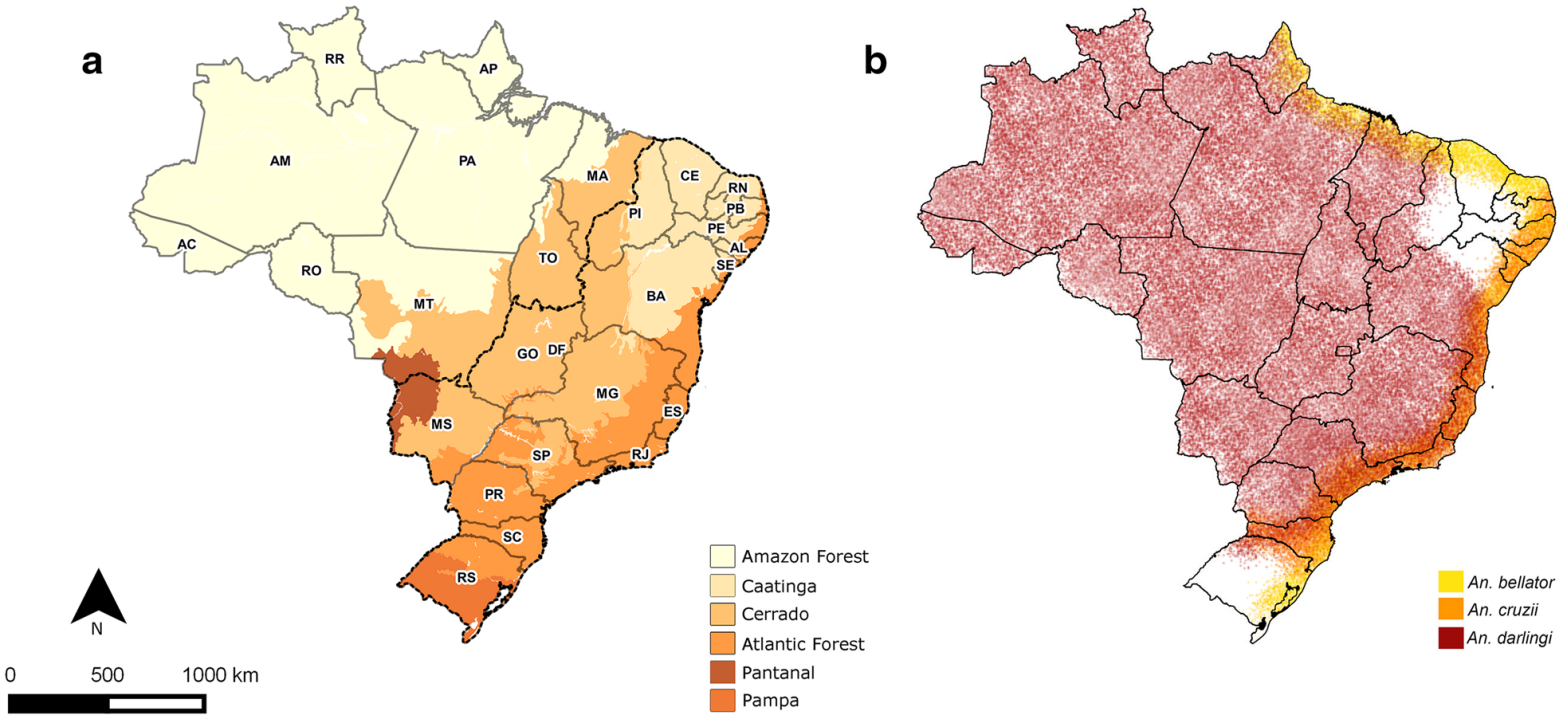
The Brazilian biome map (**a**) and geographical distribution of major malaria vectors in Brazil (**b**). Modified from IBGE [13] and Sinka et al. [53]

It was observed that IMP and AU/IN cases have a similar *Plasmodium* type infection pattern in all the analysed regions. This is probably because individuals with IMP malaria are generally asymptomatic and serve as *Plasmodium* reservoirs in regions that harbour the mosquito vector. This allows the parasite to maintain its lifecycle and be transmitted to other humans through this mosquito species, giving rise to AU malaria cases. The presence of such a cycle is evident from the large overlap between the specific areas of IMP and AU/IN cases when compared simultaneously. Bromeliad malaria is mainly concentrated in the Atlantic Forest region where the *Kerteszia* subgenus is responsible for infections (Fig. 12). According to the literature, monkey species in some of these regions may also serve as reservoirs of *Plasmodium* [26, 27, 41]. On the other hand, the vector *An. darlingi* is primarily responsible for IN malaria outbreaks in most regions of Brazil owing to its high transmission capacity and distribution throughout about 80 % of the country [9].

The overall mortality rate fluctuated between 2007 and 2014 and did not follow the distribution of malaria cases. Some municipalities had high malaria mortality rates, which may be due to lack of experience in the health community to diagnose malaria in non-endemic areas [42–45]. The vast majority of deaths were due to *P. falciparum*, which causes the most dangerous type of infection [46]. There was a peak in deaths due to *P. vivax* in 2011 that may be related to the delay in disease detection and/or treatment. Furthermore, in the last 10 years, a pattern of unusual clinical complications and fatal cases associated with *P. vivax* has been reported in Brazil [9] and should be investigated.

### *Plasmodium* species and their distribution

In this study an unusual pattern of AU/IN infections predominantly by *P. falciparum* in the Northeast and Southeast regions was observed. One possible explanation for this fact may be under-reporting by *P. vivax*. Since infections by *P. vivax* are generally benign and often asymptomatic, patients do not seek medical attention. Furthermore, especially in the extra-Amazonian regions, febrile malaria caused by *P. vivax* is often mistaken for other diseases (e.g. dengue) [12] and is not reported correctly. Thus, the number of *P. vivax* infections may be underestimated, which may obscure the actual scenario that occurs in the extra-Amazonian regions.

On the other hand, an unexpectedly high prevalence of *P. falciparum* was recently found in the blood of asymptomatic donors living in the southeast Brazilian Atlantic forest [47]. Additionally, Laporta et al. [48] showed that *P. falciparum* actively circulates, in higher proportion than *P. vivax*, among *Anopheles* mosquitoes in parts of the southeast Brazilian Atlantic forest. Therefore, a high rate of *P. falciparum* transmission by *Kerteszia* mosquitoes may challenge the classical bromeliad-malaria paradigm.

Almost all cases reported in 2007–2014 in the south were caused by *P. vivax*. PR had a high rate of AU malaria, with a high-risk cluster situated in the region of Foz do Iguaçu. Although the other southern states (SC and RS) had no cases of AU malaria in the study period, this does not exclude the possibility that malaria of this type occurred in these states. Asymptomatic patients with undiagnosed and untreated *Plasmodium* malaria have been detected in Atlantic Forest areas, which may allow maintenance of the parasite in this region [42, 49]. In line with these facts is the current presence of vector species circulating in those locations and capable of contributing to a possible outbreak.

The frequency of both IMP and AU/IN malaria caused by *P. falciparum* and *P. vivax* has been decreasing in the extra-Amazonian regions since 2007. Cases of double-infection are concentrated mainly in the mid-west, and their numbers remain constant. Future climate change may influence the distribution of the disease, which is dependent on the distribution of *Anopheles* mosquitoes that are competent of transmitting *P. falciparum* [50]. Moreover, the status of species not previously incriminated as vectors can change with changes in the landscape of the region, such as deforestation and rising temperatures. For example, the hitherto neglected epidemiological importance of *albitarsis* complex members in malaria transmission in South America might be to increase in the next years it [50].

### Temporal epidemiology

There was a significant decrease in both IMP and AU/IN cases in the extra-Amazonian regions in the last years. Nevertheless, some regions in PI had high incidence of IMP malaria. Chagas et al. [51] found that most of these cases were imported from Suriname or Maranhão (Amazonian region). Another cluster that often had a high malaria rate was observed in the region of Foz do Iguaçu, PR. This region is home to the Itaipu hydroelectric plant and, according to Ferreira [52], the following factors contributed to the occurrence of AU malaria there: (1) presence of cities or localities situated near the reservoir and other water bodies suitable for breeding of *An. darlingi*; (2) professional activities and recreational fishing that began after the impoundment; and (3) housing types (i.e. huts and wooden buildings) that allow easy access of mosquitoes. It is important to emphasize that PR had the highest percentage of international immigrants during the last 10 years in Brazil [29], which could have contributed to the increase in IMP malaria cases. The Pantanal biome region (see Fig. 12), which covers MS, also contains high-risk clusters of malaria because it is a highly preserved area that hosts the vector *An. darlingi* [53].

Although the numbers of malaria cases are relatively low, the disease is still present in all extra-Amazonian regions of Brazil. For example, an unusual increase in AU cases has been recently documented in the Rio de Janeiro state [54]. A surveillance system should be established and prepared to successfully overcome challenges associated with asymptomatic or oligosymptomatic *Plasmodium* infections [55] occurring in these regions. Since the 1980s, there has been a need for comprehensive serological studies in some areas with high-risk clusters [56]. Preventive active search may be a tool for the epidemiological surveillance of AU malaria. Continuous progress in epidemiological surveillance is necessary in these extra-Amazonian areas to assess vulnerability and susceptibility in different regions and enable rapid diagnosis and treatment across the healthcare network [57]. In addition to mapping risk areas, it will be useful to re-evaluate vector species that potentially contribute to the transmission because continuous reintroduction of *Plasmodium* and malaria is expected in controlled areas.

## Conclusions

Cases of malaria were detected between 2007 and 2014 throughout the extra-Amazonian regions of Brazil, which are vulnerable owing to climatic conditions and the presence of competent vectors. The number of IMP cases was higher than that of AU cases because of more active human migration. AU/IN cases were linked to the native forests, which serve as breeding sites of *Anopheles*, or related to outbreaks from IMP cases.

The observed unusual pattern of AU/IN infections predominantly by *P. falciparum* may be due to under-reporting of *P. vivax* malaria or a high rate of *P. falciparum* transmission by *Kerteszia* mosquitoes, which challenges the classical bromeliad-malaria paradigm. IMP and AU/IN cases had similar *Plasmodium* type infection patterns in all the analysed regions, probably because individuals with IMP malaria are generally asymptomatic and serve as *Plasmodium* reservoirs in regions that harbour the mosquito vector.

In the extra-Amazonian regions, malaria has become a problem that mainly affects isolated subpopulations with certain social characteristics (e.g. housing types) or professional activities. Therefore, adequate education of individuals at risk and health professionals is necessary. In addition, means of rapid diagnosis should be implemented in these regions to prevent serious adverse events or deaths from malaria.

## Additional file

10.1186/s12936-015-0934-6 Raw data from Notifiable Diseases Information System (SINAN) used in all analyses of this study.
