# Supplementary material for: Spatial and temporal epidemiology of malaria in extra-Amazonian regions of Brazil
Source: Malar J. 2015 Oct 15;14:408. doi: 10.1186/s12936-015-0934-6 (PMC4607178; doi:10.1186/s12936-015-0934-6)
Supplement: Supplementary file 1 — 10.1186/s12936-015-0934-6 Raw data from Notifiable Diseases Information System (SINAN) used in all analyses of this study. [file 12936_2015_934_MOESM1_ESM.pdf]

http://dtr2004.saude.gov.br/sinanweb/tabnet/dh?sinannet/malaria/bases/malabrnet.def

Info accessed August 2015.

MALÁRIA - Casos confirmados Notificados no Sistema de Informação de Agravos de Notificação - Sinan Net

Casos confirmados por Mun Infecção e result.parasitológi

Autoctone Mun Res: Sim

Período:2007-2014

Mun Infecção	Falciparum	F+FG	Vivax	F+V	Total
110001 Alta Floresta D'Oeste	0	0	1	0	1
110037 Alto Alegre dos Parecis	0	2	0	0	2
110040 Alto Paraíso	0	2	0	0	2
110002 Ariquemes	1	7	6	3	17
110045 Buritis	1	2	1	1	5
110004 Cacoal	0	0	0	1	1
110070 Campo Novo de Rondônia	1	0	0	0	1
110080 Candeias do Jamari	2	3	3	0	8
110094 Cujubim	0	0	2	0	2
110010 Guajará-Mirim	0	1	1	0	2
110011 Jaru	0	2	0	1	3
110012 Ji-Paraná	1	5	1	2	9
110013 Machadinho D'Oeste	0	3	4	0	7
110140 Monte Negro	0	1	0	0	1
110033 Nova Mamoré	0	3	0	1	4
110015 Ouro Preto do Oeste	0	0	1	0	1
110020 Porto Velho	30	31	39	22	122
110025 Presidente Médici	0	1	0	0	1
110026 Rio Crespo	0	0	0	1	1
110028 Rolim de Moura	0	3	1	0	4
110150 Seringueiras	0	1	0	0	1
110160 Theobroma	0	0	1	0	1
110175 Vale do Anari	0	1	0	0	1
110180 Vale do Paraíso	0	1	0	0	1
110030 Vilhena	0	0	3	1	4
110000 Município ignorado - RO	0	1	0	0	1
120001 Acrelândia	0	1	1	0	2
120020 Cruzeiro do Sul	0	4	1	0	5
120033 Mâncio Lima	0	1	0	0	1
120040 Rio Branco	0	0	2	2	4
120000 Município ignorado - AC	0	0	1	0	1
130014 Apuí	0	2	1	0	3
130020 Atalaia do Norte	0	1	0	0	1
130040 Barcelos	1	0	0	0	1
130070 Boca do Acre	0	3	1	1	5
130100 Carauari	0	0	0	1	1
130165 Guajará	0	0	0	1	1
130170 Humaitá	0	0	2	0	2
130240 Lábrea	0	1	0	0	1
130260 Manaus	13	16	5	7	41
130270 Manicoré	0	1	0	0	1
130300 Nhamundá	0	0	1	0	1
130330 Novo Aripuanã	0	1	0	0	1
130350 Pauini	0	5	1	1	7

130353 Presidente Figueiredo	0	1	0	0	1
130356 Rio Preto da Eva	1	0	0	0	1
130380 São Gabriel da Cachoeira	0	2	1	1	4
130406 Tabatinga	0	0	0	1	1
130420 Tefé	0	1	0	0	1
130426 Uarini	0	1	0	0	1
140010 Boa Vista	7	2	3	2	14
140017 Cantá	0	1	0	0	1
140020 Caracaraí	0	0	1	0	1
150060 Altamira	1	1	0	1	3
150085 Anapu	0	0	0	1	1
150140 Belém	3	1	2	2	8
150172 Brasil Novo	1	0	0	0	1
150295 Eldorado dos Carajás	0	0	0	1	1
150309 Goianésia do Pará	1	2	0	0	3
150360 Itaituba	4	3	8	3	18
150370 Itupiranga	1	0	0	0	1
150380 Jacundá	1	0	0	0	1
150420 Marabá	1	0	0	0	1
150497 Nova Ipixuna	1	0	0	0	1
150503 Novo Progresso	0	1	1	2	4
150506 Novo Repartimento	1	0	0	0	1
150548 Pacajá	1	2	0	1	4
150550 Paragominas	1	1	1	0	3
150553 Parauapebas	1	0	0	0	1
150680 Santarém	0	0	0	1	1
150740 São Francisco do Pará	1	0	0	0	1
150795 Tailândia	0	0	0	1	1
150805 Trairão	0	0	1	0	1
150808 Tucumã	0	0	0	1	1
150810 Tucuruí	1	0	0	1	2
150815 Uruará	0	1	0	0	1
150840 Xinguara	0	0	0	1	1
160010 Amapá	1	0	0	0	1
160030 Macapá	3	2	0	0	5
160040 Mazagão	0	0	0	2	2
160050 Oiapoque	2	1	0	1	4
160015 Pedra Branca do Amapari	0	0	1	1	2
160060 Santana	0	1	0	0	1
210095 Arame	0	1	0	0	1
210180 Benedito Leite	2	0	0	0	2
210330 Codó	1	0	0	0	1
210360 Coroatá	2	0	0	0	2
210380 Dom Pedro	1	0	0	0	1
210390 Duque Bacelar	1	0	0	0	1
210542 Itinga do Maranhão	0	0	0	1	1
210550 João Lisboa	1	0	0	0	1
210560 Joselândia	1	0	0	0	1
210570 Lago da Pedra	2	0	0	0	2
210596 Lagoa Grande do Maranhão	2	0	0	0	2

210600 Lima Campos	1	0	0	0	1
210632 Maracaçumé	1	0	0	0	1
210660 Matões	1	0	0	0	1
210810 Paulo Ramos	0	0	0	1	1
210820 Pedreiras	1	0	0	0	1
210845 Peritoró	1	0	0	0	1
210860 Pinheiro	0	1	0	0	1
210900 Porto Franco	0	1	0	0	1
210910 Presidente Dutra	1	0	0	0	1
210990 Santa Inês	1	0	0	0	1
211003 Santa Luzia do Paruá	1	0	0	0	1
211070 São Domingos do Maranhão	1	0	0	0	1
211090 São Francisco do Maranhão	1	0	0	0	1
211110 São João dos Patos	1	0	0	0	1
211125 São José dos Basílios	1	0	0	0	1
211174 Senador Alexandre Costa	1	0	0	0	1
211220 Timon	1	0	0	0	1
211223 Trizidela do Vale	1	0	0	0	1
211230 Tuntum	1	0	0	0	1
220194 Boqueirão do Piauí	1	0	0	0	1
220196 Brasileira	1	0	0	0	1
220200 Buriti dos Lopes	20	0	0	0	20
220213 Campo Grande do Piauí	1	0	0	0	1
220217 Campo Largo do Piauí	13	0	0	0	13
220253 Caraúbas do Piauí	1	0	0	0	1
220560 Landri Sales	1	0	0	0	1
220580 Luzilândia	17	0	0	0	17
220585 Madeiro	1	0	0	0	1
220610 Matias Olímpio	4	0	0	0	4
220620 Miguel Alves	2	0	0	0	2
220790 Pedro II	1	0	0	0	1
220800 Picos	1	0	0	0	1
220840 Piripiri	1	0	0	0	1
220850 Porto	3	1	0	0	4
220920 Santa Filomena	1	0	0	0	1
220980 São Gonçalo do Piauí	2	0	0	0	2
221005 São José do Divino	1	0	0	0	1
221100 Teresina	2	0	0	0	2
221120 Uruçuí	12	0	0	0	12
221150 Vera Mendes	1	0	0	0	1
230560 Independência	1	0	0	0	1
240810 Natal	2	0	0	0	2
241120 Santa Cruz	1	0	0	0	1
241200 São Gonçalo do Amarante	1	0	0	0	1
241340 Serra Negra do Norte	1	0	0	0	1
260300 Cabrobó	1	0	0	0	1
260345 Camaragibe	1	0	0	0	1
260960 Olinda	1	0	0	0	1
260980 Orocó	1	0	0	0	1
261070 Paulista	1	0	0	0	1

261160 Recife	3	0	0	0	3
290687 Capim Grosso	1	0	0	0	1
292100 Mata de São João	2	0	0	0	2
292740 Salvador	1	0	0	0	1
293015 Serra do Ramalho	1	0	0	0	1
320010 Afonso Cláudio	0	1	0	0	1
320030 Alfredo Chaves	0	23	0	0	23
320040 Anchieta	0	1	0	0	1
320060 Aracruz	0	1	0	0	1
320090 Barra de São Francisco	0	14	0	0	14
320130 Cariacica	0	1	0	0	1
320150 Colatina	0	1	0	0	1
320190 Domingos Martins	0	49	0	0	49
320210 Ecoporanga	0	3	0	0	3
320220 Fundão	0	3	0	0	3
320240 Guarapari	0	2	0	0	2
320250 Ibiraçu	0	11	0	0	11
320290 Itarana	0	3	0	0	3
320313 João Neiva	0	3	0	0	3
320320 Linhares	0	1	0	0	1
320334 Marechal Floriano	0	16	0	0	16
320390 Nova Venécia	0	13	0	0	13
320435 Rio Bananal	0	1	0	0	1
320450 Santa Leopoldina	0	25	0	0	25
320455 Santa Maria de Jetibá	0	11	0	0	11
320460 Santa Teresa	0	57	0	0	57
320470 São Gabriel da Palha	0	5	0	0	5
320495 São Roque do Canaã	0	4	0	0	4
320501 Sooretama	0	2	0	0	2
320503 Vargem Alta	0	1	0	0	1
320506 Venda Nova do Imigrante	0	2	0	0	2
320510 Viana	0	6	0	0	6
330080 Cachoeiras de Macacu	0	2	0	0	2
330140 Conceição de Macabu	0	1	0	0	1
330240 Macaé	0	4	0	0	4
330340 Nova Friburgo	0	11	0	0	11
330420 Resende	0	2	0	0	2
330455 Rio de Janeiro	0	4	0	0	4
330460 Santa Maria Madalena	0	2	0	0	2
350160 Americana	0	1	0	0	1
350570 Barueri	0	1	0	0	1
350635 Bertioga	0	12	0	0	12
350950 Campinas	0	1	0	0	1
351350 Cubatão	0	1	0	0	1
351535 Euclides da Cunha Paulista	0	1	0	0	1
352030 Iguape	0	1	0	0	1
352040 Ilhabela	0	2	0	0	2
352210 Itanhaém	0	14	0	0	14
352570 José Bonifácio	0	1	0	0	1
352620 Juquitiba	0	14	0	0	14

352770 Luiziânia	0	1	0	0	1
352940 Mauá	0	1	0	0	1
352990 Miracatu	0	2	0	0	2
353070 Mogi Guaçu	0	1	0	0	1
353260 Nhandeara	0	1	0	0	1
353720 Pedro de Toledo	0	1	0	0	1
354220 Rancharia	0	1	0	0	1
354340 Ribeirão Preto	1	0	0	0	1
354580 Santa Bárbara d'Oeste	0	1	0	0	1
354870 São Bernardo do Campo	0	6	0	0	6
354980 São José do Rio Preto	0	1	0	0	1
354990 São José dos Campos	0	1	0	0	1
355030 São Paulo	0	30	0	0	30
355070 São Sebastião	0	4	0	0	4
355180 Sete Barras	0	1	0	0	1
355580 Urânia	0	2	0	0	2
410345 Cafelândia	0	0	0	1	1
410640 Cornélio Procópio	0	0	1	0	1
410670 Cruzeiro do Sul	0	0	2	0	2
410690 Curitiba	0	0	2	0	2
410765 Fazenda Rio Grande	0	0	1	0	1
410830 Foz do Iguaçu	0	0	56	0	56
411095 Itaipulândia	0	0	1	0	1
411460 Marechal Cândido Rondon	0	0	1	0	1
411570 Matinhos	0	0	1	0	1
412570 São Miguel do Iguaçu	0	0	79	0	79
410000 Município ignorado - PR	1	0	0	0	1
420320 Camboriú	0	0	1	0	1
420540 Florianópolis	0	0	0	1	1
421010 Mafra	0	0	1	0	1
500070 Anastácio	0	0	0	1	1
500260 Camapuã	0	2	0	0	2
500270 Campo Grande	0	0	0	3	3
500600 Nova Alvorada do Sul	0	0	0	1	1
500720 Rio Brilhante	0	1	0	0	1
500790 Sidrolândia	0	0	0	1	1
510020 Água Boa	0	0	0	1	1
510025 Alta Floresta	0	0	1	0	1
510325 Colniza	1	0	1	1	3
510340 Cuiabá	0	1	1	2	4
510410 Guarantã do Norte	0	0	1	0	1
510515 Juína	0	0	1	1	2
510525 Lucas do Rio Verde	0	0	1	0	1
510625 Nova Xavantina	0	0	0	1	1
510760 Rondonópolis	1	0	0	0	1
510795 Tangará da Serra	0	1	0	0	1
520110 Anápolis	0	0	0	1	1
520215 Araguapaz	0	0	0	1	1
520425 Cachoeira Dourada	0	0	0	1	1
520450 Caldas Novas	0	0	0	3	3

520510 Catalão	0	0	0	1	1
520870 Goiânia	0	0	0	7	7
520915 Gouvelândia	0	0	0	1	1
521020 Iporá	0	0	0	1	1
521295 Matrinchã	0	0	0	1	1
521850 Quirinópolis	0	0	0	1	1
522020 São Miguel do Araguaia	0	0	0	2	2
530010 Brasília	1	0	0	0	1
000000 Ignorado ou exterior	1	0	0	0	1
Total	225	528	251	106	1110

MALÁRIA - Casos confirmados Notificados no Sistema de Informação de Agravos de Notificação - Sinan Net

Casos confirmados por Mun Infecção e result.parasitológi
Autoctone Mun Res: Sim
Período:2007

Mun Infecção	Falciparum	F+FG	Vivax	F+V	Total
110040 Alto Paraíso	0	2	0	0	2
110002 Ariquemes	0	3	1	0	4
110045 Buritis	0	0	0	1	1
110080 Candeias do Jamari	0	0	1	0	1
110094 Cujubim	0	0	1	0	1
110010 Guajará-Mirim	0	0	1	0	1
110011 Jaru	0	1	0	0	1
110012 Ji-Paraná	0	1	0	0	1
110013 Machadinho D'Oeste	0	1	0	0	1
110033 Nova Mamoré	0	0	0	1	1
110020 Porto Velho	4	2	6	8	20
110028 Rolim de Moura	0	1	1	0	2
110160 Theobroma	0	0	1	0	1
120001 Acrelândia	0	0	1	0	1
120020 Cruzeiro do Sul	0	1	0	0	1
120040 Rio Branco	0	0	1	2	3
130014 Apuí	0	1	1	0	2
130100 Carauari	0	0	0	1	1
130165 Guajará	0	0	0	1	1
130260 Manaus	1	3	2	3	9
130353 Presidente Figueiredo	0	1	0	0	1
130356 Rio Preto da Eva	1	0	0	0	1
150360 Itaituba	2	0	0	0	2
150370 Itupiranga	1	0	0	0	1
150506 Novo Repartimento	1	0	0	0	1
150740 São Francisco do Pará	1	0	0	0	1
160010 Amapá	1	0	0	0	1
160050 Oiapoque	1	0	0	0	1
210360 Coroatá	2	0	0	0	2
210390 Duque Bacelar	1	0	0	0	1
210560 Joselândia	1	0	0	0	1
220560 Landri Sales	1	0	0	0	1
220580 Luzilândia	1	0	0	0	1
220840 Piripiri	1	0	0	0	1
261070 Paulista	1	0	0	0	1
320030 Alfredo Chaves	0	3	0	0	3
320190 Domingos Martins	0	11	0	0	11
320334 Marechal Floriano	0	2	0	0	2
320390 Nova Venécia	0	1	0	0	1
320450 Santa Leopoldina	0	4	0	0	4
320460 Santa Teresa	0	7	0	0	7
320495 São Roque do Canaã	0	2	0	0	2
320501 Sooretama	0	2	0	0	2

320510 Viana	0	1	0	0	1
330340 Nova Friburgo	0	2	0	0	2
330455 Rio de Janeiro	0	1	0	0	1
350570 Barueri	0	1	0	0	1
352620 Juquitiba	0	9	0	0	9
353070 Mogi Guaçu	0	1	0	0	1
355030 São Paulo	0	21	0	0	21
355180 Sete Barras	0	1	0	0	1
410345 Cafelândia	0	0	0	1	1
410830 Foz do Iguaçu	0	0	44	0	44
411095 Itaipulândia	0	0	1	0	1
411460 Marechal Cândido Rondon	0	0	1	0	1
411570 Matinhos	0	0	1	0	1
412570 São Miguel do Iguaçu	0	0	7	0	7
420540 Florianópolis	0	0	0	1	1
500070 Anastácio	0	0	0	1	1
510025 Alta Floresta	0	0	1	0	1
510325 Colniza	1	0	0	0	1
510625 Nova Xavantina	0	0	0	1	1
510795 Tangará da Serra	0	1	0	0	1
520450 Caldas Novas	0	0	0	1	1
520870 Goiânia	0	0	0	1	1
Total	22	87	72	23	204

MALÁRIA - Casos confirmados Notificados no Sistema de Informação de Agravos de Notificação - Sinan Net
Casos confirmados por Mun Infecção e result.parasitológi
Autoctone Mun Res: Sim
Período:2008

Mun Infecção	Falciparum	F+FG	Vivax	F+V	Total
110001 Alta Floresta D'Oeste	0	0	1	0	1
110002 Ariquemes	0	1	0	1	2
110045 Buritis	0	1	1	0	2
110080 Candeias do Jamari	0	2	0	0	2
110011 Jaru	0	1	0	1	2
110012 Ji-Paraná	0	1	0	0	1
110013 Machadinho D'Oeste	0	1	0	0	1
110020 Porto Velho	3	5	4	5	17
110026 Rio Crespo	0	0	0	1	1
110180 Vale do Paraíso	0	1	0	0	1
110030 Vilhena	0	0	0	1	1
110000 Município ignorado - RO	0	1	0	0	1
120001 Acrelândia	0	1	0	0	1
120040 Rio Branco	0	0	1	0	1
130260 Manaus	3	0	0	0	3
130270 Manicoré	0	1	0	0	1
130380 São Gabriel da Cachoeira	0	0	0	1	1
130406 Tabatinga	0	0	0	1	1
140010 Boa Vista	0	1	0	0	1
150140 Belém	0	0	0	1	1
150295 Eldorado dos Carajás	0	0	0	1	1
150309 Goianésia do Pará	0	1	0	0	1
150808 Tucumã	0	0	0	1	1
150810 Tucuruí	0	0	0	1	1
160050 Oiapoque	0	0	0	1	1
210380 Dom Pedro	1	0	0	0	1
210596 Lagoa Grande do Maranhão	1	0	0	0	1
210860 Pinheiro	0	1	0	0	1
211174 Senador Alexandre Costa	1	0	0	0	1
211220 Timon	1	0	0	0	1
220200 Buriti dos Lopes	1	0	0	0	1
220580 Luzilândia	1	0	0	0	1
220610 Matias Olímpio	1	0	0	0	1
220850 Porto	1	0	0	0	1
221100 Teresina	2	0	0	0	2
260345 Camaragibe	1	0	0	0	1
261160 Recife	3	0	0	0	3
292100 Mata de São João	2	0	0	0	2
320030 Alfredo Chaves	0	4	0	0	4
320040 Anchieta	0	1	0	0	1
320130 Cariacica	0	1	0	0	1
320190 Domingos Martins	0	16	0	0	16
320220 Fundão	0	3	0	0	3

320250 Ibiraçu	0	1	0	0	1
320290 Itarana	0	1	0	0	1
320313 João Neiva	0	1	0	0	1
320320 Linhares	0	1	0	0	1
320334 Marechal Floriano	0	4	0	0	4
320390 Nova Venécia	0	1	0	0	1
320450 Santa Leopoldina	0	11	0	0	11
320455 Santa Maria de Jetibá	0	5	0	0	5
320460 Santa Teresa	0	15	0	0	15
320495 São Roque do Canaã	0	1	0	0	1
320510 Viana	0	3	0	0	3
330340 Nova Friburgo	0	4	0	0	4
352030 Iguape	0	1	0	0	1
352620 Juquitiba	0	3	0	0	3
352990 Miracatu	0	2	0	0	2
410690 Curitiba	0	0	2	0	2
410765 Fazenda Rio Grande	0	0	1	0	1
410830 Foz do Iguaçu	0	0	7	0	7
412570 São Miguel do Iguaçu	0	0	2	0	2
510325 Colniza	0	0	0	1	1
510340 Cuiabá	0	0	0	1	1
510410 Guarantã do Norte	0	0	1	0	1
510515 Juína	0	0	1	1	2
510525 Lucas do Rio Verde	0	0	1	0	1
520215 Araguapaz	0	0	0	1	1
Total	22	98	22	20	162

MALÁRIA - Casos confirmados Notificados no Sistema de Informação de Agravos de Notificação - Sinan Net
Casos confirmados por Mun Infecção e result.parasitológi
Autoctone Mun Res: Sim
Período:2009

Mun Infecção	Falciparum	F+FG	Vivax	F+V	Total
110002 Ariquemes	0	1	1	1	3
110004 Cacoal	0	0	0	1	1
110070 Campo Novo de Rondônia	1	0	0	0	1
110080 Candeias do Jamari	0	0	1	0	1
110094 Cujubim	0	0	1	0	1
110013 Machadinho D'Oeste	0	0	1	0	1
110140 Monte Negro	0	1	0	0	1
110020 Porto Velho	0	5	3	1	9
110025 Presidente Médici	0	1	0	0	1
110150 Seringueiras	0	1	0	0	1
110030 Vilhena	0	0	1	0	1
120020 Cruzeiro do Sul	0	1	0	0	1
120000 Município ignorado - AC	0	0	1	0	1
130070 Boca do Acre	0	1	0	0	1
130260 Manaus	5	2	1	1	9
130330 Novo Aripuanã	0	1	0	0	1
140010 Boa Vista	0	0	1	0	1
140020 Caracaraí	0	0	1	0	1
150085 Anapu	0	0	0	1	1
150140 Belém	0	0	0	1	1
150360 Itaituba	0	0	1	0	1
150420 Marabá	1	0	0	0	1
150497 Nova Ipixuna	1	0	0	0	1
150548 Pacajá	0	0	0	1	1
150550 Paragominas	0	0	1	0	1
150795 Tailândia	0	0	0	1	1
150810 Tucuruí	1	0	0	0	1
150815 Uruará	0	1	0	0	1
160030 Macapá	1	0	0	0	1
160015 Pedra Branca do Amapari	0	0	0	1	1
210542 Itinga do Maranhão	0	0	0	1	1
210550 João Lisboa	1	0	0	0	1
210910 Presidente Dutra	1	0	0	0	1
211003 Santa Luzia do Paruá	1	0	0	0	1
211090 São Francisco do Maranhão	1	0	0	0	1
220253 Caraúbas do Piauí	1	0	0	0	1
220580 Luzilândia	2	0	0	0	2
220585 Madeiro	1	0	0	0	1
260980 Orocó	1	0	0	0	1
292740 Salvador	1	0	0	0	1
320060 Aracruz	0	1	0	0	1
320190 Domingos Martins	0	6	0	0	6
320290 Itarana	0	2	0	0	2
320334 Marechal Floriano	0	2	0	0	2
320450 Santa Leopoldina	0	2	0	0	2

320455 Santa Maria de Jetibá	0	1	0	0	1
320460 Santa Teresa	0	4	0	0	4
320495 São Roque do Canaã	0	1	0	0	1
330455 Rio de Janeiro	0	3	0	0	3
350160 Americana	0	1	0	0	1
351535 Euclides da Cunha Paulista	0	1	0	0	1
352210 Itanhaém	0	14	0	0	14
352940 Mauá	0	1	0	0	1
353260 Nhandeara	0	1	0	0	1
353720 Pedro de Toledo	0	1	0	0	1
354220 Rancharia	0	1	0	0	1
354340 Ribeirão Preto	1	0	0	0	1
354580 Santa Bárbara d'Oeste	0	1	0	0	1
354980 São José do Rio Preto	0	1	0	0	1
355030 São Paulo	0	5	0	0	5
355070 São Sebastião	0	1	0	0	1
410830 Foz do Iguaçu	0	0	1	0	1
412570 São Miguel do Iguaçu	0	0	23	0	23
410000 Município ignorado - PR	1	0	0	0	1
500270 Campo Grande	0	0	0	3	3
500790 Sidrolândia	0	0	0	1	1
520510 Catalão	0	0	0	1	1
521020 Iporá	0	0	0	1	1
530010 Brasília	1	0	0	0	1
Total	23	65	38	16	142

MALÁRIA - Casos confirmados Notificados no Sistema de Informação de Agravos de Notificação - Sinan Net

Casos confirmados por Mun Infecção e result.parasitológi
Autoctone Mun Res: Sim
Período:2010

Mun Infecção	Falciparum	F+FG	Vivax	F+V	Total
110037 Alto Alegre dos Parecis	0	2	0	0	2
110002 Ariquemes	0	1	2	1	4
110045 Buritis	0	1	0	0	1
110012 Ji-Paraná	1	1	1	0	3
110033 Nova Mamoré	0	1	0	0	1
110020 Porto Velho	8	4	4	2	18
110030 Vilhena	0	0	1	0	1
120020 Cruzeiro do Sul	0	1	1	0	2
130014 Apuí	0	1	0	0	1
130070 Boca do Acre	0	0	0	1	1
130260 Manaus	1	2	1	1	5
130300 Nhamundá	0	0	1	0	1
140010 Boa Vista	2	1	0	0	3
150060 Altamira	1	1	0	1	3
150309 Goianésia do Pará	1	1	0	0	2
150360 Itaituba	0	0	0	1	1
150380 Jacundá	1	0	0	0	1
150503 Novo Progresso	0	0	0	1	1
150680 Santarém	0	0	0	1	1
150840 Xinguara	0	0	0	1	1
160030 Macapá	1	0	0	0	1
210570 Lago da Pedra	1	0	0	0	1
210600 Lima Campos	1	0	0	0	1
210990 Santa Inês	1	0	0	0	1
220200 Buriti dos Lopes	18	0	0	0	18
220580 Luzilândia	9	0	0	0	9
220610 Matias Olímpio	1	0	0	0	1
220800 Picos	1	0	0	0	1
240810 Natal	1	0	0	0	1
260300 Cabrobó	1	0	0	0	1
260960 Olinda	1	0	0	0	1
290687 Capim Grosso	1	0	0	0	1
293015 Serra do Ramalho	1	0	0	0	1
320030 Alfredo Chaves	0	2	0	0	2
320090 Barra de São Francisco	0	13	0	0	13
320190 Domingos Martins	0	4	0	0	4
320210 Ecoporanga	0	3	0	0	3
320240 Guarapari	0	1	0	0	1
320250 Ibiraçu	0	4	0	0	4
320313 João Neiva	0	1	0	0	1
320334 Marechal Floriano	0	1	0	0	1
320435 Rio Bananal	0	1	0	0	1
320450 Santa Leopoldina	0	1	0	0	1

320460 Santa Teresa	0	12	0	0	12
330080 Cachoeiras de Macacu	0	1	0	0	1
330140 Conceição de Macabu	0	1	0	0	1
330240 Macaé	0	1	0	0	1
330340 Nova Friburgo	0	3	0	0	3
350950 Campinas	0	1	0	0	1
351350 Cubatão	0	1	0	0	1
352040 Ilhabela	0	2	0	0	2
352570 José Bonifácio	0	1	0	0	1
352770 Luiziânia	0	1	0	0	1
354990 São José dos Campos	0	1	0	0	1
355030 São Paulo	0	4	0	0	4
355070 São Sebastião	0	1	0	0	1
355580 Urânia	0	2	0	0	2
410830 Foz do Iguaçu	0	0	1	0	1
412570 São Miguel do Iguaçu	0	0	45	0	45
421010 Mafra	0	0	1	0	1
510020 Água Boa	0	0	0	1	1
520110 Anápolis	0	0	0	1	1
520425 Cachoeira Dourada	0	0	0	1	1
520450 Caldas Novas	0	0	0	2	2
520915 Gouvelândia	0	0	0	1	1
Total	53	80	58	16	207

MALÁRIA - Casos confirmados Notificados no Sistema de Informação de Agravos de Notificação - Sinan Net

Casos confirmados por Mun Infecção e result.parasitológi
Autoctone Mun Res: Sim
Período:2011

Mun Infecção	Falciparum	F+FG	Vivax	F+V	Total
110002 Ariquemes	1	0	1	0	2
110080 Candeias do Jamari	0	0	1	0	1
110013 Machadinho D'Oeste	0	0	1	0	1
110033 Nova Mamoré	0	2	0	0	2
110020 Porto Velho	6	4	4	2	16
130170 Humaitá	0	0	1	0	1
130260 Manaus	1	2	1	2	6
130426 Uarini	0	1	0	0	1
140010 Boa Vista	0	0	0	1	1
140017 Cantá	0	1	0	0	1
150140 Belém	1	0	2	0	3
150172 Brasil Novo	1	0	0	0	1
150360 Itaituba	0	0	1	0	1
150503 Novo Progresso	0	1	1	1	3
150548 Pacajá	0	1	0	0	1
150553 Parauapebas	1	0	0	0	1
160030 Macapá	1	0	0	0	1
160040 Mazagão	0	0	0	2	2
160050 Oiapoque	0	1	0	0	1
160015 Pedra Branca do Amapari	0	0	1	0	1
210095 Arame	0	1	0	0	1
210180 Benedito Leite	2	0	0	0	2
210632 Maracaçumé	1	0	0	0	1
211070 São Domingos do Maranhão	1	0	0	0	1
211125 São José dos Basílios	1	0	0	0	1
211223 Trizidela do Vale	1	0	0	0	1
220200 Buriti dos Lopes	1	0	0	0	1
220580 Luzilândia	1	0	0	0	1
221120 Uruçuí	12	0	0	0	12
221150 Vera Mendes	1	0	0	0	1
240810 Natal	1	0	0	0	1
241120 Santa Cruz	1	0	0	0	1
320030 Alfredo Chaves	0	7	0	0	7
320090 Barra de São Francisco	0	1	0	0	1
320190 Domingos Martins	0	3	0	0	3
320250 Ibiraçu	0	2	0	0	2
320334 Marechal Floriano	0	4	0	0	4
320450 Santa Leopoldina	0	3	0	0	3
320455 Santa Maria de Jetibá	0	1	0	0	1
320460 Santa Teresa	0	5	0	0	5
320503 Vargem Alta	0	1	0	0	1
320510 Viana	0	1	0	0	1
352620 Juquitiba	0	1	0	0	1

355070 São Sebastião	0	1	0	0	1
410830 Foz do Iguaçu	0	0	3	0	3
412570 São Miguel do Iguaçu	0	0	2	0	2
500260 Camapuã	0	1	0	0	1
500600 Nova Alvorada do Sul	0	0	0	1	1
510325 Colniza	0	0	1	0	1
510340 Cuiabá	0	1	0	0	1
510760 Rondonópolis	1	0	0	0	1
521295 Matrinchã	0	0	0	1	1
521850 Quirinópolis	0	0	0	1	1
Total	36	46	20	11	113

MALÁRIA - Casos confirmados Notificados no Sistema de Informação de Agravos de Notificação - Sinan Net

Casos confirmados por Mun Infecção e result.parasitológi
Autoctone Mun Res: Sim
Período:2012

Mun Infecção	Falciparum	F+FG	Vivax	F+V	Total
110045 Buritis	1	0	0	0	1
110080 Candeias do Jamari	2	0	0	0	2
110010 Guajará-Mirim	0	1	0	0	1
110012 Ji-Paraná	0	1	0	1	2
110013 Machadinho D'Oeste	0	1	1	0	2
110015 Ouro Preto do Oeste	0	0	1	0	1
110020 Porto Velho	4	3	6	1	14
110030 Vilhena	0	0	1	0	1
130260 Manaus	1	5	0	0	6
130420 Tefé	0	1	0	0	1
140010 Boa Vista	1	0	0	0	1
150140 Belém	1	1	0	0	2
150360 Itaituba	0	2	2	2	6
150550 Paragominas	1	1	0	0	2
160030 Macapá	0	1	0	0	1
160050 Oiapoque	1	0	0	0	1
210845 Peritoró	1	0	0	0	1
210900 Porto Franco	0	1	0	0	1
211230 Tuntum	1	0	0	0	1
220194 Boqueirão do Piauí	1	0	0	0	1
220196 Brasileira	1	0	0	0	1
220217 Campo Largo do Piauí	1	0	0	0	1
220580 Luzilândia	1	0	0	0	1
220610 Matias Olímpio	2	0	0	0	2
220620 Miguel Alves	2	0	0	0	2
220850 Porto	0	1	0	0	1
220980 São Gonçalo do Piauí	2	0	0	0	2
241200 São Gonçalo do Amarante	1	0	0	0	1
241340 Serra Negra do Norte	1	0	0	0	1
320030 Alfredo Chaves	0	1	0	0	1
320150 Colatina	0	1	0	0	1
320250 Ibiraçu	0	1	0	0	1
320390 Nova Venécia	0	10	0	0	10
320450 Santa Leopoldina	0	1	0	0	1
320460 Santa Teresa	0	3	0	0	3
320470 São Gabriel da Palha	0	5	0	0	5
320506 Venda Nova do Imigrante	0	1	0	0	1
330420 Resende	0	1	0	0	1
330460 Santa Maria Madalena	0	1	0	0	1
350635 Bertioga	0	11	0	0	11
410640 Cornélio Procópio	0	0	1	0	1
500260 Camapuã	0	1	0	0	1
510340 Cuiabá	0	0	1	0	1
000000 Ignorado ou exterior	1	0	0	0	1

Total	27	56	13	4	100

MALÁRIA - Casos confirmados Notificados no Sistema de Informação de Agravos de Notificação - Sinan Net

Casos confirmados por Mun Infecção e result.parasitológi

Autoctone Mun Res: Sim

Período:2013

Mun Infecção	Falciparum	F+FG	Vivax	F+V	Total
110002 Ariquemes	0	1	1	0	2
110080 Candeias do Jamari	0	1	0	0	1
110012 Ji-Paraná	0	1	0	1	2
110013 Machadinho D'Oeste	0	0	1	0	1
110020 Porto Velho	3	4	9	2	18
110028 Rolim de Moura	0	2	0	0	2
120020 Cruzeiro do Sul	0	1	0	0	1
130040 Barcelos	1	0	0	0	1
130260 Manaus	0	2	0	0	2
130380 São Gabriel da Cachoeira	0	0	1	0	1
140010 Boa Vista	0	0	0	1	1
150360 Itaituba	0	0	4	0	4
150548 Pacajá	0	1	0	0	1
150805 Trairão	0	0	1	0	1
210570 Lago da Pedra	1	0	0	0	1
210596 Lagoa Grande do Maranhão	1	0	0	0	1
210660 Matões	1	0	0	0	1
210820 Pedreiras	1	0	0	0	1
211110 São João dos Patos	1	0	0	0	1
220217 Campo Largo do Piauí	12	0	0	0	12
220580 Luzilândia	2	0	0	0	2
220790 Pedro II	1	0	0	0	1
220850 Porto	2	0	0	0	2
221005 São José do Divino	1	0	0	0	1
320030 Alfredo Chaves	0	3	0	0	3
320190 Domingos Martins	0	7	0	0	7
320240 Guarapari	0	1	0	0	1
320250 Ibiraçu	0	2	0	0	2
320334 Marechal Floriano	0	2	0	0	2
320390 Nova Venécia	0	1	0	0	1
320450 Santa Leopoldina	0	1	0	0	1
320455 Santa Maria de Jetibá	0	3	0	0	3
320460 Santa Teresa	0	6	0	0	6
320506 Venda Nova do Imigrante	0	1	0	0	1
320510 Viana	0	1	0	0	1
330240 Macaé	0	1	0	0	1
330340 Nova Friburgo	0	2	0	0	2
330420 Resende	0	1	0	0	1
350635 Bertioga	0	1	0	0	1
354870 São Bernardo do Campo	0	6	0	0	6
355070 São Sebastião	0	1	0	0	1
410670 Cruzeiro do Sul	0	0	2	0	2
420320 Camboriú	0	0	1	0	1

Total	27	53	20	4	104

MALÁRIA - Casos confirmados Notificados no Sistema de Informação de Agravos de Notificação - Sinan Net

Casos confirmados por Mun Infecção e result.parasitológi
Autoctone Mun Res: Sim
Período:2014

Mun Infecção	Falciparum	F+FG	Vivax	F+V	Total
110020 Porto Velho	2	4	3	1	10
110175 Vale do Anari	0	1	0	0	1
120033 Mâncio Lima	0	1	0	0	1
130020 Atalaia do Norte	0	1	0	0	1
130070 Boca do Acre	0	2	1	0	3
130170 Humaitá	0	0	1	0	1
130240 Lábrea	0	1	0	0	1
130260 Manaus	1	0	0	0	1
130350 Pauini	0	5	1	1	7
130380 São Gabriel da Cachoeira	0	2	0	0	2
140010 Boa Vista	4	0	2	0	6
150140 Belém	1	0	0	0	1
150360 Itaituba	2	1	0	0	3
150548 Pacajá	1	0	0	0	1
160030 Macapá	0	1	0	0	1
160060 Santana	0	1	0	0	1
210330 Codó	1	0	0	0	1
210810 Paulo Ramos	0	0	0	1	1
220213 Campo Grande do Piauí	1	0	0	0	1
220920 Santa Filomena	1	0	0	0	1
230560 Independência	1	0	0	0	1
320010 Afonso Cláudio	0	1	0	0	1
320030 Alfredo Chaves	0	3	0	0	3
320190 Domingos Martins	0	2	0	0	2
320250 Ibiraçu	0	1	0	0	1
320313 João Neiva	0	1	0	0	1
320334 Marechal Floriano	0	1	0	0	1
320450 Santa Leopoldina	0	2	0	0	2
320455 Santa Maria de Jetibá	0	1	0	0	1
320460 Santa Teresa	0	5	0	0	5
330080 Cachoeiras de Macacu	0	1	0	0	1
330240 Macaé	0	2	0	0	2
330460 Santa Maria Madalena	0	1	0	0	1
352620 Juquitiba	0	1	0	0	1
500720 Rio Brilhante	0	1	0	0	1
510340 Cuiabá	0	0	0	1	1
520870 Goiânia	0	0	0	6	6
522020 São Miguel do Araguaia	0	0	0	2	2
Total	15	43	8	12	78

Mortalidade - Brasil
Óbitos p/Residênc por Município e Ano do Óbito
Amazônia Legal: Não
Categoria CID-10: B50 Malaria p/Plasmodium falciparum
Causa - CID-BR-10: ... 024 Malária
Período:2007-2013

Município	2007	2008	2009	2010	2011	2012	2013	Total
240810 Natal	-	1	-	-	-	-	-	1
260005 Abreu e Lima	-	-	1	-	-	-	-	1
260000 Município ignorado - PE	-	-	1	-	-	-	-	1
292740 Salvador	-	-	-	-	1	-	-	1
293130 Tapiramutá	1	-	-	-	-	-	-	1
310620 Belo Horizonte	-	-	-	-	-	1	-	1
317010 Uberaba	-	-	-	-	1	-	-	1
320060 Aracruz	-	-	-	-	1	-	-	1
330330 Niterói	1	-	-	-	-	-	-	1
330455 Rio de Janeiro	1	-	-	-	-	1	-	2
352050 Indaiatuba	-	-	-	-	1	-	-	1
352440 Jacareí	1	-	-	-	-	-	-	1
354850 Santos	-	-	-	1	-	-	-	1
355220 Sorocaba	-	-	-	1	-	-	-	1
355710 Votuporanga	-	-	-	1	-	-	-	1
410660 Cruzeiro do Oeste	-	-	1	-	-	-	-	1
411370 Londrina	1	-	-	-	-	-	-	1
411990 Ponta Grossa	-	1	-	-	-	-	-	1
420490 Descanso	-	-	-	1	-	-	-	1
420540 Florianópolis	-	-	-	-	1	-	-	1
431480 Portão	-	-	-	1	-	-	-	1
500270 Campo Grande	-	-	-	1	-	-	-	1
520870 Goiânia	1	-	-	-	-	1	1	3
521800 Porangatu	1	-	-	-	-	-	-	1
530010 Brasília	-	-	-	-	-	-	1	1
Total	7	2	3	6	5	3	2	28

Fonte: MS/SVS/CGIAE - Sistema de Informações sobre Mortalidade - SIM
Nota:

Em 2011, houve uma mudança no conteúdo da Declaração de Óbito, com maior detalhamento das informações coletadas. Para este ano, foram utilizados simultaneamente os dois formulários. Para mais detalhes sobre as mudanças ocorridas e os seus efeitos, veja o documento "Sistema de Informações sobre Mortalidade - SIM. Consolidação da base de dados de 2011".

Mortalidade - Brasil
Óbitos p/Residênc por Município e Ano do Óbito
Amazônia Legal: Não
Categoria CID-10: B51 Malaria p/Plasmodium vivax
Causa - CID-BR-10: ... 024 Malária
Período:2007-2013

Município	2010	2011	2013	Total
220200 Buriti dos Lopes	-	1	-	1
320013 Águia Branca	-	1	-	1
352590 Jundiaí	-	1	-	1
355220 Sorocaba	-	-	1	1
411250 Jardim Alegre	-	1	-	1
421010 Mafra	1	-	-	1
Total	1	4	1	6

Fonte: MS/SVS/CGIAE - Sistema de Informações sobre Mortalidade - SIM
Nota:

Em 2011, houve uma mudança no conteúdo da Declaração de Óbito, com maior detalhamento das informações coletadas. Para este ano, foram utilizados simultaneamente os dois formulários. Para mais detalhes sobre as mudanças ocorridas e os seus efeitos, veja o documento "Sistema de Informações sobre Mortalidade - SIM. Consolidação da base de dados de 2011".

MALÁRIA - Casos confirmados Notificados no Sistema de Informação de Agravos de Notificação - Sinan Net

Casos confirmados por Munic. Residência e result.parasitológi
Autoctone Mun Res: Não
Período:2007-2014

Munic. Residência	Falciparum	F+FG	Vivax	F+V	Total
110001 Alta Floresta D'Oeste	0	1	1	0	2
110040 Alto Paraíso	0	0	1	0	1
110002 Ariquemes	0	2	2	4	8
110045 Buritis	0	0	2	0	2
110004 Cacoal	0	4	1	2	7
110070 Campo Novo de Rondônia	0	1	0	0	1
110080 Candeias do Jamari	0	2	1	0	3
110094 Cujubim	0	0	1	0	1
110009 Espigão D'Oeste	0	1	0	0	1
110011 Jaru	0	3	2	0	5
110012 Ji-Paraná	0	3	3	0	6
110013 Machadinho D'Oeste	0	1	1	0	2
110130 Mirante da Serra	0	1	0	0	1
110140 Monte Negro	0	1	0	0	1
110033 Nova Mamoré	0	2	0	0	2
110015 Ouro Preto do Oeste	0	1	0	0	1
110020 Porto Velho	6	16	3	6	31
110025 Presidente Médici	0	1	0	0	1
110026 Rio Crespo	0	0	1	0	1
110028 Rolim de Moura	0	0	1	0	1
110160 Theobroma	0	1	0	0	1
110170 Urupá	0	1	0	0	1
110030 Vilhena	0	0	0	3	3
120001 Acrelândia	0	0	1	0	1
120020 Cruzeiro do Sul	0	2	0	0	2
120080 Porto Acre	0	1	0	0	1
120040 Rio Branco	0	1	0	1	2
120042 Rodrigues Alves	0	0	0	1	1
130014 Apuí	0	1	1	0	2
130020 Atalaia do Norte	0	1	0	0	1
130110 Careiro	1	0	0	0	1
130260 Manaus	6	7	1	0	14
130270 Manicoré	0	1	0	0	1
130300 Nhamundá	0	0	1	0	1
130353 Presidente Figueiredo	1	0	0	0	1
130380 São Gabriel da Cachoeira	0	1	0	0	1
140010 Boa Vista	1	3	2	2	8
140047 Rorainópolis	0	1	0	0	1
140060 São Luiz	0	0	0	1	1
150080 Ananindeua	1	0	0	0	1
150085 Anapu	0	1	0	0	1
150130 Barcarena	0	1	0	0	1
150140 Belém	0	2	0	0	2

150157 Bom Jesus do Tocantins	1	0	0	0	1
150360 Itaituba	1	4	1	0	6
150380 Jacundá	0	1	1	1	3
150420 Marabá	0	1	0	0	1
150506 Novo Repartimento	1	0	0	0	1
150548 Pacajá	0	1	0	0	1
150550 Paragominas	1	0	0	0	1
150553 Parauapebas	0	1	0	0	1
150680 Santarém	0	0	0	1	1
150730 São Félix do Xingu	0	1	0	0	1
150840 Xinguara	0	1	0	0	1
160020 Calçoene	0	2	0	0	2
160030 Macapá	1	2	0	1	4
160050 Oiapoque	1	0	0	0	1
160005 Serra do Navio	0	0	0	1	1
170200 Araguaçu	0	0	0	2	2
172100 Palmas	1	0	0	0	1
210005 Açailândia	0	1	0	0	1
210043 Alto Alegre do Maranhão	1	0	0	0	1
210095 Arame	0	1	0	0	1
210120 Bacabal	1	0	0	0	1
210150 Barão de Grajaú	1	0	0	0	1
210160 Barra do Corda	1	0	0	0	1
210220 Buriti	1	0	0	0	1
210300 Caxias	1	0	0	0	1
210330 Codó	1	0	0	0	1
210360 Coroatá	1	0	0	0	1
210390 Duque Bacelar	2	0	0	0	2
210462 Governador Luiz Rocha	1	0	0	0	1
210530 Imperatriz	0	0	0	1	1
210570 Lago da Pedra	5	1	0	0	6
210596 Lagoa Grande do Maranhão	1	0	0	0	1
210745 Olinda Nova do Maranhão	0	1	0	0	1
210780 Parnarama	1	0	0	0	1
210820 Pedreiras	1	0	0	0	1
210910 Presidente Dutra	1	0	0	1	2
210980 Santa Helena	0	1	0	0	1
211000 Santa Luzia	1	0	0	0	1
211050 São Bento	0	1	0	0	1
211130 São Luís	1	3	0	0	4
211220 Timon	6	0	0	0	6
211230 Tuntum	1	0	0	0	1
211300 Vitorino Freire	2	0	0	0	2
211400 Zé Doca	0	0	1	0	1
210000 Município ignorado - MA	0	0	0	1	1
220020 Água Branca	1	0	0	0	1
220025 Alagoinha do Piauí	1	0	0	0	1
220040 Altos	1	0	0	0	1
220050 Amarante	3	0	0	0	3
220120 Barras	17	0	0	0	17

220130 Barreiras do Piauí	1	0	0	0	1
220140 Barro Duro	2	0	0	0	2
220150 Batalha	4	0	0	0	4
220173 Betânia do Piauí	1	0	0	0	1
220177 Boa Hora	1	0	0	0	1
220194 Boqueirão do Piauí	1	0	0	0	1
220196 Brasileira	2	0	0	0	2
220200 Buriti dos Lopes	0	1	0	0	1
220205 Cabeceiras do Piauí	3	0	0	0	3
220217 Campo Largo do Piauí	6	0	0	0	6
220220 Campo Maior	2	0	0	0	2
220230 Canto do Buriti	2	0	0	0	2
220240 Capitão de Campos	1	0	0	0	1
220330 Demerval Lobão	1	0	0	0	1
220370 Esperantina	5	0	0	0	5
220385 Floresta do Piauí	0	1	0	0	1
220390 Floriano	3	0	0	0	3
220420 Francisco Santos	1	0	0	0	1
220430 Fronteiras	1	0	0	0	1
220435 Geminiano	1	0	0	0	1
220450 Guadalupe	2	0	0	0	2
220510 Itaueira	1	0	0	0	1
220540 Joaquim Pires	5	0	0	0	5
220545 Joca Marques	7	0	0	0	7
220550 José de Freitas	1	0	0	0	1
220558 Lagoa do Piauí	1	0	0	0	1
220580 Luzilândia	72	0	0	0	72
220585 Madeiro	6	0	0	0	6
220610 Matias Olímpio	20	0	0	0	20
220620 Miguel Alves	1	0	0	0	1
220640 Monsenhor Gil	5	0	0	0	5
220650 Monsenhor Hipólito	5	0	0	0	5
220750 Palmeirais	2	0	0	0	2
220770 Parnaíba	2	0	0	0	2
220800 Picos	1	0	0	0	1
220840 Piripiri	23	0	0	0	23
220850 Porto	6	0	0	0	6
220880 Regeneração	9	0	0	0	9
220920 Santa Filomena	1	0	0	0	1
220930 Santa Luz	1	0	0	0	1
220980 São Gonçalo do Piauí	8	0	0	0	8
220997 São João do Arraial	1	0	0	0	1
221090 Socorro do Piauí	1	0	0	0	1
221100 Teresina	75	0	0	0	75
221110 União	1	0	0	0	1
221120 Uruçuí	6	0	0	0	6
221130 Valença do Piauí	1	0	0	0	1
221140 Várzea Grande	1	0	0	0	1
221160 Vila Nova do Piauí	1	0	0	0	1
220000 Município ignorado - PI	1	0	0	0	1

230075 Amontada	3	0	0	0	3
230110 Aracati	3	0	0	0	3
230240 Boa Viagem	5	0	0	0	5
230250 Brejo Santo	1	0	0	0	1
230260 Camocim	1	0	0	0	1
230280 Canindé	1	0	0	0	1
230340 Carnaubal	1	0	0	0	1
230370 Caucaia	10	0	0	0	10
230380 Cedro	2	0	0	0	2
230400 Coreaú	3	0	0	0	3
230410 Crateús	2	0	0	0	2
230425 Cruz	2	0	0	0	2
230440 Fortaleza	108	1	0	0	109
230445 Fortim	2	0	0	0	2
230470 Granja	1	0	0	0	1
230523 Horizonte	2	0	0	0	2
230550 Iguatu	2	0	0	0	2
230610 Irauçuba	5	0	0	0	5
230620 Itaiçaba	1	0	0	0	1
230630 Itapagé	2	0	0	0	2
230640 Itapipoca	3	0	0	0	3
230690 Jaguaribe	0	1	0	0	1
230730 Juazeiro do Norte	2	0	0	0	2
230760 Limoeiro do Norte	1	0	0	0	1
230830 Milagres	1	0	0	0	1
230870 Morada Nova	1	0	0	0	1
230960 Pacajus	1	0	0	0	1
231020 Paracuru	2	0	0	0	2
231030 Parambu	3	0	0	0	3
231050 Pedra Branca	1	0	0	0	1
231126 Quiterianópolis	2	0	0	0	2
231140 Quixeramobim	1	0	0	0	1
231150 Quixeré	1	0	0	0	1
231230 São Benedito	1	0	0	0	1
231240 São Gonçalo do Amarante	3	0	0	0	3
231280 Senador Sá	1	0	0	0	1
231290 Sobral	4	0	0	0	4
231300 Solonópole	2	0	0	0	2
231310 Tabuleiro do Norte	10	0	0	0	10
231335 Tejuçuoca	1	0	0	0	1
231350 Trairi	3	0	0	0	3
231390 Uruoca	1	0	0	0	1
231400 Várzea Alegre	1	0	0	0	1
231410 Viçosa do Ceará	4	0	0	0	4
240020 Açu	2	0	0	0	2
240080 Angicos	2	0	0	0	2
240100 Apodi	1	0	0	0	1
240150 Barcelona	1	0	0	0	1
240200 Caicó	6	0	0	0	6
240310 Currais Novos	2	0	0	0	2

240560 Jardim de Piranhas	1	0	0	0	1
240580 João Câmara	1	0	0	0	1
240710 Macaíba	4	0	0	0	4
240800 Mossoró	1	0	0	0	1
240810 Natal	28	0	0	0	28
240850 Ouro Branco	3	0	0	0	3
240325 Parnamirim	7	0	0	0	7
241120 Santa Cruz	1	0	0	0	1
241200 São Gonçalo do Amarante	9	0	0	0	9
241220 São José de Mipibu	2	0	0	0	2
241240 São José do Seridó	1	0	0	0	1
241300 São Vicente	1	0	0	0	1
241340 Serra Negra do Norte	1	0	0	1	2
241400 Tangará	1	0	0	0	1
240000 Município ignorado - RN	1	0	0	0	1
250030 Alagoa Grande	1	0	0	0	1
250180 Bayeux	1	0	0	0	1
250250 Boqueirão	1	0	0	0	1
250370 Cajazeiras	2	0	0	0	2
250375 Cajazeirinhas	1	0	0	0	1
250400 Campina Grande	7	1	0	0	8
250430 Catolé do Rocha	6	0	0	0	6
250460 Conde	1	0	0	0	1
250750 João Pessoa	7	1	0	0	8
250905 Marcação	1	0	0	0	1
251010 Nova Floresta	1	0	0	0	1
251080 Patos	7	0	0	3	10
251230 Princesa Isabel	1	0	0	0	1
251370 Santa Rita	1	0	0	0	1
251390 São Bento	1	0	0	0	1
251450 São José de Piranhas	1	0	0	0	1
251540 Seridó	1	0	0	0	1
251610 Soledade	2	0	0	0	2
251650 Taperoá	1	0	0	0	1
260005 Abreu e Lima	2	0	0	0	2
260010 Afogados da Ingazeira	1	0	0	0	1
260110 Araripina	2	0	0	0	2
260200 Bodocó	1	0	0	0	1
260290 Cabo de Santo Agostinho	2	2	0	0	4
260300 Cabrobó	1	0	0	0	1
260340 Calumbi	1	0	0	0	1
260345 Camaragibe	1	0	0	0	1
260400 Carpina	2	0	0	0	2
260410 Caruaru	2	0	0	0	2
260430 Cedro	2	0	0	0	2
260570 Floresta	1	0	0	0	1
260640 Gravatá	2	0	0	0	2
260720 Ipojuca	1	0	0	0	1
260790 Jaboatão dos Guararapes	18	0	0	0	18
260840 Jurema	1	0	0	0	1

260850 Lagoa do Itaenga	1	0	0	0	1
260875 Lagoa Grande	0	1	0	0	1
260940 Moreno	1	0	0	0	1
260950 Nazaré da Mata	1	0	0	0	1
260960 Olinda	6	1	0	0	7
260990 Ouricuri	2	0	0	0	2
261000 Palmares	1	0	0	0	1
261060 Paudalho	1	0	0	0	1
261070 Paulista	5	0	0	0	5
261110 Petrolina	3	0	0	0	3
261140 Primavera	1	0	0	0	1
261160 Recife	63	2	0	0	65
261250 Santa Cruz do Capibaribe	1	0	2	0	3
261350 São José do Belmonte	1	0	0	0	1
261360 São José do Egito	1	0	0	0	1
261410 Sertânia	1	0	0	0	1
261450 Surubim	1	0	0	0	1
261580 Tupanatinga	1	0	0	0	1
261640 Vitória de Santo Antão	1	0	0	0	1
270010 Água Branca	1	0	0	0	1
270020 Anadia	1	0	0	0	1
270030 Arapiraca	6	2	0	0	8
270050 Barra de Santo Antônio	1	0	0	0	1
270210 Colônia Leopoldina	0	1	0	0	1
270230 Coruripe	1	0	0	0	1
270235 Craíbas	1	0	0	0	1
270240 Delmiro Gouveia	8	1	0	0	9
270430 Maceió	17	0	0	0	17
270460 Maravilha	1	0	0	0	1
270470 Marechal Deodoro	2	0	0	0	2
270570 Olho d'Água das Flores	1	0	0	0	1
270860 São Miguel dos Campos	1	0	0	0	1
280020 Aquidabã	1	0	0	0	1
280030 Aracaju	11	1	0	0	12
280060 Barra dos Coqueiros	1	0	0	0	1
280067 Boquim	1	0	0	0	1
280120 Canindé de São Francisco	4	0	0	0	4
280150 Carmópolis	1	0	0	0	1
280210 Estância	2	0	0	0	2
280260 Gracho Cardoso	1	0	0	0	1
280380 Malhada dos Bois	1	0	0	0	1
280390 Malhador	1	0	0	0	1
280450 Nossa Senhora da Glória	1	0	0	0	1
280480 Nossa Senhora do Socorro	3	0	0	0	3
280530 Pirambu	1	0	0	0	1
280540 Poço Redondo	3	0	0	0	3
280560 Porto da Folha	1	0	0	0	1
280590 Riachuelo	1	0	0	0	1
280700 São Miguel do Aleixo	1	0	0	0	1
290070 Alagoinhas	2	0	0	0	2

290225 Arataca	1	0	0	0	1
290320 Barreiras	0	0	0	1	1
290327 Barrocas	1	1	0	0	2
290340 Belmonte	1	0	0	0	1
290520 Caetité	1	0	0	0	1
290570 Camaçari	2	0	0	0	2
290630 Canavieiras	1	0	0	0	1
290650 Candeias	3	0	0	0	3
290750 Catu	1	0	0	0	1
290790 Cipó	1	0	0	0	1
290800 Coaraci	1	0	0	0	1
290810 Cocos	1	0	0	0	1
290820 Conceição da Feira	1	0	0	0	1
290930 Correntina	2	0	0	0	2
291010 Dom Basílio	0	1	0	0	1
291050 Entre Rios	2	0	0	0	2
291072 Eunápolis	3	0	0	0	3
291080 Feira de Santana	3	0	0	0	3
291160 Governador Mangabeira	1	0	0	0	1
291185 Heliópolis	1	0	0	0	1
291190 Iaçu	4	0	0	0	4
291320 Ibotirama	1	0	0	0	1
291360 Ilhéus	3	0	0	0	3
291465 Itabela	3	0	0	0	3
291470 Itaberaba	1	0	0	0	1
291480 Itabuna	3	1	0	0	4
291530 Itagimirim	1	0	0	0	1
291535 Itaguaçu da Bahia	1	0	0	0	1
291560 Itamaraju	2	0	0	0	2
291630 Itapebi	1	0	0	0	1
291800 Jequié	1	0	0	0	1
291840 Juazeiro	1	1	0	0	2
291955 Luís Eduardo Magalhães	1	0	0	0	1
292090 Mascote	2	0	0	0	2
292100 Mata de São João	1	0	0	0	1
292200 Mucuri	1	0	0	0	1
292230 Muritiba	4	0	0	0	4
292300 Nova Viçosa	0	1	0	0	1
292400 Paulo Afonso	7	0	0	0	7
292520 Pojuca	3	0	0	0	3
292530 Porto Seguro	4	0	0	0	4
292600 Remanso	3	0	0	0	3
292740 Salvador	40	2	0	0	42
292840 Santa Rita de Cássia	1	0	0	0	1
292950 São Sebastião do Passé	1	0	0	0	1
293010 Senhor do Bonfim	1	0	0	0	1
293015 Serra do Ramalho	1	0	0	0	1
293050 Serrinha	1	0	0	0	1
293070 Simões Filho	5	0	0	0	5
293077 Sobradinho	2	0	0	0	2

293135 Teixeira de Freitas	2	0	0	0	2
293150 Teofilândia	1	0	0	1	2
293190 Tucano	1	0	0	0	1
310050 Açucena	0	1	0	0	1
310110 Aimorés	0	4	0	0	4
310150 Além Paraíba	0	1	0	0	1
310160 Alfenas	0	1	0	0	1
310170 Almenara	0	1	0	0	1
310350 Araguari	0	22	0	0	22
310400 Araxá	0	5	0	0	5
310420 Arcos	0	2	0	0	2
310450 Arinos	0	10	0	0	10
310490 Baependi	0	2	0	0	2
310510 Bambuí	0	1	0	0	1
310540 Barão de Cocais	0	2	0	0	2
310560 Barbacena	0	7	0	0	7
310570 Barra Longa	0	1	0	0	1
310620 Belo Horizonte	0	133	0	0	133
310670 Betim	0	22	0	0	22
310690 Bicas	0	2	0	0	2
310740 Bom Despacho	0	3	0	0	3
310860 Brasília de Minas	0	1	0	0	1
310890 Brasópolis	0	1	0	0	1
310900 Brumadinho	0	5	0	0	5
310930 Buritis	0	1	0	0	1
310980 Cachoeira Dourada	0	1	0	0	1
311000 Caeté	0	1	0	0	1
311050 Camanducaia	0	2	0	0	2
311090 Campanha	0	1	0	0	1
311140 Campo Florido	0	3	0	0	3
311160 Campos Gerais	0	1	0	0	1
311260 Capinópolis	0	1	0	0	1
311320 Carandaí	0	1	0	0	1
311535 Catas Altas	0	1	0	0	1
311570 Central de Minas	0	2	0	0	2
311730 Conceição das Alagoas	0	1	0	0	1
311800 Congonhas	0	1	0	0	1
311840 Conselheiro Pena	0	1	0	0	1
311860 Contagem	0	27	0	0	27
311910 Corinto	0	1	0	0	1
311930 Coromandel	0	4	0	0	4
311940 Coronel Fabriciano	0	4	0	0	4
312090 Curvelo	0	2	0	0	2
312160 Diamantina	0	1	0	0	1
312230 Divinópolis	0	14	0	0	14
312240 Divisa Nova	0	1	0	0	1
312390 Entre Rios de Minas	0	1	0	0	1
312410 Esmeraldas	0	3	0	0	3
312420 Espera Feliz	0	1	0	0	1
312480 Estrela do Sul	0	2	0	0	2

312595 Fervedouro	0	1	0	0	1
312610 Formiga	0	5	0	0	5
312710 Frutal	0	12	0	0	12
312730 Galiléia	0	1	0	0	1
312737 Goiabeira	0	1	0	0	1
312750 Gonzaga	0	1	0	0	1
312770 Governador Valadares	0	14	0	0	14
312780 Grão Mogol	0	2	0	0	2
312820 Guaraciaba	0	1	0	0	1
312830 Guaranésia	0	2	0	0	2
312870 Guaxupé	0	1	0	0	1
312890 Guimarânia	0	4	0	0	4
312930 Iapu	0	1	0	0	1
312980 Ibirité	0	8	0	0	8
313010 Igarapé	0	2	0	0	2
313115 Ipaba	0	1	0	0	1
313120 Ipanema	0	1	0	0	1
313130 Ipatinga	0	8	0	0	8
313170 Itabira	0	10	0	0	10
313180 Itabirinha	0	4	0	0	4
313190 Itabirito	0	3	0	0	3
313210 Itacarambi	0	1	0	0	1
313240 Itajubá	0	3	0	0	3
313300 Itamonte	0	3	0	0	3
313310 Itanhandu	0	1	0	0	1
313320 Itanhomi	0	2	0	0	2
313370 Itatiaiuçu	0	1	0	0	1
313375 Itaú de Minas	0	3	0	0	3
313380 Itaúna	0	2	0	0	2
313420 Ituiutaba	0	5	0	0	5
313480 Jacuí	0	2	0	0	2
313505 Jaíba	0	1	0	0	1
313510 Janaúba	0	2	0	0	2
313655 José Raydan	0	2	0	0	2
313665 Juatuba	0	1	0	0	1
313670 Juiz de Fora	0	15	0	0	15
313690 Juruaia	0	1	0	0	1
313720 Lagoa da Prata	0	6	0	0	6
313740 Lagoa Dourada	0	1	0	0	1
313750 Lagoa Formosa	0	1	0	0	1
313760 Lagoa Santa	0	4	0	0	4
313820 Lavras	0	2	0	0	2
313840 Leopoldina	0	1	0	0	1
313880 Luz	0	2	0	0	2
313900 Machado	0	1	0	0	1
313960 Mantena	0	7	0	0	7
313970 Maravilhas	0	2	0	0	2
314000 Mariana	0	5	0	0	5
314050 Martinho Campos	0	1	0	0	1
314170 Mesquita	0	1	0	0	1

314240 Moema	0	1	0	0	1
314310 Monte Carmelo	0	4	0	0	4
314330 Montes Claros	0	1	0	0	1
314390 Muriaé	0	3	0	0	3
314460 Nepomuceno	0	1	0	0	1
314470 Nova Era	0	2	0	0	2
314480 Nova Lima	0	6	0	0	6
314500 Nova Ponte	0	1	0	0	1
314537 Novorizonte	0	1	0	0	1
314560 Oliveira	0	2	0	0	2
314590 Ouro Branco	0	2	0	0	2
314610 Ouro Preto	0	2	0	0	2
314630 Padre Paraíso	0	1	0	0	1
314650 Pains	0	3	0	0	3
314710 Pará de Minas	0	3	0	0	3
314700 Paracatu	0	8	0	0	8
314790 Passos	0	2	0	0	2
314800 Patos de Minas	0	6	0	0	6
314810 Patrocínio	0	10	0	0	10
315150 Piumhi	0	1	0	0	1
315210 Ponte Nova	0	2	0	0	2
315220 Porteirinha	0	2	0	0	2
315250 Pouso Alegre	0	5	0	0	5
315290 Pratápolis	0	1	0	0	1
315300 Pratinha	0	1	0	0	1
315340 Presidente Olegário	0	1	0	0	1
315390 Raposos	0	0	1	0	1
315400 Raul Soares	0	1	0	0	1
315430 Resplendor	0	1	0	0	1
315460 Ribeirão das Neves	0	9	0	0	9
315570 Rio Piracicaba	0	2	0	0	2
315580 Rio Pomba	0	1	0	0	1
315590 Rio Preto	0	1	0	0	1
315670 Sabará	0	6	0	0	6
315690 Sacramento	0	1	0	0	1
315720 Santa Bárbara	0	1	0	0	1
315780 Santa Luzia	0	10	0	0	10
315820 Santa Maria do Suaçuí	0	7	0	0	7
315980 Santa Vitória	0	1	0	0	1
316040 Santo Antônio do Monte	0	4	0	0	4
316090 São Brás do Suaçuí	0	1	0	0	1
316095 São Domingos das Dores	0	1	0	0	1
316110 São Francisco	0	1	0	0	1
316130 São Francisco de Sales	0	3	0	0	3
316190 São Gonçalo do Rio Abaixo	0	1	0	0	1
316210 São Gotardo	0	3	0	0	3
316250 São João del Rei	0	4	0	0	4
316257 São João do Manteninha	0	1	0	0	1
316290 São João Nepomuceno	0	1	0	0	1
316292 São Joaquim de Bicas	0	3	0	0	3

316294 São José da Barra	0	1	0	0	1
316350 São José do Jacuri	0	1	0	0	1
316410 São Pedro do Suaçuí	0	1	0	0	1
316553 Sarzedo	0	3	0	0	3
316670 Serra dos Aimorés	0	2	0	0	2
316720 Sete Lagoas	0	2	0	0	2
316800 Taiobeiras	0	1	0	0	1
316860 Teófilo Otoni	0	1	0	0	1
316870 Timóteo	0	1	0	0	1
316930 Três Corações	0	1	0	0	1
316935 Três Marias	0	2	0	0	2
316990 Ubá	0	1	0	0	1
317010 Uberaba	0	14	0	0	14
317020 Uberlândia	0	44	0	1	45
317040 Unaí	0	8	0	0	8
317065 Vargem Grande do Rio Pardo	0	1	0	0	1
317070 Varginha	0	1	0	0	1
317100 Vazante	0	1	0	0	1
317110 Veríssimo	0	2	0	0	2
317120 Vespasiano	0	3	0	0	3
317130 Viçosa	0	3	0	0	3
320010 Afonso Cláudio	0	1	0	0	1
320016 Água Doce do Norte	0	1	0	0	1
320013 Águia Branca	0	2	0	0	2
320040 Anchieta	0	2	0	0	2
320060 Aracruz	1	2	0	0	3
320080 Baixo Guandu	0	8	0	0	8
320090 Barra de São Francisco	0	11	0	0	11
320100 Boa Esperança	0	2	0	0	2
320120 Cachoeiro de Itapemirim	0	1	0	0	1
320130 Cariacica	0	21	0	2	23
320140 Castelo	0	1	0	0	1
320150 Colatina	0	12	0	1	13
320170 Conceição do Castelo	0	2	0	0	2
320190 Domingos Martins	0	1	0	0	1
320210 Ecoporanga	0	2	0	0	2
320220 Fundão	0	2	0	0	2
320225 Governador Lindenberg	0	2	0	0	2
320240 Guarapari	0	7	0	0	7
320250 Ibiraçu	0	1	0	0	1
320260 Iconha	0	2	0	0	2
320270 Itaguaçu	0	3	0	0	3
320280 Itapemirim	0	1	0	0	1
320305 Jaguaré	0	3	0	0	3
320313 João Neiva	0	2	0	0	2
320320 Linhares	0	30	2	1	33
320332 Marataízes	0	1	0	0	1
320334 Marechal Floriano	0	3	0	0	3
320350 Montanha	0	2	0	0	2
320390 Nova Venécia	0	6	0	0	6

320400 Pancas	0	2	0	0	2
320405 Pedro Canário	0	1	0	0	1
320425 Ponto Belo	0	3	0	0	3
320440 Rio Novo do Sul	0	1	0	0	1
320455 Santa Maria de Jetibá	0	2	0	0	2
320460 Santa Teresa	0	5	0	0	5
320470 São Gabriel da Palha	0	3	0	0	3
320490 São Mateus	0	9	0	0	9
320495 São Roque do Canaã	0	4	0	0	4
320500 Serra	0	23	0	0	23
320506 Venda Nova do Imigrante	0	2	0	0	2
320510 Viana	0	2	0	0	2
320515 Vila Pavão	0	4	0	0	4
320517 Vila Valério	0	4	0	0	4
320520 Vila Velha	0	25	0	0	25
320530 Vitória	0	27	0	0	27
330010 Angra dos Reis	0	2	0	0	2
330020 Araruama	0	1	0	0	1
330030 Barra do Piraí	0	3	0	0	3
330045 Belford Roxo	0	2	0	0	2
330060 Bom Jesus do Itabapoana	0	1	0	0	1
330070 Cabo Frio	0	3	0	0	3
330080 Cachoeiras de Macacu	0	2	0	0	2
330100 Campos dos Goytacazes	0	6	0	0	6
330140 Conceição de Macabu	0	1	0	0	1
330150 Cordeiro	0	1	0	0	1
330170 Duque de Caxias	0	16	0	0	16
330190 Itaboraí	0	1	0	0	1
330200 Itaguaí	0	3	0	0	3
330220 Itaperuna	0	1	0	0	1
330225 Itatiaia	0	1	0	0	1
330240 Macaé	0	24	0	0	24
330250 Magé	0	2	0	0	2
330260 Mangaratiba	0	1	0	0	1
330270 Maricá	0	3	0	0	3
330285 Mesquita	0	2	0	0	2
330320 Nilópolis	0	1	0	0	1
330330 Niterói	0	20	0	0	20
330340 Nova Friburgo	0	2	0	0	2
330350 Nova Iguaçu	0	14	0	0	14
330360 Paracambi	0	3	0	0	3
330390 Petrópolis	0	2	0	0	2
330410 Porciúncula	0	2	0	0	2
330414 Queimados	0	2	0	0	2
330420 Resende	0	5	0	0	5
330430 Rio Bonito	0	1	0	0	1
330440 Rio Claro	0	1	0	0	1
330452 Rio das Ostras	0	8	0	0	8
330455 Rio de Janeiro	0	386	0	1	387
330490 São Gonçalo	0	15	0	0	15

330500 São João da Barra	0	1	0	0	1
330510 São João de Meriti	0	3	0	0	3
330540 Sapucaia	0	1	0	0	1
330580 Teresópolis	0	4	0	0	4
330610 Valença	0	4	0	0	4
330630 Volta Redonda	0	1	0	0	1
350010 Adamantina	0	1	0	0	1
350040 Águas da Prata	0	1	0	0	1
350050 Águas de Lindóia	0	1	0	0	1
350070 Agudos	0	1	0	0	1
350160 Americana	0	3	0	0	3
350170 Américo Brasiliense	0	1	0	0	1
350210 Andradina	0	2	0	0	2
350260 Aparecida d'Oeste	0	1	0	0	1
350270 Apiaí	0	2	0	0	2
350275 Araçariguama	0	1	0	0	1
350280 Araçatuba	0	3	1	0	4
350320 Araraquara	0	2	0	0	2
350330 Araras	0	2	0	0	2
350380 Artur Nogueira	0	2	0	0	2
350390 Arujá	0	4	0	0	4
350400 Assis	0	2	0	0	2
350410 Atibaia	0	3	0	0	3
350460 Bady Bassitt	0	2	0	0	2
350480 Bálsamo	0	1	0	0	1
350550 Barretos	0	4	0	0	4
350560 Barrinha	0	2	0	0	2
350570 Barueri	0	10	0	0	10
350580 Bastos	0	1	0	0	1
350590 Batatais	0	6	0	0	6
350600 Bauru	0	9	0	0	9
350610 Bebedouro	0	3	0	0	3
350635 Bertioga	0	3	0	1	4
350700 Boituva	0	1	0	0	1
350710 Bom Jesus dos Perdões	0	2	0	0	2
350730 Boracéia	0	1	0	0	1
350750 Botucatu	0	3	0	0	3
350760 Bragança Paulista	0	5	0	0	5
350780 Brodowski	0	1	0	0	1
350790 Brotas	0	1	0	0	1
350840 Cabreúva	0	1	0	0	1
350850 Caçapava	0	6	0	0	6
350900 Caieiras	0	3	0	0	3
350920 Cajamar	0	5	0	0	5
350950 Campinas	0	33	0	1	34
350960 Campo Limpo Paulista	0	1	0	0	1
350970 Campos do Jordão	0	1	0	0	1
351000 Cândido Mota	0	7	0	0	7
351020 Capão Bonito	0	1	0	0	1
351060 Carapicuíba	0	8	0	0	8

351070 Cardoso	0	1	0	0	1
351100 Castilho	0	2	0	0	2
351170 Charqueada	0	1	0	0	1
351190 Clementina	0	1	0	0	1
351240 Cordeirópolis	0	1	0	0	1
351300 Cotia	0	3	0	0	3
351350 Cubatão	0	4	0	0	4
351380 Diadema	0	4	0	0	4
351410 Dois Córregos	0	1	0	0	1
351440 Dracena	0	1	0	0	1
351470 Echaporã	0	1	0	0	1
351490 Elias Fausto	0	1	0	0	1
351500 Embu	0	5	0	0	5
351570 Ferraz de Vasconcelos	0	1	0	0	1
351610 Florínia	0	1	0	0	1
351620 Franca	0	7	0	0	7
351630 Francisco Morato	0	4	0	0	4
351640 Franco da Rocha	0	2	0	0	2
351670 Garça	0	4	0	0	4
351710 Glicério	0	1	0	0	1
351740 Guaíra	0	2	0	0	2
351750 Guapiaçu	0	2	0	0	2
351780 Guaraçaí	0	1	0	0	1
351820 Guararapes	0	1	0	0	1
351830 Guararema	0	1	0	0	1
351840 Guaratinguetá	0	3	0	0	3
351870 Guarujá	0	5	0	0	5
351880 Guarulhos	0	35	0	0	35
351890 Guzolândia	0	1	0	0	1
351905 Holambra	0	2	0	0	2
351907 Hortolândia	0	6	1	0	7
352044 Ilha Solteira	0	1	0	1	2
352040 Ilhabela	0	2	0	0	2
352050 Indaiatuba	0	4	0	0	4
352100 Iperó	0	1	0	0	1
352140 Iracemápolis	0	1	0	0	1
352170 Itaberá	0	1	0	0	1
352220 Itapecerica da Serra	0	5	0	0	5
352230 Itapetininga	0	2	0	0	2
352240 Itapeva	0	2	0	0	2
352250 Itapevi	0	2	0	0	2
352310 Itaquaquecetuba	0	3	0	0	3
352330 Itariri	0	1	0	0	1
352340 Itatiba	0	1	0	0	1
352390 Itu	0	5	0	0	5
352410 Ituverava	0	1	0	0	1
352430 Jaboticabal	0	1	0	0	1
352440 Jacareí	0	6	0	0	6
352460 Jacupiranga	1	0	0	0	1
352470 Jaguariúna	0	7	0	0	7

352480	Jales	0	2	0	0	2
352500	Jandira	0	4	0	0	4
352510	Jardinópolis	0	1	0	0	1
352520	Jarinu	0	1	0	0	1
352530	Jaú	0	1	0	0	1
352590	Jundiaí	0	14	0	0	14
352600	Junqueirópolis	0	2	0	0	2
352620	Juquitiba	0	2	0	0	2
352640	Laranjal Paulista	0	1	0	0	1
352680	Lençóis Paulista	0	4	0	0	4
352690	Limeira	0	16	0	0	16
352710	Lins	0	2	0	0	2
352770	Luiziânia	0	1	0	1	2
352820	Macedônia	0	1	0	0	1
352850	Mairiporã	0	1	0	0	1
352890	Mariápolis	0	1	0	0	1
352900	Marília	0	9	1	0	10
352930	Matão	0	1	0	0	1
352940	Mauá	0	7	0	0	7
353010	Mirandópolis	0	1	0	0	1
353020	Mirante do Paranapanema	0	1	0	0	1
353030	Mirassol	0	1	0	0	1
353050	Mococa	0	2	0	0	2
353060	Mogi das Cruzes	0	5	0	0	5
353070	Mogi Guaçu	0	6	0	0	6
353080	Moji Mirim	0	2	0	0	2
353110	Mongaguá	0	1	0	0	1
353120	Monte Alegre do Sul	0	1	0	0	1
353150	Monte Azul Paulista	0	2	0	0	2
353180	Monte Mor	0	1	0	0	1
353250	Neves Paulista	0	1	0	0	1
353260	Nhandeara	0	2	0	0	2
353270	Nipoã	0	1	0	0	1
353300	Nova Granada	0	3	0	0	3
353380	Óleo	0	1	0	0	1
353390	Olímpia	0	3	0	0	3
353400	Onda Verde	0	1	0	0	1
353440	Osasco	0	20	0	0	20
353460	Osvaldo Cruz	0	1	0	0	1
353470	Ourinhos	0	2	1	0	3
353500	Palestina	0	2	0	0	2
353530	Palmital	0	1	0	0	1
353550	Paraguaçu Paulista	0	3	0	0	3
353600	Parapuã	0	1	0	0	1
353610	Pardinho	0	1	0	0	1
353625	Parisi	0	1	0	0	1
353650	Paulínia	0	4	0	0	4
353730	Penápolis	0	4	0	0	4
353760	Peruíbe	0	1	0	0	1
353770	Piacatu	0	1	0	0	1

353780 Piedade	0	4	0	0	4
353800 Pindamonhangaba	0	2	0	0	2
353810 Pindorama	0	1	0	0	1
353860 Piracaia	0	2	0	0	2
353870 Piracicaba	0	7	0	0	7
353890 Pirajuí	0	1	0	0	1
353920 Pirapozinho	0	5	0	0	5
353930 Pirassununga	0	2	0	0	2
353980 Poá	0	1	0	0	1
354000 Pompéia	0	1	0	0	1
354020 Pontal	0	1	0	0	1
354025 Pontalinda	0	2	0	0	2
354060 Porto Feliz	0	1	0	0	1
354070 Porto Ferreira	0	1	0	0	1
354075 Potim	0	2	0	0	2
354100 Praia Grande	0	2	0	0	2
354105 Pratânia	0	1	0	0	1
354120 Presidente Bernardes	0	1	0	0	1
354130 Presidente Epitácio	0	1	0	0	1
354140 Presidente Prudente	0	18	1	0	19
354150 Presidente Venceslau	0	2	0	0	2
354170 Quatá	0	2	0	0	2
354240 Regente Feijó	0	1	0	0	1
354260 Registro	0	2	0	0	2
354325 Ribeirão Grande	0	1	0	0	1
354330 Ribeirão Pires	0	2	0	0	2
354340 Ribeirão Preto	0	25	0	0	25
354390 Rio Claro	0	2	0	0	2
354400 Rio das Pedras	0	1	0	0	1
354410 Rio Grande da Serra	0	1	0	0	1
354425 Rosana	0	5	0	0	5
354520 Salto	0	1	0	0	1
354530 Salto de Pirapora	0	1	0	0	1
354580 Santa Bárbara d'Oeste	0	5	0	0	5
354660 Santa Fé do Sul	0	2	0	0	2
354680 Santa Isabel	0	1	0	0	1
354730 Santana de Parnaíba	0	7	0	0	7
354770 Santo Anastácio	0	2	0	0	2
354780 Santo André	0	19	0	0	19
354800 Santo Antônio de Posse	0	2	0	0	2
354850 Santos	0	11	0	0	11
354870 São Bernardo do Campo	1	23	0	0	24
354880 São Caetano do Sul	0	2	0	0	2
354890 São Carlos	0	5	0	0	5
354910 São João da Boa Vista	0	4	0	0	4
354980 São José do Rio Preto	0	28	0	0	28
354990 São José dos Campos	0	21	0	0	21
354995 São Lourenço da Serra	0	2	0	0	2
355010 São Manuel	0	2	0	0	2
355030 São Paulo	0	389	0	3	392

355050 São Pedro do Turvo	0	1	0	0	1
355060 São Roque	0	1	0	0	1
355070 São Sebastião	0	4	0	0	4
355100 São Vicente	0	12	0	0	12
355150 Serrana	0	1	0	0	1
355170 Sertãozinho	0	2	0	0	2
355220 Sorocaba	0	12	0	0	12
355240 Sumaré	0	7	0	0	7
355250 Suzano	0	6	0	0	6
355280 Taboão da Serra	0	6	0	0	6
355290 Taciba	0	1	0	0	1
355360 Tapiratiba	0	1	0	0	1
355400 Tatuí	0	1	0	0	1
355410 Taubaté	0	1	0	0	1
355420 Tejupá	0	1	0	0	1
355430 Teodoro Sampaio	0	1	0	0	1
355480 Tremembé	0	2	0	0	2
355500 Tupã	0	2	0	0	2
355540 Ubatuba	0	2	0	0	2
355580 Urânia	0	5	0	0	5
355620 Valinhos	0	2	0	0	2
355645 Vargem Grande Paulista	0	3	0	0	3
355650 Várzea Paulista	0	2	0	0	2
355670 Vinhedo	0	6	0	0	6
355700 Votorantim	0	2	0	0	2
355710 Votuporanga	0	5	0	0	5
410040 Almirante Tamandaré	0	0	2	0	2
410050 Altônia	0	0	2	0	2
410100 Ampére	0	0	2	0	2
410110 Andirá	0	0	1	0	1
410120 Antonina	0	0	3	0	3
410140 Apucarana	0	0	2	0	2
410150 Arapongas	0	0	3	0	3
410180 Araucária	0	0	4	1	5
410190 Assaí	0	0	1	0	1
410200 Assis Chateaubriand	0	0	5	0	5
410300 Boa Esperança	0	0	1	0	1
410305 Boa Vista da Aparecida	0	1	2	0	3
410345 Cafelândia	0	1	3	0	4
410350 Califórnia	0	0	2	0	2
410370 Cambé	0	0	1	0	1
410400 Campina Grande do Sul	0	0	2	0	2
410405 Campo Bonito	0	0	1	0	1
410420 Campo Largo	0	0	8	0	8
410430 Campo Mourão	0	0	1	0	1
410440 Cândido de Abreu	0	0	1	0	1
410450 Capanema	0	0	2	0	2
410460 Capitão Leônidas Marques	0	0	1	0	1
410480 Cascavel	0	0	33	0	33
410510 Centenário do Sul	0	0	2	0	2

410530 Céu Azul	0	0	5	0	5
410540 Chopinzinho	0	0	3	0	3
410580 Colombo	0	0	4	0	4
410630 Corbélia	0	0	10	0	10
410640 Cornélio Procópio	0	0	1	0	1
410650 Coronel Vivida	0	0	1	0	1
410660 Cruzeiro do Oeste	0	0	2	0	2
410690 Curitiba	0	1	74	0	75
410715 Diamante D'Oeste	0	0	1	0	1
410710 Diamante do Norte	0	0	1	0	1
410725 Douradina	0	0	2	0	2
410753 Entre Rios do Oeste	0	0	1	0	1
410765 Fazenda Rio Grande	0	0	3	0	3
410790 Floresta	0	0	1	0	1
410820 Formosa do Oeste	0	0	2	0	2
410830 Foz do Iguaçu	0	0	54	0	54
410832 Francisco Alves	0	0	1	0	1
410840 Francisco Beltrão	0	1	5	0	6
410860 Goioerê	0	0	1	0	1
410880 Guaíra	0	0	3	0	3
410890 Guairaçá	0	0	1	0	1
410930 Guaraniaçu	0	0	4	0	4
410940 Guarapuava	0	0	3	0	3
410965 Honório Serpa	0	0	1	0	1
410970 Ibaiti	0	0	2	0	2
410980 Ibiporã	0	0	1	0	1
410990 Icaraíma	0	0	1	0	1
411070 Irati	0	0	2	0	2
411100 Itambaracá	0	0	1	0	1
411130 Itaúna do Sul	0	0	1	0	1
411160 Ivatuba	0	0	1	0	1
411180 Jacarezinho	0	0	1	0	1
411260 Jardim Olinda	0	0	1	0	1
411270 Jataizinho	0	0	1	0	1
411280 Joaquim Távora	0	0	1	0	1
411330 Laranjeiras do Sul	0	0	3	0	3
411370 Londrina	0	1	17	0	18
411390 Mallet	0	0	1	0	1
411400 Mamborê	0	0	1	0	1
411410 Mandaguaçu	0	1	0	0	1
411460 Marechal Cândido Rondon	0	0	6	0	6
411480 Marialva	0	0	4	0	4
411490 Marilândia do Sul	0	0	1	0	1
411520 Maringá	0	2	35	0	37
411575 Mauá da Serra	0	0	1	0	1
411580 Medianeira	0	0	4	0	4
411585 Mercedes	0	0	2	0	2
411590 Mirador	0	0	1	0	1
411605 Missal	0	0	2	0	2
411670 Nova Aurora	0	0	1	0	1

411690 Nova Esperança	0	0	1	0	1
411710 Nova Londrina	0	0	2	0	2
411725 Nova Prata do Iguaçu	0	0	3	0	3
411730 Ortigueira	0	0	1	0	1
411740 Ourizona	0	0	1	0	1
411820 Paranaguá	0	0	1	0	1
411840 Paranavaí	0	1	4	0	5
411850 Pato Branco	0	0	4	0	4
411910 Piên	0	0	1	0	1
411915 Pinhais	0	0	1	0	1
411940 Piraí do Sul	0	0	1	0	1
411950 Piraquara	0	0	4	0	4
411990 Ponta Grossa	0	1	7	0	8
412000 Porecatu	0	0	1	0	1
412060 Prudentópolis	0	0	4	0	4
412090 Quedas do Iguaçu	0	0	5	1	6
412100 Querência do Norte	0	0	3	0	3
412140 Realeza	0	0	4	0	4
412215 Rio Bonito do Iguaçu	0	0	1	0	1
412230 Rio Negro	0	0	1	0	1
412280 Salgado Filho	0	0	1	0	1
412300 Salto do Lontra	0	0	1	0	1
412350 Santa Helena	0	0	21	0	21
412380 Santa Izabel do Oeste	0	0	3	0	3
412405 Santa Terezinha de Itaipu	0	0	7	0	7
412410 Santo Antônio da Platina	0	0	2	0	2
412440 Santo Antônio do Sudoeste	0	0	1	0	1
412520 São Jorge d'Oeste	0	0	1	0	1
412550 São José dos Pinhais	0	1	15	0	16
412570 São Miguel do Iguaçu	0	0	13	0	13
412575 São Pedro do Iguaçu	0	0	1	0	1
412625 Sarandi	0	0	2	0	2
412710 Telêmaco Borba	0	0	1	0	1
412730 Terra Rica	0	0	1	0	1
412740 Terra Roxa	0	0	1	0	1
412770 Toledo	0	0	11	0	11
412785 Três Barras do Paraná	0	0	3	0	3
412790 Tuneiras do Oeste	0	0	1	0	1
412800 Ubiratã	0	0	1	0	1
412810 Umuarama	0	0	4	0	4
412855 Vera Cruz do Oeste	0	0	1	0	1
412860 Verê	0	0	1	0	1
420010 Abelardo Luz	0	0	1	0	1
420030 Agronômica	0	0	1	0	1
420050 Águas de Chapecó	0	0	3	0	3
420055 Águas Frias	0	0	1	0	1
420200 Balneário Camboriú	0	0	3	0	3
421280 Balneário Piçarras	0	0	1	0	1
420208 Bandeirante	0	1	0	0	1
420210 Barra Velha	0	0	3	0	3

420230 Biguaçu	0	0	1	0	1
420240 Blumenau	0	0	9	0	9
420280 Braço do Norte	0	0	6	0	6
420290 Brusque	0	0	2	0	2
420300 Caçador	0	0	1	0	1
420310 Caibi	0	0	2	0	2
420320 Camboriú	0	0	4	0	4
420380 Canoinhas	1	0	2	0	3
420390 Capinzal	0	0	2	0	2
420400 Catanduvas	0	0	1	0	1
420415 Celso Ramos	0	0	1	0	1
420420 Chapecó	0	0	5	2	7
420430 Concórdia	0	1	11	0	12
420435 Cordilheira Alta	0	0	1	0	1
420440 Coronel Freitas	0	0	1	0	1
420460 Criciúma	0	0	2	0	2
420470 Cunha Porã	1	0	3	1	5
420540 Florianópolis	0	1	25	0	26
420545 Forquilhinha	0	0	1	0	1
420550 Fraiburgo	0	0	3	0	3
420570 Garopaba	0	0	1	0	1
420580 Garuva	0	0	1	0	1
420590 Gaspar	0	0	2	0	2
420610 Grão Pará	0	0	1	0	1
420640 Guaraciaba	0	0	1	0	1
420650 Guaramirim	0	0	3	0	3
420660 Guarujá do Sul	0	1	6	0	7
420700 Içara	0	0	1	0	1
420730 Imbituba	0	1	1	0	2
420740 Imbuia	0	0	1	0	1
420750 Indaial	0	0	1	0	1
420765 Iporã do Oeste	1	0	0	0	1
420800 Itá	0	0	1	0	1
420810 Itaiópolis	0	0	3	0	3
420820 Itajaí	0	0	7	0	7
420830 Itapema	0	0	2	0	2
420840 Itapiranga	0	0	1	0	1
420880 Jaguaruna	0	0	1	0	1
420890 Jaraguá do Sul	0	0	4	0	4
420900 Joaçaba	0	0	2	0	2
420910 Joinville	0	0	9	0	9
420915 José Boiteux	1	0	0	0	1
420930 Lages	0	0	1	0	1
421050 Maravilha	0	0	4	0	4
421100 Mondaí	1	0	1	0	2
421130 Navegantes	0	0	2	0	2
421170 Orleans	0	0	1	0	1
421190 Palhoça	0	0	2	0	2
421200 Palma Sola	0	0	1	0	1
421210 Palmitos	0	0	2	0	2

421220 Papanduva	0	0	2	0	2
421225 Passo de Torres	0	0	1	0	1
421320 Pomerode	0	0	1	0	1
421350 Porto Belo	0	0	1	0	1
421420 Quilombo	0	0	1	0	1
421440 Rio das Antas	0	0	1	0	1
421480 Rio do Sul	0	0	3	0	3
421500 Rio Negrinho	0	0	1	0	1
421580 São Bento do Sul	0	0	1	0	1
421575 São Bernardino	0	0	1	0	1
421620 São Francisco do Sul	0	0	1	0	1
421630 São João Batista	0	0	3	0	3
421660 São José	0	0	6	0	6
421690 São Lourenço do Oeste	0	0	4	0	4
421720 São Miguel do Oeste	0	0	6	1	7
421740 Schroeder	0	0	1	0	1
421760 Siderópolis	0	0	2	1	3
421820 Timbó	0	0	2	0	2
421830 Três Barras	0	0	1	0	1
421840 Treze de Maio	0	0	1	0	1
421870 Tubarão	0	1	4	0	5
421900 Urussanga	2	0	0	0	2
421917 Vargem Bonita	0	0	1	0	1
421970 Xaxim	0	0	3	0	3
430160 Bagé	0	0	1	0	1
430195 Barra Funda	0	0	1	0	1
430230 Bom Jesus	0	0	1	0	1
430300 Cachoeira do Sul	0	0	3	0	3
430440 Canela	0	0	1	0	1
430460 Canoas	0	0	4	0	4
430463 Capão da Canoa	0	0	1	0	1
430480 Carlos Barbosa	0	0	1	0	1
430510 Caxias do Sul	0	0	4	0	4
430545 Cidreira	0	0	1	0	1
430570 Condor	0	0	1	0	1
430610 Cruz Alta	0	0	6	0	6
430790 Farroupilha	0	1	2	0	3
430845 Fortaleza dos Valos	0	0	1	0	1
430860 Garibaldi	0	0	3	0	3
430920 Gravataí	0	0	1	0	1
430950 Guarani das Missões	0	0	1	0	1
431000 Ibirubá	0	0	0	1	1
431020 Ijuí	0	0	1	0	1
431115 Jóia	0	0	1	0	1
431140 Lajeado	0	0	1	0	1
431180 Marau	0	0	3	0	3
431230 Miraguaí	0	0	1	0	1
431240 Montenegro	0	0	3	0	3
431270 Nonoai	0	0	1	0	1
431290 Nova Bassano	0	0	1	0	1

431330 Nova Prata	0	0	1	0	1
431340 Novo Hamburgo	0	0	1	0	1
431350 Osório	0	0	1	0	1
431410 Passo Fundo	0	0	5	0	5
431430 Pejuçara	0	0	1	0	1
431440 Pelotas	0	0	1	0	1
431480 Portão	0	0	3	0	3
431490 Porto Alegre	0	0	16	0	16
431500 Porto Lucena	0	0	1	0	1
431630 Roque Gonzales	0	0	1	0	1
431710 Sant' Ana do Livramento	0	0	1	0	1
431690 Santa Maria	0	0	3	0	3
431720 Santa Rosa	0	0	2	0	2
431730 Santa Vitória do Palmar	0	0	1	0	1
431740 Santiago	0	0	1	0	1
431760 Santo Antônio da Patrulha	0	0	1	0	1
431775 Santo Antônio do Planalto	0	0	1	0	1
431780 Santo Augusto	0	0	1	0	1
431800 São Borja	0	0	1	0	1
431870 São Leopoldo	0	0	3	0	3
431900 São Marcos	0	0	1	0	1
431971 São Valentim do Sul	0	0	1	0	1
432010 Sarandi	0	0	1	0	1
432110 Tapes	0	0	1	0	1
432140 Tenente Portela	0	0	2	0	2
432150 Torres	0	0	1	0	1
432160 Tramandaí	0	0	1	0	1
432180 Três de Maio	0	0	1	0	1
432195 Trindade do Sul	0	0	1	0	1
432230 Tuparendi	0	0	2	0	2
432250 Vacaria	0	0	1	0	1
432260 Venâncio Aires	0	0	1	0	1
500060 Amambai	0	0	0	1	1
500070 Anastácio	0	0	0	3	3
500100 Aparecida do Taboado	0	0	0	3	3
500260 Camapuã	0	0	0	2	2
500270 Campo Grande	0	1	0	91	92
500295 Chapadão do Sul	0	0	0	2	2
500320 Corumbá	0	0	0	1	1
500325 Costa Rica	0	0	0	5	5
500330 Coxim	0	0	0	2	2
500350 Douradina	0	0	0	1	1
500370 Dourados	0	0	0	10	10
500375 Eldorado	0	0	0	1	1
500460 Itaquiraí	0	0	0	5	5
500500 Jardim	0	0	0	1	1
500515 Juti	0	0	0	1	1
500568 Mundo Novo	0	0	0	1	1
500570 Naviraí	0	0	0	2	2
500620 Nova Andradina	0	0	0	2	2

500720 Rio Brilhante	0	0	0	1	1
500769 São Gabriel do Oeste	0	0	0	6	6
500790 Sidrolândia	0	0	0	2	2
500793 Sonora	0	0	0	6	6
500795 Tacuru	0	0	0	2	2
500797 Taquarussu	0	0	0	2	2
500830 Três Lagoas	0	0	0	5	5
510025 Alta Floresta	1	0	0	1	2
510030 Alto Araguaia	0	0	0	1	1
510180 Barra do Garças	0	0	0	2	2
510325 Colniza	0	0	1	1	2
510330 Comodoro	0	0	1	0	1
510340 Cuiabá	0	1	1	1	3
510385 Gaúcha do Norte	0	1	0	0	1
510410 Guarantã do Norte	0	0	0	1	1
510558 Marcelândia	0	0	1	0	1
510629 Paranaíta	0	1	0	0	1
510740 São Pedro da Cipa	0	0	1	0	1
510790 Sinop	0	1	0	0	1
510792 Sorriso	0	0	0	1	1
510795 Tangará da Serra	0	1	0	1	2
510840 Várzea Grande	0	3	0	0	3
510000 Município ignorado - MT	0	1	0	0	1
520025 Águas Lindas de Goiás	0	0	0	3	3
520055 Alto Horizonte	0	0	0	1	1
520110 Anápolis	0	3	0	27	30
520130 Anicuns	0	0	0	1	1
520140 Aparecida de Goiânia	0	0	0	53	53
520150 Aporé	0	0	0	2	2
520215 Araguapaz	0	0	0	2	2
520320 Barro Alto	0	0	0	1	1
520350 Bom Jesus de Goiás	0	0	0	1	1
520380 Britânia	0	0	0	1	1
520393 Buriti de Goiás	0	0	0	1	1
520410 Cachoeira Alta	0	0	0	1	1
520425 Cachoeira Dourada	0	0	0	1	1
520440 Caiapônia	0	0	0	3	3
520450 Caldas Novas	0	0	0	8	8
520485 Campo Limpo de Goiás	0	0	0	1	1
520495 Campos Verdes	0	0	0	1	1
520545 Cezarina	0	0	0	1	1
520549 Cidade Ocidental	0	0	0	2	2
520551 Cocalzinho de Goiás	0	0	0	1	1
520570 Córrego do Ouro	0	0	0	1	1
520590 Corumbaíba	0	0	0	1	1
520620 Cristalina	0	0	0	2	2
520840 Goianápolis	0	0	0	1	1
520860 Goianésia	0	0	0	4	4
520870 Goiânia	1	3	0	205	209
520880 Goianira	0	0	0	4	4

520890 Goiás	0	0	0	4	4
520910 Goiatuba	0	0	0	3	3
520920 Guapó	0	0	0	1	1
520995 Indiara	0	0	0	1	1
521000 Inhumas	0	0	0	2	2
521020 Iporá	0	0	0	3	3
521120 Itapuranga	0	0	0	1	1
521150 Itumbiara	0	0	0	2	2
521180 Jaraguá	0	0	0	2	2
521190 Jataí	0	0	0	7	7
521220 Jussara	0	0	0	1	1
521250 Luziânia	0	0	0	12	12
521300 Maurilândia	0	0	0	1	1
521308 Minaçu	0	0	0	19	19
521310 Mineiros	0	0	0	4	4
521380 Morrinhos	0	0	0	2	2
521450 Nerópolis	0	0	0	1	1
521460 Niquelândia	0	0	0	4	4
521486 Nova Glória	0	0	0	1	1
521570 Palmeiras de Goiás	0	0	0	3	3
521720 Piranhas	0	0	0	1	1
521800 Porangatu	0	0	0	5	5
521850 Quirinópolis	0	0	0	4	4
521860 Rialma	0	0	0	2	2
521880 Rio Verde	0	0	0	9	9
521930 Santa Helena de Goiás	0	0	0	2	2
521940 Santa Rita do Araguaia	0	0	0	1	1
521975 Santo Antônio do Descoberto	0	0	0	1	1
522010 São Luís de Montes Belos	0	0	0	6	6
522020 São Miguel do Araguaia	0	0	0	2	2
522040 São Simão	0	0	0	2	2
522045 Senador Canedo	0	0	0	4	4
522050 Serranópolis	0	0	0	1	1
522060 Silvânia	0	0	0	1	1
522140 Trindade	0	0	0	12	12
522150 Turvânia	0	0	0	1	1
522157 Uirapuru	0	0	0	1	1
522160 Uruaçu	0	0	0	3	3
522185 Valparaíso de Goiás	0	0	0	6	6
522200 Vianópolis	0	0	0	2	2
521820 Porto Nacional (transf. p/TO)	0	0	0	1	1
530010 Brasília	1	4	0	205	210
Total	1063	2819	860	899	5641

MALÁRIA - Casos confirmados Notificados no Sistema de Informação de Agravos de Notificação - Sinan Net

Casos confirmados por Munic. Residência e result.parasitológi
Autoctone Mun Res: Não
Período:2007

Munic. Residência	Falciparum	F+FG	Vivax	F+V	Total
110001 Alta Floresta D'Oeste	0	1	0	0	1
110002 Ariquemes	0	1	0	4	5
110045 Buritis	0	0	2	0	2
110004 Cacoal	0	1	0	0	1
110094 Cujubim	0	0	1	0	1
110011 Jaru	0	2	1	0	3
110012 Ji-Paraná	0	1	0	0	1
110130 Mirante da Serra	0	1	0	0	1
110020 Porto Velho	2	3	1	2	8
110160 Theobroma	0	1	0	0	1
110170 Urupá	0	1	0	0	1
110030 Vilhena	0	0	0	2	2
120001 Acrelândia	0	0	1	0	1
120020 Cruzeiro do Sul	0	1	0	0	1
120040 Rio Branco	0	0	0	1	1
130110 Careiro	1	0	0	0	1
130260 Manaus	4	0	0	0	4
130353 Presidente Figueiredo	1	0	0	0	1
140010 Boa Vista	0	0	2	1	3
140060 São Luiz	0	0	0	1	1
150085 Anapu	0	1	0	0	1
150130 Barcarena	0	1	0	0	1
150380 Jacundá	0	1	0	0	1
150506 Novo Repartimento	1	0	0	0	1
150840 Xinguara	0	1	0	0	1
160050 Oiapoque	1	0	0	0	1
210462 Governador Luiz Rocha	1	0	0	0	1
210745 Olinda Nova do Maranhão	0	1	0	0	1
210910 Presidente Dutra	0	0	0	1	1
210980 Santa Helena	0	1	0	0	1
211050 São Bento	0	1	0	0	1
220120 Barras	2	0	0	0	2
220140 Barro Duro	1	0	0	0	1
220173 Betânia do Piauí	1	0	0	0	1
220217 Campo Largo do Piauí	3	0	0	0	3
220370 Esperantina	2	0	0	0	2
220510 Itaueira	1	0	0	0	1
220545 Joca Marques	1	0	0	0	1
220580 Luzilândia	9	0	0	0	9
220585 Madeiro	3	0	0	0	3
220880 Regeneração	1	0	0	0	1
221100 Teresina	4	0	0	0	4
220000 Município ignorado - PI	1	0	0	0	1

230110 Aracati	1	0	0	0	1
230240 Boa Viagem	2	0	0	0	2
230260 Camocim	1	0	0	0	1
230280 Canindé	1	0	0	0	1
230370 Caucaia	1	0	0	0	1
230440 Fortaleza	30	0	0	0	30
230550 Iguatu	1	0	0	0	1
230640 Itapipoca	1	0	0	0	1
230830 Milagres	1	0	0	0	1
231050 Pedra Branca	1	0	0	0	1
231290 Sobral	2	0	0	0	2
231310 Tabuleiro do Norte	1	0	0	0	1
231335 Tejuçuoca	1	0	0	0	1
231400 Várzea Alegre	1	0	0	0	1
240810 Natal	2	0	0	0	2
240325 Parnamirim	2	0	0	0	2
241340 Serra Negra do Norte	0	0	0	1	1
250030 Alagoa Grande	1	0	0	0	1
260290 Cabo de Santo Agostinho	0	1	0	0	1
260400 Carpina	1	0	0	0	1
260430 Cedro	2	0	0	0	2
260640 Gravatá	2	0	0	0	2
260790 Jaboatão dos Guararapes	1	0	0	0	1
260960 Olinda	1	0	0	0	1
261070 Paulista	2	0	0	0	2
261160 Recife	13	0	0	0	13
261250 Santa Cruz do Capibaribe	0	0	2	0	2
261580 Tupanatinga	1	0	0	0	1
270030 Arapiraca	3	0	0	0	3
270430 Maceió	2	0	0	0	2
280030 Aracaju	1	0	0	0	1
280700 São Miguel do Aleixo	1	0	0	0	1
290225 Arataca	1	0	0	0	1
290327 Barrocas	1	0	0	0	1
291050 Entre Rios	2	0	0	0	2
291072 Eunápolis	2	0	0	0	2
291080 Feira de Santana	1	0	0	0	1
291360 Ilhéus	2	0	0	0	2
291560 Itamaraju	1	0	0	0	1
292100 Mata de São João	1	0	0	0	1
292230 Muritiba	2	0	0	0	2
292520 Pojuca	3	0	0	0	3
292530 Porto Seguro	1	0	0	0	1
292600 Remanso	3	0	0	0	3
292740 Salvador	4	0	0	0	4
293135 Teixeira de Freitas	1	0	0	0	1
310350 Araguari	0	4	0	0	4
310400 Araxá	0	1	0	0	1
310420 Arcos	0	1	0	0	1
310450 Arinos	0	2	0	0	2

310560 Barbacena	0	1	0	0	1
310620 Belo Horizonte	0	14	0	0	14
310860 Brasília de Minas	0	1	0	0	1
310930 Buritis	0	1	0	0	1
311260 Capinópolis	0	1	0	0	1
311730 Conceição das Alagoas	0	1	0	0	1
311860 Contagem	0	2	0	0	2
312230 Divinópolis	0	1	0	0	1
312390 Entre Rios de Minas	0	1	0	0	1
312610 Formiga	0	1	0	0	1
312780 Grão Mogol	0	1	0	0	1
312830 Guaranésia	0	2	0	0	2
312890 Guimarânia	0	3	0	0	3
312980 Ibirité	0	1	0	0	1
313170 Itabira	0	1	0	0	1
313240 Itajubá	0	1	0	0	1
313300 Itamonte	0	1	0	0	1
313420 Ituiutaba	0	1	0	0	1
313655 José Raydan	0	2	0	0	2
313670 Juiz de Fora	0	1	0	0	1
313720 Lagoa da Prata	0	2	0	0	2
313760 Lagoa Santa	0	1	0	0	1
313820 Lavras	0	1	0	0	1
313840 Leopoldina	0	1	0	0	1
313960 Mantena	0	5	0	0	5
314170 Mesquita	0	1	0	0	1
314240 Moema	0	1	0	0	1
314310 Monte Carmelo	0	2	0	0	2
314480 Nova Lima	0	1	0	0	1
314537 Novorizonte	0	1	0	0	1
314560 Oliveira	0	1	0	0	1
314700 Paracatu	0	1	0	0	1
314800 Patos de Minas	0	1	0	0	1
314810 Patrocínio	0	2	0	0	2
315430 Resplendor	0	1	0	0	1
315460 Ribeirão das Neves	0	2	0	0	2
315690 Sacramento	0	1	0	0	1
315780 Santa Luzia	0	2	0	0	2
315820 Santa Maria do Suaçuí	0	6	0	0	6
316040 Santo Antônio do Monte	0	3	0	0	3
316095 São Domingos das Dores	0	1	0	0	1
316210 São Gotardo	0	2	0	0	2
316410 São Pedro do Suaçuí	0	1	0	0	1
316553 Sarzedo	0	1	0	0	1
316720 Sete Lagoas	0	1	0	0	1
317010 Uberaba	0	3	0	0	3
317020 Uberlândia	0	12	0	0	12
317040 Unaí	0	6	0	0	6
317065 Vargem Grande do Rio Pardo	0	1	0	0	1
317110 Veríssimo	0	2	0	0	2

317120 Vespasiano	0	1	0	0	1
320013 Águia Branca	0	1	0	0	1
320040 Anchieta	0	1	0	0	1
320060 Aracruz	0	1	0	0	1
320080 Baixo Guandu	0	2	0	0	2
320090 Barra de São Francisco	0	4	0	0	4
320100 Boa Esperança	0	2	0	0	2
320130 Cariacica	0	1	0	0	1
320150 Colatina	0	2	0	0	2
320170 Conceição do Castelo	0	1	0	0	1
320210 Ecoporanga	0	1	0	0	1
320225 Governador Lindenberg	0	1	0	0	1
320240 Guarapari	0	1	0	0	1
320260 Iconha	0	1	0	0	1
320305 Jaguaré	0	2	0	0	2
320320 Linhares	0	3	0	0	3
320390 Nova Venécia	0	1	0	0	1
320400 Pancas	0	1	0	0	1
320460 Santa Teresa	0	1	0	0	1
320470 São Gabriel da Palha	0	2	0	0	2
320490 São Mateus	0	4	0	0	4
320495 São Roque do Canaã	0	1	0	0	1
320500 Serra	0	4	0	0	4
320510 Viana	0	2	0	0	2
320515 Vila Pavão	0	4	0	0	4
320517 Vila Valério	0	1	0	0	1
320520 Vila Velha	0	5	0	0	5
320530 Vitória	0	3	0	0	3
330080 Cachoeiras de Macacu	0	1	0	0	1
330220 Itaperuna	0	1	0	0	1
330240 Macaé	0	4	0	0	4
330330 Niterói	0	4	0	0	4
330350 Nova Iguaçu	0	3	0	0	3
330360 Paracambi	0	1	0	0	1
330410 Porciúncula	0	2	0	0	2
330455 Rio de Janeiro	0	38	0	0	38
330580 Teresópolis	0	3	0	0	3
330610 Valença	0	2	0	0	2
350010 Adamantina	0	1	0	0	1
350040 Águas da Prata	0	1	0	0	1
350380 Artur Nogueira	0	1	0	0	1
350390 Arujá	0	2	0	0	2
350570 Barueri	0	2	0	0	2
350580 Bastos	0	1	0	0	1
350590 Batatais	0	2	0	0	2
350600 Bauru	0	2	0	0	2
350635 Bertioga	0	0	0	1	1
350730 Boracéia	0	1	0	0	1
350850 Caçapava	0	1	0	0	1
350900 Caieiras	0	1	0	0	1

350920 Cajamar	0	1	0	0	1
350950 Campinas	0	3	0	0	3
351060 Carapicuíba	0	1	0	0	1
351500 Embu	0	1	0	0	1
351750 Guapiaçu	0	1	0	0	1
351840 Guaratinguetá	0	1	0	0	1
351880 Guarulhos	0	3	0	0	3
351907 Hortolândia	0	3	0	0	3
352050 Indaiatuba	0	1	0	0	1
352220 Itapecerica da Serra	0	2	0	0	2
352250 Itapevi	0	1	0	0	1
352330 Itariri	0	1	0	0	1
352440 Jacareí	0	4	0	0	4
352470 Jaguariúna	0	2	0	0	2
352600 Junqueirópolis	0	2	0	0	2
352620 Juquitiba	0	1	0	0	1
352690 Limeira	0	3	0	0	3
352900 Marília	0	4	1	0	5
353060 Mogi das Cruzes	0	1	0	0	1
353070 Mogi Guaçu	0	1	0	0	1
353250 Neves Paulista	0	1	0	0	1
353440 Osasco	0	4	0	0	4
353500 Palestina	0	1	0	0	1
353550 Paraguaçu Paulista	0	1	0	0	1
353610 Pardinho	0	1	0	0	1
353650 Paulínia	0	1	0	0	1
353730 Penápolis	0	1	0	0	1
353870 Piracicaba	0	2	0	0	2
353920 Pirapozinho	0	2	0	0	2
353930 Pirassununga	0	1	0	0	1
353980 Poá	0	1	0	0	1
354105 Pratânia	0	1	0	0	1
354140 Presidente Prudente	0	5	0	0	5
354240 Regente Feijó	0	1	0	0	1
354340 Ribeirão Preto	0	1	0	0	1
354390 Rio Claro	0	2	0	0	2
354770 Santo Anastácio	0	1	0	0	1
354780 Santo André	0	1	0	0	1
354800 Santo Antônio de Posse	0	1	0	0	1
354870 São Bernardo do Campo	0	5	0	0	5
354910 São João da Boa Vista	0	1	0	0	1
354980 São José do Rio Preto	0	3	0	0	3
354990 São José dos Campos	0	1	0	0	1
354995 São Lourenço da Serra	0	1	0	0	1
355030 São Paulo	0	68	0	1	69
355100 São Vicente	0	1	0	0	1
355220 Sorocaba	0	2	0	0	2
355240 Sumaré	0	3	0	0	3
355480 Tremembé	0	1	0	0	1
355645 Vargem Grande Paulista	0	3	0	0	3

355670 Vinhedo	0	2	0	0	2
355710 Votuporanga	0	1	0	0	1
410050 Altônia	0	0	2	0	2
410140 Apucarana	0	0	1	0	1
410150 Arapongas	0	0	1	0	1
410180 Araucária	0	0	0	1	1
410190 Assaí	0	0	1	0	1
410200 Assis Chateaubriand	0	0	4	0	4
410305 Boa Vista da Aparecida	0	1	0	0	1
410345 Cafelândia	0	1	2	0	3
410400 Campina Grande do Sul	0	0	1	0	1
410420 Campo Largo	0	0	2	0	2
410450 Capanema	0	0	1	0	1
410480 Cascavel	0	0	14	0	14
410530 Céu Azul	0	0	2	0	2
410630 Corbélia	0	0	2	0	2
410650 Coronel Vivida	0	0	1	0	1
410660 Cruzeiro do Oeste	0	0	1	0	1
410690 Curitiba	0	0	16	0	16
410715 Diamante D'Oeste	0	0	1	0	1
410725 Douradina	0	0	1	0	1
410753 Entre Rios do Oeste	0	0	1	0	1
410765 Fazenda Rio Grande	0	0	2	0	2
410830 Foz do Iguaçu	0	0	22	0	22
410840 Francisco Beltrão	0	0	1	0	1
410860 Goioerê	0	0	1	0	1
410930 Guaraniaçu	0	0	1	0	1
410970 Ibaiti	0	0	2	0	2
411280 Joaquim Távora	0	0	1	0	1
411330 Laranjeiras do Sul	0	0	1	0	1
411370 Londrina	0	0	3	0	3
411400 Mamborê	0	0	1	0	1
411410 Mandaguaçu	0	1	0	0	1
411460 Marechal Cândido Rondon	0	0	2	0	2
411480 Marialva	0	0	2	0	2
411520 Maringá	0	0	2	0	2
411590 Mirador	0	0	1	0	1
411670 Nova Aurora	0	0	1	0	1
411840 Paranavaí	0	0	2	0	2
411990 Ponta Grossa	0	1	0	0	1
412090 Quedas do Iguaçu	0	0	0	1	1
412280 Salgado Filho	0	0	1	0	1
412350 Santa Helena	0	0	1	0	1
412405 Santa Terezinha de Itaipu	0	0	5	0	5
412550 São José dos Pinhais	0	0	1	0	1
412570 São Miguel do Iguaçu	0	0	5	0	5
412575 São Pedro do Iguaçu	0	0	1	0	1
412625 Sarandi	0	0	1	0	1
412770 Toledo	0	0	3	0	3
420010 Abelardo Luz	0	0	1	0	1

420030 Agronômica	0	0	1	0	1
420230 Biguaçu	0	0	1	0	1
420280 Braço do Norte	0	0	5	0	5
420380 Canoinhas	1	0	0	0	1
420390 Capinzal	0	0	1	0	1
420420 Chapecó	0	0	1	0	1
420430 Concórdia	0	1	3	0	4
420470 Cunha Porã	0	0	2	1	3
420540 Florianópolis	0	0	1	0	1
420650 Guaramirim	0	0	1	0	1
420660 Guarujá do Sul	0	0	1	0	1
420810 Itaiópolis	0	0	3	0	3
420840 Itapiranga	0	0	1	0	1
420890 Jaraguá do Sul	0	0	1	0	1
420900 Joaçaba	0	0	1	0	1
421100 Mondaí	1	0	1	0	2
421200 Palma Sola	0	0	1	0	1
421660 São José	0	0	1	0	1
421690 São Lourenço do Oeste	0	0	1	0	1
421720 São Miguel do Oeste	0	0	1	0	1
421760 Siderópolis	0	0	1	0	1
421820 Timbó	0	0	1	0	1
421917 Vargem Bonita	0	0	1	0	1
430460 Canoas	0	0	1	0	1
430463 Capão da Canoa	0	0	1	0	1
430790 Farroupilha	0	0	1	0	1
431180 Marau	0	0	1	0	1
431240 Montenegro	0	0	1	0	1
431430 Pejuçara	0	0	1	0	1
431490 Porto Alegre	0	0	2	0	2
431690 Santa Maria	0	0	1	0	1
431800 São Borja	0	0	1	0	1
431870 São Leopoldo	0	0	2	0	2
431900 São Marcos	0	0	1	0	1
432140 Tenente Portela	0	0	1	0	1
500070 Anastácio	0	0	0	1	1
500100 Aparecida do Taboado	0	0	0	1	1
500260 Camapuã	0	0	0	1	1
500270 Campo Grande	0	0	0	15	15
500350 Douradina	0	0	0	1	1
500370 Dourados	0	0	0	3	3
500460 Itaquiraí	0	0	0	1	1
500793 Sonora	0	0	0	1	1
500795 Tacuru	0	0	0	1	1
510025 Alta Floresta	0	0	0	1	1
510180 Barra do Garças	0	0	0	2	2
510325 Colniza	0	0	1	1	2
510629 Paranaíta	0	1	0	0	1
510740 São Pedro da Cipa	0	0	1	0	1
510795 Tangará da Serra	0	1	0	1	2

520110 Anápolis	0	1	0	4	5
520140 Aparecida de Goiânia	0	0	0	12	12
520215 Araguapaz	0	0	0	2	2
520450 Caldas Novas	0	0	0	1	1
520485 Campo Limpo de Goiás	0	0	0	1	1
520495 Campos Verdes	0	0	0	1	1
520840 Goianápolis	0	0	0	1	1
520860 Goianésia	0	0	0	1	1
520870 Goiânia	0	0	0	48	48
520890 Goiás	0	0	0	1	1
520920 Guapó	0	0	0	1	1
521000 Inhumas	0	0	0	1	1
521020 Iporá	0	0	0	1	1
521120 Itapuranga	0	0	0	1	1
521150 Itumbiara	0	0	0	1	1
521250 Luziânia	0	0	0	1	1
521308 Minaçu	0	0	0	1	1
521310 Mineiros	0	0	0	1	1
521800 Porangatu	0	0	0	1	1
521850 Quirinópolis	0	0	0	1	1
522010 São Luís de Montes Belos	0	0	0	2	2
522020 São Miguel do Araguaia	0	0	0	1	1
522045 Senador Canedo	0	0	0	1	1
522060 Silvânia	0	0	0	1	1
522140 Trindade	0	0	0	3	3
522185 Valparaíso de Goiás	0	0	0	1	1
530010 Brasília	0	0	0	34	34
Total	147	427	176	173	923

MALÁRIA - Casos confirmados Notificados no Sistema de Informação de Agravos de Notificação - Sinan Net

Casos confirmados por Munic. Residência e result.parasitológi
Autoctone Mun Res: Não
Período:2008

Munic. Residência	Falciparum	F+FG	Vivax	F+V	Total
110040 Alto Paraíso	0	0	1	0	1
110011 Jaru	0	1	0	0	1
110033 Nova Mamoré	0	1	0	0	1
110015 Ouro Preto do Oeste	0	1	0	0	1
110020 Porto Velho	1	3	0	0	4
120040 Rio Branco	0	1	0	0	1
130014 Apuí	0	0	1	0	1
130260 Manaus	0	1	0	0	1
130380 São Gabriel da Cachoeira	0	1	0	0	1
140047 Rorainópolis	0	1	0	0	1
150553 Parauapebas	0	1	0	0	1
150730 São Félix do Xingu	0	1	0	0	1
210530 Imperatriz	0	0	0	1	1
210570 Lago da Pedra	1	0	0	0	1
211220 Timon	2	0	0	0	2
220120 Barras	3	0	0	0	3
220370 Esperantina	1	0	0	0	1
220540 Joaquim Pires	2	0	0	0	2
220580 Luzilândia	16	0	0	0	16
220610 Matias Olímpio	1	0	0	0	1
220850 Porto	1	0	0	0	1
221100 Teresina	3	0	0	0	3
230110 Aracati	1	0	0	0	1
230340 Carnaubal	1	0	0	0	1
230370 Caucaia	2	0	0	0	2
230410 Crateús	1	0	0	0	1
230440 Fortaleza	11	0	0	0	11
230470 Granja	1	0	0	0	1
231350 Trairi	1	0	0	0	1
240020 Açu	2	0	0	0	2
240150 Barcelona	1	0	0	0	1
240580 João Câmara	1	0	0	0	1
240810 Natal	1	0	0	0	1
241200 São Gonçalo do Amarante	1	0	0	0	1
251080 Patos	0	0	0	1	1
260400 Carpina	1	0	0	0	1
260570 Floresta	1	0	0	0	1
260790 Jaboatão dos Guararapes	5	0	0	0	5
260960 Olinda	1	0	0	0	1
261060 Paudalho	1	0	0	0	1
261160 Recife	9	1	0	0	10
270430 Maceió	2	0	0	0	2
270470 Marechal Deodoro	1	0	0	0	1

280067 Boquim	1	0	0	0	1
290070 Alagoinhas	1	0	0	0	1
290327 Barrocas	0	1	0	0	1
291072 Eunápolis	1	0	0	0	1
291190 Iaçu	4	0	0	0	4
291360 Ilhéus	1	0	0	0	1
291480 Itabuna	0	1	0	0	1
292200 Mucuri	1	0	0	0	1
292530 Porto Seguro	2	0	0	0	2
292740 Salvador	10	0	0	0	10
293070 Simões Filho	1	0	0	0	1
293150 Teofilândia	0	0	0	1	1
310350 Araguari	0	4	0	0	4
310560 Barbacena	0	2	0	0	2
310620 Belo Horizonte	0	18	0	0	18
310670 Betim	0	2	0	0	2
310740 Bom Despacho	0	1	0	0	1
310890 Brasópolis	0	1	0	0	1
311140 Campo Florido	0	2	0	0	2
311535 Catas Altas	0	1	0	0	1
311860 Contagem	0	6	0	0	6
311930 Coromandel	0	1	0	0	1
312240 Divisa Nova	0	1	0	0	1
312610 Formiga	0	2	0	0	2
312710 Frutal	0	2	0	0	2
312770 Governador Valadares	0	5	0	0	5
312890 Guimarânia	0	1	0	0	1
312980 Ibirité	0	3	0	0	3
313130 Ipatinga	0	1	0	0	1
313170 Itabira	0	1	0	0	1
313180 Itabirinha	0	2	0	0	2
313190 Itabirito	0	1	0	0	1
313210 Itacarambi	0	1	0	0	1
313420 Ituiutaba	0	2	0	0	2
313505 Jaíba	0	1	0	0	1
313665 Juatuba	0	1	0	0	1
313760 Lagoa Santa	0	1	0	0	1
313880 Luz	0	2	0	0	2
313960 Mantena	0	1	0	0	1
314390 Muriaé	0	1	0	0	1
314480 Nova Lima	0	2	0	0	2
314500 Nova Ponte	0	1	0	0	1
314650 Pains	0	1	0	0	1
314800 Patos de Minas	0	2	0	0	2
314810 Patrocínio	0	1	0	0	1
315220 Porteirinha	0	2	0	0	2
315250 Pouso Alegre	0	1	0	0	1
315460 Ribeirão das Neves	0	2	0	0	2
315780 Santa Luzia	0	2	0	0	2
316040 Santo Antônio do Monte	0	1	0	0	1

316250 São João del Rei	0	3	0	0	3
316294 São José da Barra	0	1	0	0	1
316670 Serra dos Aimorés	0	1	0	0	1
317010 Uberaba	0	1	0	0	1
317020 Uberlândia	0	5	0	0	5
317040 Unaí	0	2	0	0	2
317130 Viçosa	0	2	0	0	2
320090 Barra de São Francisco	0	2	0	0	2
320130 Cariacica	0	6	0	0	6
320150 Colatina	0	3	0	1	4
320270 Itaguaçu	0	3	0	0	3
320320 Linhares	0	8	0	0	8
320440 Rio Novo do Sul	0	1	0	0	1
320455 Santa Maria de Jetibá	0	1	0	0	1
320460 Santa Teresa	0	1	0	0	1
320470 São Gabriel da Palha	0	1	0	0	1
320490 São Mateus	0	1	0	0	1
320495 São Roque do Canaã	0	1	0	0	1
320500 Serra	0	6	0	0	6
320506 Venda Nova do Imigrante	0	1	0	0	1
320517 Vila Valério	0	1	0	0	1
320520 Vila Velha	0	2	0	0	2
320530 Vitória	0	6	0	0	6
330020 Araruama	0	1	0	0	1
330060 Bom Jesus do Itabapoana	0	1	0	0	1
330170 Duque de Caxias	0	3	0	0	3
330200 Itaguaí	0	1	0	0	1
330240 Macaé	0	1	0	0	1
330270 Maricá	0	1	0	0	1
330330 Niterói	0	2	0	0	2
330350 Nova Iguaçu	0	1	0	0	1
330430 Rio Bonito	0	1	0	0	1
330440 Rio Claro	0	1	0	0	1
330455 Rio de Janeiro	0	34	0	1	35
330490 São Gonçalo	0	2	0	0	2
330580 Teresópolis	0	1	0	0	1
330610 Valença	0	1	0	0	1
350380 Artur Nogueira	0	1	0	0	1
350550 Barretos	0	2	0	0	2
350560 Barrinha	0	1	0	0	1
350570 Barueri	0	2	0	0	2
350600 Bauru	0	1	0	0	1
350710 Bom Jesus dos Perdões	0	2	0	0	2
350750 Botucatu	0	2	0	0	2
350780 Brodowski	0	1	0	0	1
350790 Brotas	0	1	0	0	1
350900 Caieiras	0	1	0	0	1
350950 Campinas	0	3	0	0	3
351060 Carapicuíba	0	3	0	0	3
351380 Diadema	0	2	0	0	2

351470 Echaporã	0	1	0	0	1
351670 Garça	0	2	0	0	2
351710 Glicério	0	1	0	0	1
351750 Guapiaçu	0	1	0	0	1
351880 Guarulhos	0	2	0	0	2
351907 Hortolândia	0	0	1	0	1
352140 Iracemápolis	0	1	0	0	1
352240 Itapeva	0	1	0	0	1
352390 Itu	0	1	0	0	1
352410 Ituverava	0	1	0	0	1
352480 Jales	0	1	0	0	1
352590 Jundiaí	0	3	0	0	3
352690 Limeira	0	1	0	0	1
352850 Mairiporã	0	1	0	0	1
352930 Matão	0	1	0	0	1
352940 Mauá	0	2	0	0	2
353010 Mirandópolis	0	1	0	0	1
353440 Osasco	0	2	0	0	2
353460 Osvaldo Cruz	0	1	0	0	1
353500 Palestina	0	1	0	0	1
353600 Parapuã	0	1	0	0	1
353650 Paulínia	0	1	0	0	1
353780 Piedade	0	2	0	0	2
353890 Pirajuí	0	1	0	0	1
354140 Presidente Prudente	0	1	0	0	1
354340 Ribeirão Preto	0	4	0	0	4
354400 Rio das Pedras	0	1	0	0	1
354680 Santa Isabel	0	1	0	0	1
354730 Santana de Parnaíba	0	1	0	0	1
354780 Santo André	0	2	0	0	2
354870 São Bernardo do Campo	0	4	0	0	4
354990 São José dos Campos	0	1	0	0	1
355010 São Manuel	0	2	0	0	2
355030 São Paulo	0	40	0	0	40
355220 Sorocaba	0	2	0	0	2
355240 Sumaré	0	3	0	0	3
355280 Taboão da Serra	0	1	0	0	1
355420 Tejupá	0	1	0	0	1
355620 Valinhos	0	1	0	0	1
355710 Votuporanga	0	1	0	0	1
410100 Ampére	0	0	1	0	1
410120 Antonina	0	0	1	0	1
410150 Arapongas	0	0	1	0	1
410200 Assis Chateaubriand	0	0	1	0	1
410480 Cascavel	0	0	7	0	7
410530 Céu Azul	0	0	2	0	2
410630 Corbélia	0	0	1	0	1
410690 Curitiba	0	0	16	0	16
410765 Fazenda Rio Grande	0	0	1	0	1
410830 Foz do Iguaçu	0	0	3	0	3

410840 Francisco Beltrão	0	0	2	0	2
410930 Guaraniaçu	0	0	2	0	2
410940 Guarapuava	0	0	2	0	2
411460 Marechal Cândido Rondon	0	0	1	0	1
411520 Maringá	0	2	6	0	8
411580 Medianeira	0	0	1	0	1
411740 Ourizona	0	0	1	0	1
411850 Pato Branco	0	0	1	0	1
411910 Piên	0	0	1	0	1
412100 Querência do Norte	0	0	1	0	1
412140 Realeza	0	0	2	0	2
412380 Santa Izabel do Oeste	0	0	1	0	1
412405 Santa Terezinha de Itaipu	0	0	2	0	2
412550 São José dos Pinhais	0	0	3	0	3
412570 São Miguel do Iguaçu	0	0	2	0	2
412770 Toledo	0	0	2	0	2
412800 Ubiratã	0	0	1	0	1
412860 Verê	0	0	1	0	1
420200 Balneário Camboriú	0	0	1	0	1
420240 Blumenau	0	0	1	0	1
420320 Camboriú	0	0	1	0	1
420380 Canoinhas	0	0	1	0	1
420430 Concórdia	0	0	2	0	2
420540 Florianópolis	0	0	1	0	1
420660 Guarujá do Sul	0	0	1	0	1
420750 Indaial	0	0	1	0	1
420910 Joinville	0	0	1	0	1
421480 Rio do Sul	0	0	1	0	1
421660 São José	0	0	1	0	1
421760 Siderópolis	0	0	0	1	1
421820 Timbó	0	0	1	0	1
430510 Caxias do Sul	0	0	1	0	1
430545 Cidreira	0	0	1	0	1
430570 Condor	0	0	1	0	1
430610 Cruz Alta	0	0	1	0	1
430845 Fortaleza dos Valos	0	0	1	0	1
430860 Garibaldi	0	0	1	0	1
430950 Guarani das Missões	0	0	1	0	1
431000 Ibirubá	0	0	0	1	1
431490 Porto Alegre	0	0	1	0	1
431630 Roque Gonzales	0	0	1	0	1
431760 Santo Antônio da Patrulha	0	0	1	0	1
431870 São Leopoldo	0	0	1	0	1
432195 Trindade do Sul	0	0	1	0	1
500070 Anastácio	0	0	0	1	1
500100 Aparecida do Taboado	0	0	0	1	1
500270 Campo Grande	0	0	0	15	15
500320 Corumbá	0	0	0	1	1
500325 Costa Rica	0	0	0	1	1
500460 Itaquiraí	0	0	0	1	1

500568 Mundo Novo	0	0	0	1	1
500620 Nova Andradina	0	0	0	1	1
500793 Sonora	0	0	0	1	1
510030 Alto Araguaia	0	0	0	1	1
510340 Cuiabá	0	1	0	0	1
510385 Gaúcha do Norte	0	1	0	0	1
510410 Guarantã do Norte	0	0	0	1	1
510792 Sorriso	0	0	0	1	1
520110 Anápolis	0	1	0	3	4
520140 Aparecida de Goiânia	0	0	0	5	5
520393 Buriti de Goiás	0	0	0	1	1
520450 Caldas Novas	0	0	0	1	1
520570 Córrego do Ouro	0	0	0	1	1
520590 Corumbaíba	0	0	0	1	1
520620 Cristalina	0	0	0	1	1
520870 Goiânia	0	2	0	23	25
521020 Iporá	0	0	0	1	1
521190 Jataí	0	0	0	1	1
521300 Maurilândia	0	0	0	1	1
521310 Mineiros	0	0	0	2	2
521940 Santa Rita do Araguaia	0	0	0	1	1
522040 São Simão	0	0	0	1	1
522140 Trindade	0	0	0	1	1
522157 Uirapuru	0	0	0	1	1
522160 Uruaçu	0	0	0	1	1
522200 Vianópolis	0	0	0	1	1
530010 Brasília	0	0	0	18	18
Total	98	332	94	98	622

MALÁRIA - Casos confirmados Notificados no Sistema de Informação de Agravos de Notificação - Sinan Net

Casos confirmados por Munic. Residência e result.parasitológi
Autoctone Mun Res: Não
Período:2009

Munic. Residência	Falciparum	F+FG	Vivax	F+V	Total
110002 Ariquemes	0	0	2	0	2
110004 Cacoal	0	2	0	0	2
110009 Espigão D'Oeste	0	1	0	0	1
110012 Ji-Paraná	0	0	1	0	1
110013 Machadinho D'Oeste	0	1	0	0	1
110020 Porto Velho	0	1	1	1	3
130260 Manaus	1	0	0	0	1
130270 Manicoré	0	1	0	0	1
150080 Ananindeua	1	0	0	0	1
150140 Belém	0	1	0	0	1
150157 Bom Jesus do Tocantins	1	0	0	0	1
150360 Itaituba	1	0	0	0	1
150380 Jacundá	0	0	0	1	1
150550 Paragominas	1	0	0	0	1
210360 Coroatá	1	0	0	0	1
210910 Presidente Dutra	1	0	0	0	1
211130 São Luís	0	2	0	0	2
220150 Batalha	1	0	0	0	1
220177 Boa Hora	1	0	0	0	1
220205 Cabeceiras do Piauí	1	0	0	0	1
220370 Esperantina	1	0	0	0	1
220385 Floresta do Piauí	0	1	0	0	1
220390 Floriano	1	0	0	0	1
220540 Joaquim Pires	1	0	0	0	1
220545 Joca Marques	1	0	0	0	1
220580 Luzilândia	12	0	0	0	12
220585 Madeiro	1	0	0	0	1
220640 Monsenhor Gil	1	0	0	0	1
220800 Picos	1	0	0	0	1
220840 Piripiri	1	0	0	0	1
220850 Porto	1	0	0	0	1
220880 Regeneração	2	0	0	0	2
220980 São Gonçalo do Piauí	1	0	0	0	1
221100 Teresina	3	0	0	0	3
230250 Brejo Santo	1	0	0	0	1
230440 Fortaleza	10	0	0	0	10
230445 Fortim	2	0	0	0	2
230640 Itapipoca	1	0	0	0	1
230960 Pacajus	1	0	0	0	1
231150 Quixeré	1	0	0	0	1
231230 São Benedito	1	0	0	0	1
231290 Sobral	2	0	0	0	2
231310 Tabuleiro do Norte	3	0	0	0	3

240200 Caicó	1	0	0	0	1
240710 Macaíba	1	0	0	0	1
240800 Mossoró	1	0	0	0	1
240810 Natal	6	0	0	0	6
240325 Parnamirim	1	0	0	0	1
241220 São José de Mipibu	2	0	0	0	2
250400 Campina Grande	7	1	0	0	8
250430 Catolé do Rocha	2	0	0	0	2
260005 Abreu e Lima	1	0	0	0	1
260290 Cabo de Santo Agostinho	2	1	0	0	3
260410 Caruaru	2	0	0	0	2
260790 Jaboatão dos Guararapes	4	0	0	0	4
260850 Lagoa do Itaenga	1	0	0	0	1
260960 Olinda	1	1	0	0	2
261160 Recife	17	0	0	0	17
270020 Anadia	1	0	0	0	1
270050 Barra de Santo Antônio	1	0	0	0	1
270240 Delmiro Gouveia	1	0	0	0	1
270430 Maceió	3	0	0	0	3
270460 Maravilha	1	0	0	0	1
270860 São Miguel dos Campos	1	0	0	0	1
280030 Aracaju	2	0	0	0	2
280120 Canindé de São Francisco	1	0	0	0	1
280210 Estância	1	0	0	0	1
280480 Nossa Senhora do Socorro	1	0	0	0	1
290800 Coaraci	1	0	0	0	1
290820 Conceição da Feira	1	0	0	0	1
291010 Dom Basílio	0	1	0	0	1
291465 Itabela	1	0	0	0	1
291480 Itabuna	1	0	0	0	1
292530 Porto Seguro	1	0	0	0	1
292740 Salvador	5	2	0	0	7
293010 Senhor do Bonfim	1	0	0	0	1
293070 Simões Filho	1	0	0	0	1
293135 Teixeira de Freitas	1	0	0	0	1
310350 Araguari	0	2	0	0	2
310400 Araxá	0	2	0	0	2
310560 Barbacena	0	1	0	0	1
310620 Belo Horizonte	0	7	0	0	7
310670 Betim	0	6	0	0	6
310690 Bicas	0	1	0	0	1
310900 Brumadinho	0	3	0	0	3
310980 Cachoeira Dourada	0	1	0	0	1
311840 Conselheiro Pena	0	1	0	0	1
311860 Contagem	0	5	0	0	5
311910 Corinto	0	1	0	0	1
312090 Curvelo	0	2	0	0	2
312230 Divinópolis	0	5	0	0	5
312410 Esmeraldas	0	2	0	0	2
312480 Estrela do Sul	0	1	0	0	1

312750 Gonzaga	0	1	0	0	1
312780 Grão Mogol	0	1	0	0	1
312980 Ibirité	0	3	0	0	3
313130 Ipatinga	0	1	0	0	1
313370 Itatiaiuçu	0	1	0	0	1
313480 Jacuí	0	1	0	0	1
313510 Janaúba	0	1	0	0	1
313670 Juiz de Fora	0	2	0	0	2
313820 Lavras	0	1	0	0	1
314000 Mariana	0	2	0	0	2
314590 Ouro Branco	0	1	0	0	1
314610 Ouro Preto	0	1	0	0	1
314710 Pará de Minas	0	1	0	0	1
314700 Paracatu	0	4	0	0	4
315210 Ponte Nova	0	1	0	0	1
315290 Pratápolis	0	1	0	0	1
315390 Raposos	0	0	1	0	1
315460 Ribeirão das Neves	0	1	0	0	1
315590 Rio Preto	0	1	0	0	1
315670 Sabará	0	1	0	0	1
315720 Santa Bárbara	0	1	0	0	1
315780 Santa Luzia	0	1	0	0	1
316292 São Joaquim de Bicas	0	3	0	0	3
316553 Sarzedo	0	2	0	0	2
317010 Uberaba	0	2	0	0	2
317020 Uberlândia	0	2	0	1	3
317070 Varginha	0	1	0	0	1
320016 Água Doce do Norte	0	1	0	0	1
320060 Aracruz	1	0	0	0	1
320080 Baixo Guandu	0	2	0	0	2
320130 Cariacica	0	0	0	1	1
320240 Guarapari	0	1	0	0	1
320280 Itapemirim	0	1	0	0	1
320313 João Neiva	0	2	0	0	2
320320 Linhares	0	3	0	0	3
320334 Marechal Floriano	0	1	0	0	1
320390 Nova Venécia	0	2	0	0	2
320460 Santa Teresa	0	1	0	0	1
320490 São Mateus	0	2	0	0	2
320495 São Roque do Canaã	0	2	0	0	2
320500 Serra	0	3	0	0	3
320517 Vila Valério	0	1	0	0	1
320520 Vila Velha	0	2	0	0	2
320530 Vitória	0	4	0	0	4
330070 Cabo Frio	0	1	0	0	1
330080 Cachoeiras de Macacu	0	1	0	0	1
330240 Macaé	0	2	0	0	2
330350 Nova Iguaçu	0	2	0	0	2
330420 Resende	0	1	0	0	1
330452 Rio das Ostras	0	2	0	0	2

330455 Rio de Janeiro	0	47	0	0	47
330490 São Gonçalo	0	2	0	0	2
330500 São João da Barra	0	1	0	0	1
330510 São João de Meriti	0	1	0	0	1
350160 Americana	0	2	0	0	2
350210 Andradina	0	1	0	0	1
350635 Bertioga	0	2	0	0	2
350920 Cajamar	0	2	0	0	2
350950 Campinas	0	4	0	0	4
351060 Carapicuíba	0	1	0	0	1
351570 Ferraz de Vasconcelos	0	1	0	0	1
351610 Florínia	0	1	0	0	1
351630 Francisco Morato	0	3	0	0	3
351640 Franco da Rocha	0	1	0	0	1
351670 Garça	0	1	0	0	1
351740 Guaíra	0	1	0	0	1
351820 Guararapes	0	1	0	0	1
351880 Guarulhos	0	1	0	0	1
352040 Ilhabela	0	2	0	0	2
352170 Itaberá	0	1	0	0	1
352220 Itapecerica da Serra	0	1	0	0	1
352340 Itatiba	0	1	0	0	1
352470 Jaguariúna	0	1	0	0	1
352500 Jandira	0	1	0	0	1
352520 Jarinu	0	1	0	0	1
352620 Juquitiba	0	1	0	0	1
352690 Limeira	0	4	0	0	4
352900 Marília	0	1	0	0	1
352940 Mauá	0	1	0	0	1
353060 Mogi das Cruzes	0	1	0	0	1
353070 Mogi Guaçu	0	2	0	0	2
353110 Mongaguá	0	1	0	0	1
353260 Nhandeara	0	1	0	0	1
353440 Osasco	0	3	0	0	3
353530 Palmital	0	1	0	0	1
353730 Penápolis	0	2	0	0	2
353760 Peruíbe	0	1	0	0	1
353860 Piracaia	0	2	0	0	2
354025 Pontalinda	0	2	0	0	2
354075 Potim	0	2	0	0	2
354100 Praia Grande	0	1	0	0	1
354140 Presidente Prudente	0	0	1	0	1
354340 Ribeirão Preto	0	1	0	0	1
354580 Santa Bárbara d'Oeste	0	2	0	0	2
354730 Santana de Parnaíba	0	2	0	0	2
354780 Santo André	0	3	0	0	3
354870 São Bernardo do Campo	0	4	0	0	4
354880 São Caetano do Sul	0	1	0	0	1
354980 São José do Rio Preto	0	1	0	0	1
354990 São José dos Campos	0	1	0	0	1

355030 São Paulo	0	53	0	1	54
355070 São Sebastião	0	2	0	0	2
355100 São Vicente	0	2	0	0	2
355650 Várzea Paulista	0	1	0	0	1
355710 Votuporanga	0	1	0	0	1
410140 Apucarana	0	0	1	0	1
410350 Califórnia	0	0	2	0	2
410480 Cascavel	0	0	2	0	2
410580 Colombo	0	0	1	0	1
410630 Corbélia	0	0	1	0	1
410660 Cruzeiro do Oeste	0	0	1	0	1
410690 Curitiba	0	0	11	0	11
410820 Formosa do Oeste	0	0	2	0	2
410830 Foz do Iguaçu	0	0	6	0	6
410840 Francisco Beltrão	0	1	0	0	1
410890 Guairaçá	0	0	1	0	1
411070 Irati	0	0	1	0	1
411160 Ivatuba	0	0	1	0	1
411330 Laranjeiras do Sul	0	0	1	0	1
411480 Marialva	0	0	1	0	1
411520 Maringá	0	0	5	0	5
411575 Mauá da Serra	0	0	1	0	1
411690 Nova Esperança	0	0	1	0	1
411725 Nova Prata do Iguaçu	0	0	1	0	1
411840 Paranavaí	0	1	0	0	1
411850 Pato Branco	0	0	1	0	1
411990 Ponta Grossa	0	0	2	0	2
412100 Querência do Norte	0	0	2	0	2
412140 Realeza	0	0	2	0	2
412350 Santa Helena	0	0	19	0	19
412570 São Miguel do Iguaçu	0	0	3	0	3
412810 Umuarama	0	0	4	0	4
420208 Bandeirante	0	1	0	0	1
420240 Blumenau	0	0	1	0	1
420430 Concórdia	0	0	1	0	1
420470 Cunha Porã	0	0	1	0	1
420540 Florianópolis	0	1	1	0	2
420660 Guarujá do Sul	0	0	2	0	2
420730 Imbituba	0	1	1	0	2
420830 Itapema	0	0	1	0	1
420890 Jaraguá do Sul	0	0	1	0	1
421480 Rio do Sul	0	0	1	0	1
421620 São Francisco do Sul	0	0	1	0	1
421690 São Lourenço do Oeste	0	0	1	0	1
421720 São Miguel do Oeste	0	0	1	0	1
421760 Siderópolis	0	0	1	0	1
421970 Xaxim	0	0	1	0	1
430610 Cruz Alta	0	0	1	0	1
430860 Garibaldi	0	0	1	0	1
431140 Lajeado	0	0	1	0	1

431490 Porto Alegre	0	0	1	0	1
431720 Santa Rosa	0	0	2	0	2
500070 Anastácio	0	0	0	1	1
500270 Campo Grande	0	0	0	20	20
500325 Costa Rica	0	0	0	1	1
500370 Dourados	0	0	0	2	2
500375 Eldorado	0	0	0	1	1
500500 Jardim	0	0	0	1	1
500620 Nova Andradina	0	0	0	1	1
500790 Sidrolândia	0	0	0	1	1
500797 Taquarussu	0	0	0	2	2
510330 Comodoro	0	0	1	0	1
510840 Várzea Grande	0	1	0	0	1
510000 Município ignorado - MT	0	1	0	0	1
520025 Águas Lindas de Goiás	0	0	0	1	1
520055 Alto Horizonte	0	0	0	1	1
520110 Anápolis	0	0	0	3	3
520140 Aparecida de Goiânia	0	0	0	6	6
520870 Goiânia	1	0	0	18	19
521150 Itumbiara	0	0	0	1	1
521190 Jataí	0	0	0	3	3
521250 Luziânia	0	0	0	1	1
521308 Minaçu	0	0	0	1	1
521310 Mineiros	0	0	0	1	1
521720 Piranhas	0	0	0	1	1
522050 Serranópolis	0	0	0	1	1
522140 Trindade	0	0	0	2	2
522160 Uruaçu	0	0	0	1	1
522200 Vianópolis	0	0	0	1	1
530010 Brasília	0	0	0	24	24
Total	136	319	101	101	657

MALÁRIA - Casos confirmados Notificados no Sistema de Informação de Agravos de Notificação - Sinan Net
Casos confirmados por Munic. Residência e result.parasitológi
Autoctone Mun Res: Não
Período:2010

Munic. Residência	Falciparum	F+FG	Vivax	F+V	Total
110004 Cacoal	0	0	0	1	1
110070 Campo Novo de Rondônia	0	1	0	0	1
110011 Jaru	0	0	1	0	1
110020 Porto Velho	1	2	0	1	4
110026 Rio Crespo	0	0	1	0	1
110030 Vilhena	0	0	0	1	1
120020 Cruzeiro do Sul	0	1	0	0	1
120080 Porto Acre	0	1	0	0	1
130260 Manaus	0	2	0	0	2
130300 Nhamundá	0	0	1	0	1
140010 Boa Vista	0	1	0	0	1
150380 Jacundá	0	0	1	0	1
150548 Pacajá	0	1	0	0	1
210120 Bacabal	1	0	0	0	1
210390 Duque Bacelar	1	0	0	0	1
210820 Pedreiras	1	0	0	0	1
211220 Timon	2	0	0	0	2
211230 Tuntum	1	0	0	0	1
220020 Água Branca	1	0	0	0	1
220040 Altos	1	0	0	0	1
220120 Barras	2	0	0	0	2
220150 Batalha	1	0	0	0	1
220205 Cabeceiras do Piauí	1	0	0	0	1
220240 Capitão de Campos	1	0	0	0	1
220330 Demerval Lobão	1	0	0	0	1
220540 Joaquim Pires	1	0	0	0	1
220558 Lagoa do Piauí	1	0	0	0	1
220580 Luzilândia	8	0	0	0	8
220610 Matias Olímpio	3	0	0	0	3
220640 Monsenhor Gil	1	0	0	0	1
220770 Parnaíba	1	0	0	0	1
220840 Piripiri	6	0	0	0	6
220980 São Gonçalo do Piauí	2	0	0	0	2
220997 São João do Arraial	1	0	0	0	1
221100 Teresina	17	0	0	0	17
230075 Amontada	1	0	0	0	1
230240 Boa Viagem	3	0	0	0	3
230370 Caucaia	2	0	0	0	2
230400 Coreaú	3	0	0	0	3
230440 Fortaleza	16	0	0	0	16
230523 Horizonte	1	0	0	0	1
230550 Iguatu	1	0	0	0	1
230610 Irauçuba	5	0	0	0	5
230760 Limoeiro do Norte	1	0	0	0	1

231020 Paracuru	1	0	0	0	1
231030 Parambu	2	0	0	0	2
231240 São Gonçalo do Amarante	3	0	0	0	3
231280 Senador Sá	1	0	0	0	1
231300 Solonópole	2	0	0	0	2
231310 Tabuleiro do Norte	2	0	0	0	2
231410 Viçosa do Ceará	2	0	0	0	2
240200 Caicó	1	0	0	0	1
240710 Macaíba	1	0	0	0	1
240810 Natal	4	0	0	0	4
240850 Ouro Branco	1	0	0	0	1
240325 Parnamirim	1	0	0	0	1
241200 São Gonçalo do Amarante	3	0	0	0	3
241300 São Vicente	1	0	0	0	1
241340 Serra Negra do Norte	1	0	0	0	1
241400 Tangará	1	0	0	0	1
250180 Bayeux	1	0	0	0	1
250370 Cajazeiras	1	0	0	0	1
250430 Catolé do Rocha	4	0	0	0	4
250750 João Pessoa	2	0	0	0	2
250905 Marcação	1	0	0	0	1
251080 Patos	1	0	0	0	1
251230 Princesa Isabel	1	0	0	0	1
251370 Santa Rita	1	0	0	0	1
251540 Seridó	1	0	0	0	1
251610 Soledade	2	0	0	0	2
260345 Camaragibe	1	0	0	0	1
260840 Jurema	1	0	0	0	1
260990 Ouricuri	2	0	0	0	2
261110 Petrolina	1	0	0	0	1
261160 Recife	4	0	0	0	4
261350 São José do Belmonte	1	0	0	0	1
261360 São José do Egito	1	0	0	0	1
270030 Arapiraca	1	0	0	0	1
270240 Delmiro Gouveia	2	0	0	0	2
270430 Maceió	1	0	0	0	1
280030 Aracaju	1	1	0	0	2
280060 Barra dos Coqueiros	1	0	0	0	1
280120 Canindé de São Francisco	2	0	0	0	2
280450 Nossa Senhora da Glória	1	0	0	0	1
280480 Nossa Senhora do Socorro	1	0	0	0	1
280540 Poço Redondo	1	0	0	0	1
290070 Alagoinhas	1	0	0	0	1
290790 Cipó	1	0	0	0	1
291080 Feira de Santana	1	0	0	0	1
291185 Heliópolis	1	0	0	0	1
291320 Ibotirama	1	0	0	0	1
291470 Itaberaba	1	0	0	0	1
291480 Itabuna	1	0	0	0	1
291560 Itamaraju	1	0	0	0	1

292230 Muritiba	1	0	0	0	1
292300 Nova Viçosa	0	1	0	0	1
292400 Paulo Afonso	1	0	0	0	1
292740 Salvador	4	0	0	0	4
293077 Sobradinho	2	0	0	0	2
293190 Tucano	1	0	0	0	1
310050 Açucena	0	1	0	0	1
310350 Araguari	0	3	0	0	3
310400 Araxá	0	2	0	0	2
310450 Arinos	0	1	0	0	1
310490 Baependi	0	1	0	0	1
310510 Bambuí	0	1	0	0	1
310560 Barbacena	0	2	0	0	2
310620 Belo Horizonte	0	17	0	0	17
310670 Betim	0	2	0	0	2
310900 Brumadinho	0	2	0	0	2
311000 Caeté	0	1	0	0	1
311860 Contagem	0	1	0	0	1
311930 Coromandel	0	1	0	0	1
311940 Coronel Fabriciano	0	1	0	0	1
312230 Divinópolis	0	4	0	0	4
312410 Esmeraldas	0	1	0	0	1
312610 Formiga	0	1	0	0	1
312710 Frutal	0	7	0	0	7
312770 Governador Valadares	0	2	0	0	2
312870 Guaxupé	0	1	0	0	1
313010 Igarapé	0	1	0	0	1
313120 Ipanema	0	1	0	0	1
313130 Ipatinga	0	1	0	0	1
313170 Itabira	0	2	0	0	2
313420 Ituiutaba	0	2	0	0	2
313690 Juruaia	0	1	0	0	1
313720 Lagoa da Prata	0	1	0	0	1
314000 Mariana	0	2	0	0	2
314390 Muriaé	0	2	0	0	2
314460 Nepomuceno	0	1	0	0	1
314470 Nova Era	0	2	0	0	2
314480 Nova Lima	0	2	0	0	2
314610 Ouro Preto	0	1	0	0	1
314630 Padre Paraíso	0	1	0	0	1
314700 Paracatu	0	2	0	0	2
314810 Patrocínio	0	2	0	0	2
315150 Piumhi	0	1	0	0	1
315570 Rio Piracicaba	0	1	0	0	1
315670 Sabará	0	1	0	0	1
316090 São Brás do Suaçuí	0	1	0	0	1
316110 São Francisco	0	1	0	0	1
316800 Taiobeiras	0	1	0	0	1
316935 Três Marias	0	1	0	0	1
316990 Ubá	0	1	0	0	1

317010 Uberaba	0	3	0	0	3
317020 Uberlândia	0	9	0	0	9
317100 Vazante	0	1	0	0	1
320080 Baixo Guandu	0	2	0	0	2
320090 Barra de São Francisco	0	3	0	0	3
320130 Cariacica	0	3	0	1	4
320150 Colatina	0	2	0	0	2
320170 Conceição do Castelo	0	1	0	0	1
320320 Linhares	0	4	1	1	6
320350 Montanha	0	2	0	0	2
320405 Pedro Canário	0	1	0	0	1
320425 Ponto Belo	0	1	0	0	1
320500 Serra	0	3	0	0	3
320520 Vila Velha	0	1	0	0	1
320530 Vitória	0	3	0	0	3
330045 Belford Roxo	0	1	0	0	1
330070 Cabo Frio	0	1	0	0	1
330100 Campos dos Goytacazes	0	1	0	0	1
330170 Duque de Caxias	0	1	0	0	1
330240 Macaé	0	7	0	0	7
330320 Nilópolis	0	1	0	0	1
330330 Niterói	0	1	0	0	1
330340 Nova Friburgo	0	1	0	0	1
330350 Nova Iguaçu	0	2	0	0	2
330420 Resende	0	2	0	0	2
330452 Rio das Ostras	0	3	0	0	3
330455 Rio de Janeiro	0	42	0	0	42
330490 São Gonçalo	0	3	0	0	3
350050 Águas de Lindóia	0	1	0	0	1
350210 Andradina	0	1	0	0	1
350270 Apiaí	0	1	0	0	1
350275 Araçariguama	0	1	0	0	1
350280 Araçatuba	0	0	1	0	1
350330 Araras	0	1	0	0	1
350390 Arujá	0	2	0	0	2
350410 Atibaia	0	1	0	0	1
350570 Barueri	0	2	0	0	2
350600 Bauru	0	1	0	0	1
350610 Bebedouro	0	1	0	0	1
350760 Bragança Paulista	0	2	0	0	2
350950 Campinas	0	4	0	0	4
351000 Cândido Mota	0	2	0	0	2
351020 Capão Bonito	0	1	0	0	1
351070 Cardoso	0	1	0	0	1
351300 Cotia	0	1	0	0	1
351380 Diadema	0	1	0	0	1
351490 Elias Fausto	0	1	0	0	1
351500 Embu	0	2	0	0	2
351620 Franca	0	1	0	0	1
351670 Garça	0	1	0	0	1

351840 Guaratinguetá	0	1	0	0	1
351880 Guarulhos	0	5	0	0	5
351905 Holambra	0	2	0	0	2
352044 Ilha Solteira	0	1	0	0	1
352220 Itapecerica da Serra	0	1	0	0	1
352390 Itu	0	1	0	0	1
352590 Jundiaí	0	3	0	0	3
352680 Lençóis Paulista	0	1	0	0	1
352710 Lins	0	1	0	0	1
352900 Marília	0	1	0	0	1
352940 Mauá	0	3	0	0	3
353020 Mirante do Paranapanema	0	1	0	0	1
353050 Mococa	0	2	0	0	2
353070 Mogi Guaçu	0	1	0	0	1
353120 Monte Alegre do Sul	0	1	0	0	1
353150 Monte Azul Paulista	0	2	0	0	2
353260 Nhandeara	0	1	0	0	1
353300 Nova Granada	0	2	0	0	2
353440 Osasco	0	4	0	0	4
353470 Ourinhos	0	1	0	0	1
353550 Paraguaçu Paulista	0	2	0	0	2
353650 Paulínia	0	1	0	0	1
353870 Piracicaba	0	1	0	0	1
353920 Pirapozinho	0	1	0	0	1
354150 Presidente Venceslau	0	1	0	0	1
354325 Ribeirão Grande	0	1	0	0	1
354340 Ribeirão Preto	0	3	0	0	3
354425 Rosana	0	2	0	0	2
354580 Santa Bárbara d'Oeste	0	1	0	0	1
354660 Santa Fé do Sul	0	1	0	0	1
354780 Santo André	0	6	0	0	6
354800 Santo Antônio de Posse	0	1	0	0	1
354850 Santos	0	1	0	0	1
354870 São Bernardo do Campo	0	1	0	0	1
354890 São Carlos	0	1	0	0	1
354910 São João da Boa Vista	0	1	0	0	1
354980 São José do Rio Preto	0	10	0	0	10
354990 São José dos Campos	0	4	0	0	4
355030 São Paulo	0	67	0	1	68
355050 São Pedro do Turvo	0	1	0	0	1
355070 São Sebastião	0	1	0	0	1
355100 São Vicente	0	2	0	0	2
355170 Sertãozinho	0	1	0	0	1
355220 Sorocaba	0	2	0	0	2
355250 Suzano	0	2	0	0	2
355280 Taboão da Serra	0	2	0	0	2
355290 Taciba	0	1	0	0	1
355430 Teodoro Sampaio	0	1	0	0	1
355480 Tremembé	0	1	0	0	1
355540 Ubatuba	0	1	0	0	1

355580 Urânia	0	1	0	0	1
355710 Votuporanga	0	2	0	0	2
410100 Ampére	0	0	1	0	1
410150 Arapongas	0	0	1	0	1
410180 Araucária	0	0	1	0	1
410305 Boa Vista da Aparecida	0	0	2	0	2
410420 Campo Largo	0	0	2	0	2
410480 Cascavel	0	0	1	0	1
410530 Céu Azul	0	0	1	0	1
410540 Chopinzinho	0	0	1	0	1
410630 Corbélia	0	0	1	0	1
410640 Cornélio Procópio	0	0	1	0	1
410690 Curitiba	0	0	4	0	4
410725 Douradina	0	0	1	0	1
410830 Foz do Iguaçu	0	0	4	0	4
410880 Guaíra	0	0	1	0	1
410965 Honório Serpa	0	0	1	0	1
410980 Ibiporã	0	0	1	0	1
411180 Jacarezinho	0	0	1	0	1
411370 Londrina	0	0	3	0	3
411520 Maringá	0	0	7	0	7
411580 Medianeira	0	0	1	0	1
411710 Nova Londrina	0	0	1	0	1
411730 Ortigueira	0	0	1	0	1
411840 Paranavaí	0	0	1	0	1
411940 Piraí do Sul	0	0	1	0	1
412090 Quedas do Iguaçu	0	0	4	0	4
412350 Santa Helena	0	0	1	0	1
412380 Santa Izabel do Oeste	0	0	1	0	1
412550 São José dos Pinhais	0	0	4	0	4
412625 Sarandi	0	0	1	0	1
412770 Toledo	0	0	1	0	1
412785 Três Barras do Paraná	0	0	3	0	3
412855 Vera Cruz do Oeste	0	0	1	0	1
420050 Águas de Chapecó	0	0	3	0	3
420055 Águas Frias	0	0	1	0	1
420240 Blumenau	0	0	4	0	4
420310 Caibi	0	0	1	0	1
420320 Camboriú	0	0	1	0	1
420390 Capinzal	0	0	1	0	1
420420 Chapecó	0	0	2	2	4
420430 Concórdia	0	0	3	0	3
420540 Florianópolis	0	0	1	0	1
420550 Fraiburgo	0	0	1	0	1
420700 Içara	0	0	1	0	1
420830 Itapema	0	0	1	0	1
420910 Joinville	0	0	1	0	1
421050 Maravilha	0	0	2	0	2
421130 Navegantes	0	0	1	0	1
421170 Orleans	0	0	1	0	1

421210 Palmitos	0	0	1	0	1
421500 Rio Negrinho	0	0	1	0	1
421580 São Bento do Sul	0	0	1	0	1
421660 São José	0	0	1	0	1
421870 Tubarão	0	0	3	0	3
430160 Bagé	0	0	1	0	1
430460 Canoas	0	0	1	0	1
430510 Caxias do Sul	0	0	1	0	1
430920 Gravataí	0	0	1	0	1
431020 Ijuí	0	0	1	0	1
431115 Jóia	0	0	1	0	1
431230 Miraguaí	0	0	1	0	1
431340 Novo Hamburgo	0	0	1	0	1
431410 Passo Fundo	0	0	1	0	1
431480 Portão	0	0	3	0	3
431490 Porto Alegre	0	0	3	0	3
431500 Porto Lucena	0	0	1	0	1
431690 Santa Maria	0	0	1	0	1
431780 Santo Augusto	0	0	1	0	1
432010 Sarandi	0	0	1	0	1
432150 Torres	0	0	1	0	1
432160 Tramandaí	0	0	1	0	1
500270 Campo Grande	0	0	0	13	13
500370 Dourados	0	0	0	2	2
500769 São Gabriel do Oeste	0	0	0	2	2
500793 Sonora	0	0	0	1	1
500830 Três Lagoas	0	0	0	3	3
510025 Alta Floresta	1	0	0	0	1
520025 Águas Lindas de Goiás	0	0	0	1	1
520110 Anápolis	0	0	0	5	5
520140 Aparecida de Goiânia	0	0	0	8	8
520350 Bom Jesus de Goiás	0	0	0	1	1
520440 Caiapônia	0	0	0	2	2
520450 Caldas Novas	0	0	0	2	2
520870 Goiânia	0	1	0	38	39
520880 Goianira	0	0	0	1	1
520890 Goiás	0	0	0	1	1
521020 Iporá	0	0	0	1	1
521180 Jaraguá	0	0	0	1	1
521190 Jataí	0	0	0	2	2
521250 Luziânia	0	0	0	8	8
521308 Minaçu	0	0	0	2	2
521450 Nerópolis	0	0	0	1	1
521800 Porangatu	0	0	0	3	3
521860 Rialma	0	0	0	1	1
521880 Rio Verde	0	0	0	2	2
521930 Santa Helena de Goiás	0	0	0	1	1
522045 Senador Canedo	0	0	0	1	1
522140 Trindade	0	0	0	2	2
522185 Valparaíso de Goiás	0	0	0	1	1

530010 Brasília	1	1	0	44	46
Total	172	390	115	158	835

MALÁRIA - Casos confirmados Notificados no Sistema de Informação de Agravos de Notificação - Sinan Net

Casos confirmados por Munic. Residência e result.parasitológi

Autoctone Mun Res: Não

Período:2011

Munic. Residência	Falciparum	F+FG	Vivax	F+V	Total
110080 Candeias do Jamari	0	1	0	0	1
110012 Ji-Paraná	0	0	1	0	1
110013 Machadinho D'Oeste	0	0	1	0	1
110140 Monte Negro	0	1	0	0	1
110033 Nova Mamoré	0	1	0	0	1
110020 Porto Velho	1	3	0	2	6
110025 Presidente Médici	0	1	0	0	1
130014 Apuí	0	1	0	0	1
130260 Manaus	0	1	1	0	2
140010 Boa Vista	0	1	0	1	2
150360 Itaituba	0	1	1	0	2
150680 Santarém	0	0	0	1	1
160030 Macapá	0	1	0	1	2
170200 Araguaçu	0	0	0	2	2
210390 Duque Bacelar	1	0	0	0	1
210570 Lago da Pedra	2	0	0	0	2
210780 Parnarama	1	0	0	0	1
211000 Santa Luzia	1	0	0	0	1
211130 São Luís	0	1	0	0	1
211220 Timon	1	0	0	0	1
211300 Vitorino Freire	1	0	0	0	1
211400 Zé Doca	0	0	1	0	1
220025 Alagoinha do Piauí	1	0	0	0	1
220050 Amarante	1	0	0	0	1
220120 Barras	4	0	0	0	4
220130 Barreiras do Piauí	1	0	0	0	1
220150 Batalha	2	0	0	0	2
220217 Campo Largo do Piauí	1	0	0	0	1
220390 Floriano	1	0	0	0	1
220420 Francisco Santos	1	0	0	0	1
220450 Guadalupe	1	0	0	0	1
220545 Joca Marques	3	0	0	0	3
220580 Luzilândia	15	0	0	0	15
220585 Madeiro	1	0	0	0	1
220610 Matias Olímpio	5	0	0	0	5
220620 Miguel Alves	1	0	0	0	1
220640 Monsenhor Gil	3	0	0	0	3
220840 Piripiri	8	0	0	0	8
220850 Porto	3	0	0	0	3
220880 Regeneração	1	0	0	0	1
220920 Santa Filomena	1	0	0	0	1
220930 Santa Luz	1	0	0	0	1
221090 Socorro do Piauí	1	0	0	0	1

221100 Teresina	8	0	0	0	8
221120 Uruçuí	6	0	0	0	6
230370 Caucaia	2	0	0	0	2
230380 Cedro	2	0	0	0	2
230410 Crateús	1	0	0	0	1
230425 Cruz	1	0	0	0	1
230440 Fortaleza	14	1	0	0	15
230630 Itapagé	1	0	0	0	1
230730 Juazeiro do Norte	1	0	0	0	1
230870 Morada Nova	1	0	0	0	1
231350 Trairi	2	0	0	0	2
231390 Uruoca	1	0	0	0	1
231410 Viçosa do Ceará	1	0	0	0	1
240080 Angicos	2	0	0	0	2
240310 Currais Novos	1	0	0	0	1
240810 Natal	1	0	0	0	1
240850 Ouro Branco	2	0	0	0	2
241120 Santa Cruz	1	0	0	0	1
241200 São Gonçalo do Amarante	4	0	0	0	4
250250 Boqueirão	1	0	0	0	1
250750 João Pessoa	2	0	0	0	2
251010 Nova Floresta	1	0	0	0	1
251080 Patos	2	0	0	2	4
260005 Abreu e Lima	1	0	0	0	1
260110 Araripina	2	0	0	0	2
260200 Bodocó	1	0	0	0	1
260300 Cabrobó	1	0	0	0	1
260790 Jaboatão dos Guararapes	3	0	0	0	3
260960 Olinda	1	0	0	0	1
261070 Paulista	2	0	0	0	2
261110 Petrolina	1	0	0	0	1
261160 Recife	6	0	0	0	6
261640 Vitória de Santo Antão	1	0	0	0	1
270240 Delmiro Gouveia	3	0	0	0	3
270430 Maceió	2	0	0	0	2
280020 Aquidabã	1	0	0	0	1
280030 Aracaju	2	0	0	0	2
280120 Canindé de São Francisco	1	0	0	0	1
280260 Gracho Cardoso	1	0	0	0	1
280380 Malhada dos Bois	1	0	0	0	1
280540 Poço Redondo	1	0	0	0	1
290320 Barreiras	0	0	0	1	1
290650 Candeias	2	0	0	0	2
290930 Correntina	2	0	0	0	2
291160 Governador Mangabeira	1	0	0	0	1
291465 Itabela	2	0	0	0	2
291630 Itapebi	1	0	0	0	1
291840 Juazeiro	1	0	0	0	1
291955 Luís Eduardo Magalhães	1	0	0	0	1
292090 Mascote	2	0	0	0	2

292400 Paulo Afonso	1	0	0	0	1
292740 Salvador	2	0	0	0	2
292840 Santa Rita de Cássia	1	0	0	0	1
293150 Teofilândia	1	0	0	0	1
310350 Araguari	0	4	0	0	4
310450 Arinos	0	7	0	0	7
310490 Baependi	0	1	0	0	1
310560 Barbacena	0	1	0	0	1
310620 Belo Horizonte	0	27	0	0	27
310670 Betim	0	5	0	0	5
311090 Campanha	0	1	0	0	1
311160 Campos Gerais	0	1	0	0	1
311320 Carandaí	0	1	0	0	1
311570 Central de Minas	0	1	0	0	1
311860 Contagem	0	3	0	0	3
311930 Coromandel	0	1	0	0	1
311940 Coronel Fabriciano	0	1	0	0	1
312230 Divinópolis	0	1	0	0	1
312770 Governador Valadares	0	1	0	0	1
312930 Iapu	0	1	0	0	1
313010 Igarapé	0	1	0	0	1
313130 Ipatinga	0	2	0	0	2
313170 Itabira	0	1	0	0	1
313190 Itabirito	0	1	0	0	1
313375 Itaú de Minas	0	3	0	0	3
313480 Jacuí	0	1	0	0	1
313510 Janaúba	0	1	0	0	1
313670 Juiz de Fora	0	3	0	0	3
313720 Lagoa da Prata	0	2	0	0	2
313900 Machado	0	1	0	0	1
313970 Maravilhas	0	1	0	0	1
314000 Mariana	0	1	0	0	1
314050 Martinho Campos	0	1	0	0	1
314480 Nova Lima	0	1	0	0	1
314710 Pará de Minas	0	1	0	0	1
314700 Paracatu	0	1	0	0	1
314790 Passos	0	1	0	0	1
314800 Patos de Minas	0	1	0	0	1
314810 Patrocínio	0	4	0	0	4
315250 Pouso Alegre	0	1	0	0	1
315580 Rio Pomba	0	1	0	0	1
315670 Sabará	0	4	0	0	4
315780 Santa Luzia	0	1	0	0	1
315820 Santa Maria do Suaçuí	0	1	0	0	1
315980 Santa Vitória	0	1	0	0	1
316130 São Francisco de Sales	0	3	0	0	3
316257 São João do Manteninha	0	1	0	0	1
316350 São José do Jacuri	0	1	0	0	1
317010 Uberaba	0	2	0	0	2
317020 Uberlândia	0	2	0	0	2

317120 Vespasiano	0	1	0	0	1
317130 Viçosa	0	1	0	0	1
320013 Águia Branca	0	1	0	0	1
320060 Aracruz	0	1	0	0	1
320080 Baixo Guandu	0	2	0	0	2
320090 Barra de São Francisco	0	2	0	0	2
320120 Cachoeiro de Itapemirim	0	1	0	0	1
320130 Cariacica	0	4	0	0	4
320150 Colatina	0	2	0	0	2
320225 Governador Lindenberg	0	1	0	0	1
320240 Guarapari	0	1	0	0	1
320320 Linhares	0	6	1	0	7
320334 Marechal Floriano	0	1	0	0	1
320390 Nova Venécia	0	3	0	0	3
320490 São Mateus	0	2	0	0	2
320500 Serra	0	3	0	0	3
320517 Vila Valério	0	1	0	0	1
320520 Vila Velha	0	3	0	0	3
320530 Vitória	0	3	0	0	3
330100 Campos dos Goytacazes	0	2	0	0	2
330140 Conceição de Macabu	0	1	0	0	1
330150 Cordeiro	0	1	0	0	1
330170 Duque de Caxias	0	2	0	0	2
330200 Itaguaí	0	1	0	0	1
330240 Macaé	0	1	0	0	1
330260 Mangaratiba	0	1	0	0	1
330270 Maricá	0	1	0	0	1
330330 Niterói	0	1	0	0	1
330350 Nova Iguaçu	0	2	0	0	2
330390 Petrópolis	0	2	0	0	2
330452 Rio das Ostras	0	1	0	0	1
330455 Rio de Janeiro	0	63	0	0	63
330490 São Gonçalo	0	2	0	0	2
330540 Sapucaia	0	1	0	0	1
330630 Volta Redonda	0	1	0	0	1
350260 Aparecida d'Oeste	0	1	0	0	1
350400 Assis	0	1	0	0	1
350550 Barretos	0	1	0	0	1
350560 Barrinha	0	1	0	0	1
350570 Barueri	0	1	0	0	1
350590 Batatais	0	1	0	0	1
350610 Bebedouro	0	2	0	0	2
350750 Botucatu	0	1	0	0	1
350760 Bragança Paulista	0	1	0	0	1
350840 Cabreúva	0	1	0	0	1
350900 Caieiras	0	1	0	0	1
350950 Campinas	0	6	0	0	6
350970 Campos do Jordão	0	1	0	0	1
351000 Cândido Mota	0	1	0	0	1
351300 Cotia	0	1	0	0	1

351380 Diadema	0	1	0	0	1
351500 Embu	0	2	0	0	2
351620 Franca	0	3	0	0	3
351630 Francisco Morato	0	1	0	0	1
351870 Guarujá	0	1	0	0	1
351880 Guarulhos	0	9	0	0	9
351890 Guzolândia	0	1	0	0	1
351907 Hortolândia	0	1	0	0	1
352050 Indaiatuba	0	3	0	0	3
352100 Iperó	0	1	0	0	1
352230 Itapetininga	0	1	0	0	1
352240 Itapeva	0	1	0	0	1
352250 Itapevi	0	1	0	0	1
352310 Itaquaquecetuba	0	1	0	0	1
352460 Jacupiranga	1	0	0	0	1
352470 Jaguariúna	0	1	0	0	1
352480 Jales	0	1	0	0	1
352500 Jandira	0	1	0	0	1
352590 Jundiaí	0	4	0	0	4
352680 Lençóis Paulista	0	1	0	0	1
352690 Limeira	0	1	0	0	1
352710 Lins	0	1	0	0	1
352900 Marília	0	3	0	0	3
353060 Mogi das Cruzes	0	1	0	0	1
353070 Mogi Guaçu	0	2	0	0	2
353380 Óleo	0	1	0	0	1
353390 Olímpia	0	1	0	0	1
353400 Onda Verde	0	1	0	0	1
353440 Osasco	0	4	0	0	4
353470 Ourinhos	0	1	1	0	2
353810 Pindorama	0	1	0	0	1
353920 Pirapozinho	0	1	0	0	1
354070 Porto Ferreira	0	1	0	0	1
354130 Presidente Epitácio	0	1	0	0	1
354140 Presidente Prudente	0	6	0	0	6
354150 Presidente Venceslau	0	1	0	0	1
354260 Registro	0	1	0	0	1
354330 Ribeirão Pires	0	1	0	0	1
354340 Ribeirão Preto	0	5	0	0	5
354410 Rio Grande da Serra	0	1	0	0	1
354425 Rosana	0	3	0	0	3
354530 Salto de Pirapora	0	1	0	0	1
354730 Santana de Parnaíba	0	1	0	0	1
354770 Santo Anastácio	0	1	0	0	1
354780 Santo André	0	1	0	0	1
354850 Santos	0	2	0	0	2
354870 São Bernardo do Campo	0	7	0	0	7
354890 São Carlos	0	1	0	0	1
354980 São José do Rio Preto	0	2	0	0	2
354990 São José dos Campos	0	1	0	0	1

355030 São Paulo	0	49	0	0	49
355170 Sertãozinho	0	1	0	0	1
355220 Sorocaba	0	2	0	0	2
355250 Suzano	0	4	0	0	4
355500 Tupã	0	1	0	0	1
355580 Urânia	0	1	0	0	1
355620 Valinhos	0	1	0	0	1
355670 Vinhedo	0	2	0	0	2
355700 Votorantim	0	1	0	0	1
410180 Araucária	0	0	2	0	2
410370 Cambé	0	0	1	0	1
410400 Campina Grande do Sul	0	0	1	0	1
410420 Campo Largo	0	0	1	0	1
410430 Campo Mourão	0	0	1	0	1
410450 Capanema	0	0	1	0	1
410480 Cascavel	0	0	4	0	4
410580 Colombo	0	0	3	0	3
410630 Corbélia	0	0	4	0	4
410690 Curitiba	0	0	7	0	7
410710 Diamante do Norte	0	0	1	0	1
410830 Foz do Iguaçu	0	0	8	0	8
410880 Guaíra	0	0	2	0	2
411100 Itambaracá	0	0	1	0	1
411130 Itaúna do Sul	0	0	1	0	1
411260 Jardim Olinda	0	0	1	0	1
411370 Londrina	0	0	5	0	5
411520 Maringá	0	0	6	0	6
411605 Missal	0	0	1	0	1
411710 Nova Londrina	0	0	1	0	1
411820 Paranaguá	0	0	1	0	1
411840 Paranavaí	0	0	1	0	1
411850 Pato Branco	0	0	2	0	2
411950 Piraquara	0	0	2	0	2
411990 Ponta Grossa	0	0	2	0	2
412000 Porecatu	0	0	1	0	1
412215 Rio Bonito do Iguaçu	0	0	1	0	1
412410 Santo Antônio da Platina	0	0	2	0	2
412520 São Jorge d'Oeste	0	0	1	0	1
412550 São José dos Pinhais	0	0	3	0	3
412770 Toledo	0	0	2	0	2
420400 Catanduvas	0	0	1	0	1
420430 Concórdia	0	0	2	0	2
420540 Florianópolis	0	0	6	0	6
420545 Forquilhinha	0	0	1	0	1
420550 Fraiburgo	0	0	1	0	1
420765 Iporã do Oeste	1	0	0	0	1
420820 Itajaí	0	0	1	0	1
420910 Joinville	0	0	5	0	5
420930 Lages	0	0	1	0	1
421420 Quilombo	0	0	1	0	1

421690 São Lourenço do Oeste	0	0	1	0	1
421830 Três Barras	0	0	1	0	1
430300 Cachoeira do Sul	0	0	3	0	3
430440 Canela	0	0	1	0	1
430460 Canoas	0	0	1	0	1
430610 Cruz Alta	0	0	3	0	3
430790 Farroupilha	0	1	0	0	1
431240 Montenegro	0	0	2	0	2
431270 Nonoai	0	0	1	0	1
431350 Osório	0	0	1	0	1
431410 Passo Fundo	0	0	1	0	1
431490 Porto Alegre	0	0	1	0	1
432110 Tapes	0	0	1	0	1
500270 Campo Grande	0	0	0	11	11
500295 Chapadão do Sul	0	0	0	1	1
500325 Costa Rica	0	0	0	1	1
500330 Coxim	0	0	0	2	2
500370 Dourados	0	0	0	2	2
500720 Rio Brilhante	0	0	0	1	1
500793 Sonora	0	0	0	2	2
500830 Três Lagoas	0	0	0	1	1
510558 Marcelândia	0	0	1	0	1
520025 Águas Lindas de Goiás	0	0	0	1	1
520110 Anápolis	0	0	0	2	2
520140 Aparecida de Goiânia	0	0	0	6	6
520150 Aporé	0	0	0	1	1
520425 Cachoeira Dourada	0	0	0	1	1
520440 Caiapônia	0	0	0	1	1
520450 Caldas Novas	0	0	0	1	1
520551 Cocalzinho de Goiás	0	0	0	1	1
520870 Goiânia	0	0	0	20	20
520910 Goiatuba	0	0	0	3	3
520995 Indiara	0	0	0	1	1
521180 Jaraguá	0	0	0	1	1
521250 Luziânia	0	0	0	1	1
521308 Minaçu	0	0	0	3	3
521486 Nova Glória	0	0	0	1	1
521800 Porangatu	0	0	0	1	1
521850 Quirinópolis	0	0	0	1	1
521880 Rio Verde	0	0	0	2	2
521930 Santa Helena de Goiás	0	0	0	1	1
521975 Santo Antônio do Descoberto	0	0	0	1	1
522040 São Simão	0	0	0	1	1
522045 Senador Canedo	0	0	0	1	1
522185 Valparaíso de Goiás	0	0	0	2	2
530010 Brasília	0	0	0	30	30
Total	172	413	114	115	814

MALÁRIA - Casos confirmados Notificados no Sistema de Informação de Agravos de Notificação - Sinan Net
Casos confirmados por Munic. Residência e result.parasitológi
Autoctone Mun Res: Não
Período:2012

Munic. Residência	Falciparum	F+FG	Vivax	F+V	Total
110001 Alta Floresta D'Oeste	0	0	1	0	1
110002 Ariquemes	0	1	0	0	1
110004 Cacoal	0	0	1	1	2
110012 Ji-Paraná	0	1	0	0	1
110020 Porto Velho	0	2	1	0	3
130260 Manaus	1	1	0	0	2
150360 Itaituba	0	2	0	0	2
150420 Marabá	0	1	0	0	1
160005 Serra do Navio	0	0	0	1	1
210043 Alto Alegre do Maranhão	1	0	0	0	1
210095 Arame	0	1	0	0	1
210150 Barão de Grajaú	1	0	0	0	1
211130 São Luís	1	0	0	0	1
220050 Amarante	2	0	0	0	2
220120 Barras	6	0	0	0	6
220194 Boqueirão do Piauí	1	0	0	0	1
220196 Brasileira	1	0	0	0	1
220205 Cabeceiras do Piauí	1	0	0	0	1
220217 Campo Largo do Piauí	2	0	0	0	2
220370 Esperantina	1	0	0	0	1
220390 Floriano	1	0	0	0	1
220430 Fronteiras	1	0	0	0	1
220435 Geminiano	1	0	0	0	1
220580 Luzilândia	6	0	0	0	6
220610 Matias Olímpio	7	0	0	0	7
220840 Piripiri	5	0	0	0	5
220880 Regeneração	1	0	0	0	1
220980 São Gonçalo do Piauí	4	0	0	0	4
221100 Teresina	11	0	0	0	11
221130 Valença do Piauí	1	0	0	0	1
221160 Vila Nova do Piauí	1	0	0	0	1
230425 Cruz	1	0	0	0	1
230440 Fortaleza	12	0	0	0	12
230630 Itapagé	1	0	0	0	1
230640 Itapipoca	1	0	0	0	1
230690 Jaguaribe	0	1	0	0	1
231020 Paracuru	1	0	0	0	1
231030 Parambu	1	0	0	0	1
231310 Tabuleiro do Norte	3	0	0	0	3
240100 Apodi	1	0	0	0	1
240200 Caicó	3	0	0	0	3
240310 Currais Novos	1	0	0	0	1
240560 Jardim de Piranhas	1	0	0	0	1
240710 Macaíba	2	0	0	0	2

240810 Natal	7	0	0	0	7
241240 São José do Seridó	1	0	0	0	1
250370 Cajazeiras	1	0	0	0	1
250375 Cajazeirinhas	1	0	0	0	1
250460 Conde	1	0	0	0	1
250750 João Pessoa	1	1	0	0	2
251080 Patos	3	0	0	0	3
251390 São Bento	1	0	0	0	1
251450 São José de Piranhas	1	0	0	0	1
260720 Ipojuca	1	0	0	0	1
260790 Jaboatão dos Guararapes	3	0	0	0	3
260875 Lagoa Grande	0	1	0	0	1
261110 Petrolina	1	0	0	0	1
261160 Recife	2	0	0	0	2
270010 Água Branca	1	0	0	0	1
270030 Arapiraca	1	1	0	0	2
270210 Colônia Leopoldina	0	1	0	0	1
270240 Delmiro Gouveia	1	0	0	0	1
270430 Maceió	2	0	0	0	2
270570 Olho d'Água das Flores	1	0	0	0	1
280030 Aracaju	1	0	0	0	1
280210 Estância	1	0	0	0	1
280390 Malhador	1	0	0	0	1
280530 Pirambu	1	0	0	0	1
280560 Porto da Folha	1	0	0	0	1
280590 Riachuelo	1	0	0	0	1
290340 Belmonte	1	0	0	0	1
290570 Camaçari	1	0	0	0	1
291080 Feira de Santana	1	0	0	0	1
291530 Itagimirim	1	0	0	0	1
292400 Paulo Afonso	1	0	0	0	1
292740 Salvador	2	0	0	0	2
293015 Serra do Ramalho	1	0	0	0	1
293050 Serrinha	1	0	0	0	1
293070 Simões Filho	3	0	0	0	3
310110 Aimorés	0	2	0	0	2
310150 Além Paraíba	0	1	0	0	1
310160 Alfenas	0	1	0	0	1
310350 Araguari	0	3	0	0	3
310420 Arcos	0	1	0	0	1
310620 Belo Horizonte	0	23	0	0	23
310670 Betim	0	4	0	0	4
310690 Bicas	0	1	0	0	1
310740 Bom Despacho	0	2	0	0	2
311050 Camanducaia	0	2	0	0	2
311800 Congonhas	0	1	0	0	1
311860 Contagem	0	5	0	0	5
311930 Coromandel	0	1	0	0	1
311940 Coronel Fabriciano	0	2	0	0	2
312160 Diamantina	0	1	0	0	1

312230 Divinópolis	0	2	0	0	2
312595 Fervedouro	0	1	0	0	1
312730 Galiléia	0	1	0	0	1
312770 Governador Valadares	0	2	0	0	2
312820 Guaraciaba	0	1	0	0	1
313115 Ipaba	0	1	0	0	1
313130 Ipatinga	0	1	0	0	1
313180 Itabirinha	0	1	0	0	1
313190 Itabirito	0	1	0	0	1
313300 Itamonte	0	2	0	0	2
313670 Juiz de Fora	0	2	0	0	2
313960 Mantena	0	1	0	0	1
313970 Maravilhas	0	1	0	0	1
314310 Monte Carmelo	0	1	0	0	1
314330 Montes Claros	0	1	0	0	1
314650 Pains	0	1	0	0	1
314710 Pará de Minas	0	1	0	0	1
314810 Patrocínio	0	1	0	0	1
315250 Pouso Alegre	0	2	0	0	2
315300 Pratinha	0	1	0	0	1
315460 Ribeirão das Neves	0	2	0	0	2
315780 Santa Luzia	0	3	0	0	3
316190 São Gonçalo do Rio Abaixo	0	1	0	0	1
316210 São Gotardo	0	1	0	0	1
316290 São João Nepomuceno	0	1	0	0	1
316870 Timóteo	0	1	0	0	1
317020 Uberlândia	0	7	0	0	7
317120 Vespasiano	0	1	0	0	1
320040 Anchieta	0	1	0	0	1
320130 Cariacica	0	3	0	0	3
320140 Castelo	0	1	0	0	1
320150 Colatina	0	3	0	0	3
320250 Ibiraçu	0	1	0	0	1
320320 Linhares	0	1	0	0	1
320400 Pancas	0	1	0	0	1
320425 Ponto Belo	0	2	0	0	2
320460 Santa Teresa	0	1	0	0	1
320500 Serra	0	1	0	0	1
320520 Vila Velha	0	6	0	0	6
320530 Vitória	0	3	0	0	3
330010 Angra dos Reis	0	2	0	0	2
330170 Duque de Caxias	0	6	0	0	6
330225 Itatiaia	0	1	0	0	1
330240 Macaé	0	4	0	0	4
330250 Magé	0	1	0	0	1
330270 Maricá	0	1	0	0	1
330330 Niterói	0	9	0	0	9
330360 Paracambi	0	2	0	0	2
330420 Resende	0	2	0	0	2
330455 Rio de Janeiro	0	87	0	0	87

330490 São Gonçalo	0	1	0	0	1
330510 São João de Meriti	0	1	0	0	1
330610 Valença	0	1	0	0	1
350160 Americana	0	1	0	0	1
350270 Apiaí	0	1	0	0	1
350280 Araçatuba	0	1	0	0	1
350320 Araraquara	0	1	0	0	1
350400 Assis	0	1	0	0	1
350410 Atibaia	0	2	0	0	2
350570 Barueri	0	1	0	0	1
350590 Batatais	0	1	0	0	1
350600 Bauru	0	3	0	0	3
350635 Bertioga	0	1	0	0	1
350850 Caçapava	0	1	0	0	1
350920 Cajamar	0	1	0	0	1
350950 Campinas	0	6	0	0	6
350960 Campo Limpo Paulista	0	1	0	0	1
351000 Cândido Mota	0	2	0	0	2
351060 Carapicuíba	0	1	0	0	1
351300 Cotia	0	1	0	0	1
351350 Cubatão	0	4	0	0	4
351440 Dracena	0	1	0	0	1
351620 Franca	0	2	0	0	2
351840 Guaratinguetá	0	1	0	0	1
351870 Guarujá	0	3	0	0	3
351880 Guarulhos	0	4	0	0	4
351907 Hortolândia	0	2	0	0	2
352440 Jacareí	0	2	0	0	2
352470 Jaguariúna	0	2	0	0	2
352590 Jundiaí	0	3	0	0	3
352690 Limeira	0	3	0	0	3
352770 Luiziânia	0	1	0	1	2
353060 Mogi das Cruzes	0	2	0	0	2
353300 Nova Granada	0	1	0	0	1
353390 Olímpia	0	1	0	0	1
353440 Osasco	0	1	0	0	1
353780 Piedade	0	2	0	0	2
353800 Pindamonhangaba	0	1	0	0	1
353870 Piracicaba	0	1	0	0	1
354020 Pontal	0	1	0	0	1
354060 Porto Feliz	0	1	0	0	1
354100 Praia Grande	0	1	0	0	1
354140 Presidente Prudente	0	5	0	0	5
354260 Registro	0	1	0	0	1
354340 Ribeirão Preto	0	1	0	0	1
354580 Santa Bárbara d'Oeste	0	1	0	0	1
354730 Santana de Parnaíba	0	1	0	0	1
354780 Santo André	0	3	0	0	3
354850 Santos	0	5	0	0	5
354870 São Bernardo do Campo	1	1	0	0	2

354890 São Carlos	0	3	0	0	3
354980 São José do Rio Preto	0	5	0	0	5
354990 São José dos Campos	0	9	0	0	9
355030 São Paulo	0	38	0	0	38
355060 São Roque	0	1	0	0	1
355100 São Vicente	0	4	0	0	4
355220 Sorocaba	0	2	0	0	2
355280 Taboão da Serra	0	2	0	0	2
355410 Taubaté	0	1	0	0	1
355580 Urânia	0	2	0	0	2
355700 Votorantim	0	1	0	0	1
410120 Antonina	0	0	2	0	2
410180 Araucária	0	0	1	0	1
410300 Boa Esperança	0	0	1	0	1
410460 Capitão Leônidas Marques	0	0	1	0	1
410480 Cascavel	0	0	2	0	2
410510 Centenário do Sul	0	0	2	0	2
410540 Chopinzinho	0	0	2	0	2
410630 Corbélia	0	0	1	0	1
410690 Curitiba	0	0	5	0	5
410830 Foz do Iguaçu	0	0	3	0	3
410930 Guaraniaçu	0	0	1	0	1
411330 Laranjeiras do Sul	0	0	1	0	1
411370 Londrina	0	1	4	0	5
411390 Mallet	0	0	1	0	1
411460 Marechal Cândido Rondon	0	0	2	0	2
411480 Marialva	0	0	1	0	1
411520 Maringá	0	0	3	0	3
411580 Medianeira	0	0	1	0	1
411585 Mercedes	0	0	1	0	1
411605 Missal	0	0	1	0	1
411950 Piraquara	0	0	2	0	2
411990 Ponta Grossa	0	0	1	0	1
412060 Prudentópolis	0	0	4	0	4
412440 Santo Antônio do Sudoeste	0	0	1	0	1
412570 São Miguel do Iguaçu	0	0	1	0	1
412710 Telêmaco Borba	0	0	1	0	1
412770 Toledo	0	0	2	0	2
412790 Tuneiras do Oeste	0	0	1	0	1
420210 Barra Velha	0	0	2	0	2
420240 Blumenau	0	0	2	0	2
420280 Braço do Norte	0	0	1	0	1
420290 Brusque	0	0	1	0	1
420300 Caçador	0	0	1	0	1
420320 Camboriú	0	0	2	0	2
420415 Celso Ramos	0	0	1	0	1
420420 Chapecó	0	0	1	0	1
420460 Criciúma	0	0	1	0	1
420540 Florianópolis	0	0	7	0	7
420550 Fraiburgo	0	0	1	0	1

420590 Gaspar	0	0	1	0	1
420660 Guarujá do Sul	0	1	2	0	3
420740 Imbuia	0	0	1	0	1
420890 Jaraguá do Sul	0	0	1	0	1
420900 Joaçaba	0	0	1	0	1
420910 Joinville	0	0	2	0	2
421050 Maravilha	0	0	1	0	1
421130 Navegantes	0	0	1	0	1
421210 Palmitos	0	0	1	0	1
421320 Pomerode	0	0	1	0	1
421440 Rio das Antas	0	0	1	0	1
421630 São João Batista	0	0	2	0	2
421720 São Miguel do Oeste	0	0	1	0	1
430230 Bom Jesus	0	0	1	0	1
430480 Carlos Barbosa	0	0	1	0	1
430510 Caxias do Sul	0	0	1	0	1
431290 Nova Bassano	0	0	1	0	1
431330 Nova Prata	0	0	1	0	1
431410 Passo Fundo	0	0	1	0	1
431490 Porto Alegre	0	0	5	0	5
432180 Três de Maio	0	0	1	0	1
432230 Tuparendi	0	0	1	0	1
500060 Amambai	0	0	0	1	1
500260 Camapuã	0	0	0	1	1
500270 Campo Grande	0	1	0	10	11
500295 Chapadão do Sul	0	0	0	1	1
500325 Costa Rica	0	0	0	1	1
500460 Itaquiraí	0	0	0	1	1
500515 Juti	0	0	0	1	1
500570 Naviraí	0	0	0	2	2
500769 São Gabriel do Oeste	0	0	0	1	1
500793 Sonora	0	0	0	1	1
500795 Tacuru	0	0	0	1	1
510340 Cuiabá	0	0	1	0	1
510840 Várzea Grande	0	1	0	0	1
520110 Anápolis	0	1	0	5	6
520140 Aparecida de Goiânia	0	0	0	5	5
520150 Aporé	0	0	0	1	1
520320 Barro Alto	0	0	0	1	1
520380 Britânia	0	0	0	1	1
520410 Cachoeira Alta	0	0	0	1	1
520450 Caldas Novas	0	0	0	1	1
520545 Cezarina	0	0	0	1	1
520549 Cidade Ocidental	0	0	0	1	1
520620 Cristalina	0	0	0	1	1
520870 Goiânia	0	0	0	28	28
520890 Goiás	0	0	0	1	1
521000 Inhumas	0	0	0	1	1
521220 Jussara	0	0	0	1	1
521250 Luziânia	0	0	0	1	1

521308 Minaçu	0	0	0	7	7
521380 Morrinhos	0	0	0	2	2
521460 Niquelândia	0	0	0	3	3
521570 Palmeiras de Goiás	0	0	0	2	2
521880 Rio Verde	0	0	0	3	3
522020 São Miguel do Araguaia	0	0	0	1	1
522140 Trindade	0	0	0	1	1
522150 Turvânia	0	0	0	1	1
522185 Valparaíso de Goiás	0	0	0	1	1
530010 Brasília	0	2	0	20	22
Total	134	407	102	115	758

MALÁRIA - Casos confirmados Notificados no Sistema de Informação de Agravos de Notificação - Sinan Net

Casos confirmados por Munic. Residência e result.parasitológi
Autoctone Mun Res: Não
Período:2013

Munic. Residência	Falciparum	F+FG	Vivax	F+V	Total
110080 Candeias do Jamari	0	1	1	0	2
110012 Ji-Paraná	0	1	1	0	2
110020 Porto Velho	0	2	0	0	2
110028 Rolim de Moura	0	0	1	0	1
120042 Rodrigues Alves	0	0	0	1	1
130260 Manaus	0	1	0	0	1
140010 Boa Vista	1	1	0	0	2
150140 Belém	0	1	0	0	1
150360 Itaituba	0	1	0	0	1
160020 Calçoene	0	2	0	0	2
160030 Macapá	0	1	0	0	1
172100 Palmas	1	0	0	0	1
210005 Açailândia	0	1	0	0	1
210160 Barra do Corda	1	0	0	0	1
210220 Buriti	1	0	0	0	1
210570 Lago da Pedra	1	0	0	0	1
210596 Lagoa Grande do Maranhão	1	0	0	0	1
211220 Timon	1	0	0	0	1
211300 Vitorino Freire	1	0	0	0	1
210000 Município ignorado - MA	0	0	0	1	1
220140 Barro Duro	1	0	0	0	1
220196 Brasileira	1	0	0	0	1
220230 Canto do Buriti	2	0	0	0	2
220450 Guadalupe	1	0	0	0	1
220540 Joaquim Pires	1	0	0	0	1
220545 Joca Marques	2	0	0	0	2
220550 José de Freitas	1	0	0	0	1
220580 Luzilândia	5	0	0	0	5
220610 Matias Olímpio	3	0	0	0	3
220750 Palmeirais	2	0	0	0	2
220770 Parnaíba	1	0	0	0	1
220840 Piripiri	2	0	0	0	2
220850 Porto	1	0	0	0	1
220880 Regeneração	3	0	0	0	3
220980 São Gonçalo do Piauí	1	0	0	0	1
221100 Teresina	20	0	0	0	20
221140 Várzea Grande	1	0	0	0	1
230075 Amontada	2	0	0	0	2
230110 Aracati	1	0	0	0	1
230370 Caucaia	3	0	0	0	3
230440 Fortaleza	8	0	0	0	8
230523 Horizonte	1	0	0	0	1
230730 Juazeiro do Norte	1	0	0	0	1

231126 Quiterianópolis	2	0	0	0	2
231140 Quixeramobim	1	0	0	0	1
240200 Caicó	1	0	0	0	1
240810 Natal	2	0	0	0	2
240325 Parnamirim	2	0	0	0	2
250750 João Pessoa	2	0	0	0	2
251080 Patos	1	0	0	0	1
251650 Taperoá	1	0	0	0	1
260790 Jaboatão dos Guararapes	2	0	0	0	2
260950 Nazaré da Mata	1	0	0	0	1
260960 Olinda	2	0	0	0	2
261160 Recife	7	1	0	0	8
261250 Santa Cruz do Capibaribe	1	0	0	0	1
261410 Sertânia	1	0	0	0	1
270230 Coruripe	1	0	0	0	1
270235 Craíbas	1	0	0	0	1
270240 Delmiro Gouveia	1	1	0	0	2
270430 Maceió	3	0	0	0	3
280030 Aracaju	1	0	0	0	1
280150 Carmópolis	1	0	0	0	1
290520 Caetité	1	0	0	0	1
290750 Catu	1	0	0	0	1
290810 Cocos	1	0	0	0	1
291480 Itabuna	1	0	0	0	1
291535 Itaguaçu da Bahia	1	0	0	0	1
291840 Juazeiro	0	1	0	0	1
292400 Paulo Afonso	2	0	0	0	2
292740 Salvador	5	0	0	0	5
292950 São Sebastião do Passé	1	0	0	0	1
310170 Almenara	0	1	0	0	1
310350 Araguari	0	1	0	0	1
310540 Barão de Cocais	0	2	0	0	2
310570 Barra Longa	0	1	0	0	1
310620 Belo Horizonte	0	20	0	0	20
310670 Betim	0	3	0	0	3
311140 Campo Florido	0	1	0	0	1
311570 Central de Minas	0	1	0	0	1
311860 Contagem	0	4	0	0	4
312230 Divinópolis	0	1	0	0	1
312420 Espera Feliz	0	1	0	0	1
312480 Estrela do Sul	0	1	0	0	1
312610 Formiga	0	1	0	0	1
312710 Frutal	0	3	0	0	3
312770 Governador Valadares	0	4	0	0	4
312980 Ibirité	0	1	0	0	1
313130 Ipatinga	0	2	0	0	2
313170 Itabira	0	1	0	0	1
313180 Itabirinha	0	1	0	0	1
313240 Itajubá	0	1	0	0	1
313380 Itaúna	0	1	0	0	1

313670 Juiz de Fora	0	5	0	0	5
313720 Lagoa da Prata	0	1	0	0	1
313740 Lagoa Dourada	0	1	0	0	1
313760 Lagoa Santa	0	1	0	0	1
314310 Monte Carmelo	0	1	0	0	1
314560 Oliveira	0	1	0	0	1
314590 Ouro Branco	0	1	0	0	1
314650 Pains	0	1	0	0	1
315210 Ponte Nova	0	1	0	0	1
315250 Pouso Alegre	0	1	0	0	1
315400 Raul Soares	0	1	0	0	1
315460 Ribeirão das Neves	0	1	0	0	1
315570 Rio Piracicaba	0	1	0	0	1
315780 Santa Luzia	0	1	0	0	1
316250 São João del Rei	0	1	0	0	1
316670 Serra dos Aimorés	0	1	0	0	1
316720 Sete Lagoas	0	1	0	0	1
316860 Teófilo Otoni	0	1	0	0	1
316930 Três Corações	0	1	0	0	1
316935 Três Marias	0	1	0	0	1
317010 Uberaba	0	2	0	0	2
317020 Uberlândia	0	5	0	0	5
320130 Cariacica	0	3	0	0	3
320190 Domingos Martins	0	1	0	0	1
320210 Ecoporanga	0	1	0	0	1
320240 Guarapari	0	4	0	0	4
320305 Jaguaré	0	1	0	0	1
320320 Linhares	0	2	0	0	2
320455 Santa Maria de Jetibá	0	1	0	0	1
320460 Santa Teresa	0	1	0	0	1
320500 Serra	0	1	0	0	1
320506 Venda Nova do Imigrante	0	1	0	0	1
320520 Vila Velha	0	4	0	0	4
320530 Vitória	0	2	0	0	2
330030 Barra do Piraí	0	3	0	0	3
330045 Belford Roxo	0	1	0	0	1
330170 Duque de Caxias	0	2	0	0	2
330190 Itaboraí	0	1	0	0	1
330200 Itaguaí	0	1	0	0	1
330240 Macaé	0	1	0	0	1
330250 Magé	0	1	0	0	1
330285 Mesquita	0	1	0	0	1
330330 Niterói	0	3	0	0	3
330340 Nova Friburgo	0	1	0	0	1
330350 Nova Iguaçu	0	3	0	0	3
330414 Queimados	0	2	0	0	2
330455 Rio de Janeiro	0	43	0	0	43
330490 São Gonçalo	0	1	0	0	1
350070 Agudos	0	1	0	0	1
350170 Américo Brasiliense	0	1	0	0	1

350280 Araçatuba	0	2	0	0	2
350320 Araraquara	0	1	0	0	1
350480 Bálsamo	0	1	0	0	1
350550 Barretos	0	1	0	0	1
350590 Batatais	0	2	0	0	2
350600 Bauru	0	1	0	0	1
350700 Boituva	0	1	0	0	1
350760 Bragança Paulista	0	2	0	0	2
350850 Caçapava	0	1	0	0	1
350920 Cajamar	0	1	0	0	1
350950 Campinas	0	3	0	1	4
351000 Cândido Mota	0	2	0	0	2
351060 Carapicuíba	0	1	0	0	1
351170 Charqueada	0	1	0	0	1
351190 Clementina	0	1	0	0	1
351620 Franca	0	1	0	0	1
351780 Guaraçaí	0	1	0	0	1
351880 Guarulhos	0	8	0	0	8
352220 Itapecerica da Serra	0	1	0	0	1
352310 Itaquaquecetuba	0	2	0	0	2
352390 Itu	0	2	0	0	2
352470 Jaguariúna	0	1	0	0	1
352510 Jardinópolis	0	1	0	0	1
352530 Jaú	0	1	0	0	1
352590 Jundiaí	0	1	0	0	1
352680 Lençóis Paulista	0	2	0	0	2
352690 Limeira	0	1	0	0	1
352890 Mariápolis	0	1	0	0	1
353080 Moji Mirim	0	2	0	0	2
353390 Olímpia	0	1	0	0	1
353440 Osasco	0	1	0	0	1
353625 Parisi	0	1	0	0	1
353770 Piacatu	0	1	0	0	1
353870 Piracicaba	0	1	0	0	1
354140 Presidente Prudente	0	1	0	0	1
354170 Quatá	0	2	0	0	2
354340 Ribeirão Preto	0	5	0	0	5
354580 Santa Bárbara d'Oeste	0	1	0	0	1
354730 Santana de Parnaíba	0	2	0	0	2
354780 Santo André	0	2	0	0	2
354850 Santos	0	2	0	0	2
354910 São João da Boa Vista	0	1	0	0	1
354980 São José do Rio Preto	0	6	0	0	6
354990 São José dos Campos	0	2	0	0	2
355030 São Paulo	0	39	0	0	39
355100 São Vicente	0	1	0	0	1
355150 Serrana	0	1	0	0	1
355220 Sorocaba	0	2	0	0	2
355360 Tapiratiba	0	1	0	0	1
355400 Tatuí	0	1	0	0	1

355500 Tupã	0	1	0	0	1
355650 Várzea Paulista	0	1	0	0	1
355670 Vinhedo	0	1	0	0	1
410110 Andirá	0	0	1	0	1
410345 Cafelândia	0	0	1	0	1
410405 Campo Bonito	0	0	1	0	1
410420 Campo Largo	0	0	1	0	1
410440 Cândido de Abreu	0	0	1	0	1
410480 Cascavel	0	0	2	0	2
410690 Curitiba	0	1	7	0	8
410830 Foz do Iguaçu	0	0	5	0	5
410832 Francisco Alves	0	0	1	0	1
410840 Francisco Beltrão	0	0	1	0	1
410940 Guarapuava	0	0	1	0	1
410990 Icaraíma	0	0	1	0	1
411370 Londrina	0	0	2	0	2
411460 Marechal Cândido Rondon	0	0	1	0	1
411490 Marilândia do Sul	0	0	1	0	1
411520 Maringá	0	0	3	0	3
411585 Mercedes	0	0	1	0	1
411725 Nova Prata do Iguaçu	0	0	2	0	2
411990 Ponta Grossa	0	0	1	0	1
412090 Quedas do Iguaçu	0	0	1	0	1
412230 Rio Negro	0	0	1	0	1
412300 Salto do Lontra	0	0	1	0	1
412380 Santa Izabel do Oeste	0	0	1	0	1
412550 São José dos Pinhais	0	0	4	0	4
412570 São Miguel do Iguaçu	0	0	1	0	1
412730 Terra Rica	0	0	1	0	1
412740 Terra Roxa	0	0	1	0	1
420200 Balneário Camboriú	0	0	1	0	1
421280 Balneário Piçarras	0	0	1	0	1
420210 Barra Velha	0	0	1	0	1
420290 Brusque	0	0	1	0	1
420310 Caibi	0	0	1	0	1
420420 Chapecó	0	0	1	0	1
420435 Cordilheira Alta	0	0	1	0	1
420440 Coronel Freitas	0	0	1	0	1
420470 Cunha Porã	1	0	0	0	1
420540 Florianópolis	0	0	5	0	5
420570 Garopaba	0	0	1	0	1
420590 Gaspar	0	0	1	0	1
420640 Guaraciaba	0	0	1	0	1
420800 Itá	0	0	1	0	1
420820 Itajaí	0	0	5	0	5
420890 Jaraguá do Sul	0	0	1	0	1
420915 José Boiteux	1	0	0	0	1
421050 Maravilha	0	0	1	0	1
421220 Papanduva	0	0	2	0	2
421480 Rio do Sul	0	0	1	0	1

421630 São João Batista	0	0	1	0	1
421690 São Lourenço do Oeste	0	0	1	0	1
421720 São Miguel do Oeste	0	0	0	1	1
421740 Schroeder	0	0	1	0	1
421840 Treze de Maio	0	0	1	0	1
421900 Urussanga	2	0	0	0	2
421970 Xaxim	0	0	1	0	1
430195 Barra Funda	0	0	1	0	1
430860 Garibaldi	0	0	1	0	1
431410 Passo Fundo	0	0	2	0	2
431490 Porto Alegre	0	0	1	0	1
431710 Sant' Ana do Livramento	0	0	1	0	1
431740 Santiago	0	0	1	0	1
432230 Tuparendi	0	0	1	0	1
500270 Campo Grande	0	0	0	6	6
500370 Dourados	0	0	0	1	1
500460 Itaquiraí	0	0	0	2	2
500769 São Gabriel do Oeste	0	0	0	3	3
500790 Sidrolândia	0	0	0	1	1
510840 Várzea Grande	0	1	0	0	1
520110 Anápolis	0	0	0	1	1
520140 Aparecida de Goiânia	0	0	0	4	4
520450 Caldas Novas	0	0	0	2	2
520860 Goianésia	0	0	0	3	3
520870 Goiânia	0	0	0	18	18
520880 Goianira	0	0	0	2	2
520890 Goiás	0	0	0	1	1
521308 Minaçu	0	0	0	3	3
521460 Niquelândia	0	0	0	1	1
521850 Quirinópolis	0	0	0	1	1
521860 Rialma	0	0	0	1	1
521880 Rio Verde	0	0	0	2	2
522010 São Luís de Montes Belos	0	0	0	2	2
522045 Senador Canedo	0	0	0	1	1
522140 Trindade	0	0	0	1	1
522160 Uruaçu	0	0	0	1	1
530010 Brasília	0	1	0	23	24
Total	123	312	88	84	607

MALÁRIA - Casos confirmados Notificados no Sistema de Informação de Agravos de Notificação - Sinan Net

Casos confirmados por Munic. Residência e result.parasitológi
Autoctone Mun Res: Não
Período:2014

Munic. Residência	Falciparum	F+FG	Vivax	F+V	Total
110004 Cacoal	0	1	0	0	1
110020 Porto Velho	1	0	0	0	1
130020 Atalaia do Norte	0	1	0	0	1
130260 Manaus	0	1	0	0	1
160030 Macapá	1	0	0	0	1
210300 Caxias	1	0	0	0	1
210330 Codó	1	0	0	0	1
210570 Lago da Pedra	1	1	0	0	2
220200 Buriti dos Lopes	0	1	0	0	1
220220 Campo Maior	2	0	0	0	2
220580 Luzilândia	1	0	0	0	1
220585 Madeiro	1	0	0	0	1
220610 Matias Olímpio	1	0	0	0	1
220650 Monsenhor Hipólito	5	0	0	0	5
220840 Piripiri	1	0	0	0	1
220880 Regeneração	1	0	0	0	1
221100 Teresina	9	0	0	0	9
221110 União	1	0	0	0	1
230440 Fortaleza	7	0	0	0	7
230620 Itaiçaba	1	0	0	0	1
231310 Tabuleiro do Norte	1	0	0	0	1
231410 Viçosa do Ceará	1	0	0	0	1
240810 Natal	5	0	0	0	5
240325 Parnamirim	1	0	0	0	1
241200 São Gonçalo do Amarante	1	0	0	0	1
240000 Município ignorado - RN	1	0	0	0	1
260010 Afogados da Ingazeira	1	0	0	0	1
260340 Calumbi	1	0	0	0	1
260940 Moreno	1	0	0	0	1
261000 Palmares	1	0	0	0	1
261070 Paulista	1	0	0	0	1
261140 Primavera	1	0	0	0	1
261160 Recife	5	0	0	0	5
261450 Surubim	1	0	0	0	1
270030 Arapiraca	1	1	0	0	2
270430 Maceió	2	0	0	0	2
270470 Marechal Deodoro	1	0	0	0	1
280030 Aracaju	3	0	0	0	3
280480 Nossa Senhora do Socorro	1	0	0	0	1
280540 Poço Redondo	1	0	0	0	1
290570 Camaçari	1	0	0	0	1
290630 Canavieiras	1	0	0	0	1
290650 Candeias	1	0	0	0	1

291800 Jequié	1	0	0	0	1
292230 Muritiba	1	0	0	0	1
292400 Paulo Afonso	2	0	0	0	2
292740 Salvador	8	0	0	0	8
310110 Aimorés	0	2	0	0	2
310350 Araguari	0	1	0	0	1
310620 Belo Horizonte	0	7	0	0	7
311860 Contagem	0	1	0	0	1
312737 Goiabeira	0	1	0	0	1
313170 Itabira	0	4	0	0	4
313240 Itajubá	0	1	0	0	1
313310 Itanhandu	0	1	0	0	1
313320 Itanhomi	0	2	0	0	2
313380 Itaúna	0	1	0	0	1
313670 Juiz de Fora	0	2	0	0	2
313750 Lagoa Formosa	0	1	0	0	1
313760 Lagoa Santa	0	1	0	0	1
314790 Passos	0	1	0	0	1
314800 Patos de Minas	0	2	0	0	2
315340 Presidente Olegário	0	1	0	0	1
315460 Ribeirão das Neves	0	1	0	0	1
317010 Uberaba	0	1	0	0	1
317020 Uberlândia	0	2	0	0	2
320010 Afonso Cláudio	0	1	0	0	1
320130 Cariacica	0	1	0	0	1
320220 Fundão	0	2	0	0	2
320260 Iconha	0	1	0	0	1
320320 Linhares	0	3	0	0	3
320332 Marataízes	0	1	0	0	1
320334 Marechal Floriano	0	1	0	0	1
320500 Serra	0	2	0	0	2
320520 Vila Velha	0	2	0	0	2
320530 Vitória	0	3	0	0	3
330070 Cabo Frio	0	1	0	0	1
330100 Campos dos Goytacazes	0	3	0	0	3
330170 Duque de Caxias	0	2	0	0	2
330240 Macaé	0	4	0	0	4
330285 Mesquita	0	1	0	0	1
330350 Nova Iguaçu	0	1	0	0	1
330452 Rio das Ostras	0	2	0	0	2
330455 Rio de Janeiro	0	32	0	0	32
330490 São Gonçalo	0	4	0	0	4
330510 São João de Meriti	0	1	0	0	1
350330 Araras	0	1	0	0	1
350460 Bady Bassitt	0	2	0	0	2
350570 Barueri	0	2	0	0	2
350600 Bauru	0	1	0	0	1
350850 Caçapava	0	3	0	0	3
350950 Campinas	0	4	0	0	4
351060 Carapicuíba	0	1	0	0	1

351100 Castilho	0	2	0	0	2
351240 Cordeirópolis	0	1	0	0	1
351410 Dois Córregos	0	1	0	0	1
351640 Franco da Rocha	0	1	0	0	1
351740 Guaíra	0	1	0	0	1
351830 Guararema	0	1	0	0	1
351870 Guarujá	0	1	0	0	1
351880 Guarulhos	0	3	0	0	3
352044 Ilha Solteira	0	0	0	1	1
352230 Itapetininga	0	1	0	0	1
352390 Itu	0	1	0	0	1
352430 Jaboticabal	0	1	0	0	1
352500 Jandira	0	2	0	0	2
352640 Laranjal Paulista	0	1	0	0	1
352690 Limeira	0	3	0	0	3
352820 Macedônia	0	1	0	0	1
352940 Mauá	0	1	0	0	1
353030 Mirassol	0	1	0	0	1
353180 Monte Mor	0	1	0	0	1
353270 Nipoã	0	1	0	0	1
353440 Osasco	0	1	0	0	1
353650 Paulínia	0	1	0	0	1
353730 Penápolis	0	1	0	0	1
353800 Pindamonhangaba	0	1	0	0	1
353870 Piracicaba	0	2	0	0	2
353920 Pirapozinho	0	1	0	0	1
353930 Pirassununga	0	1	0	0	1
354000 Pompéia	0	1	0	0	1
354120 Presidente Bernardes	0	1	0	0	1
354330 Ribeirão Pires	0	1	0	0	1
354340 Ribeirão Preto	0	5	0	0	5
354520 Salto	0	1	0	0	1
354660 Santa Fé do Sul	0	1	0	0	1
354780 Santo André	0	1	0	0	1
354850 Santos	0	1	0	0	1
354870 São Bernardo do Campo	0	1	0	0	1
354880 São Caetano do Sul	0	1	0	0	1
354910 São João da Boa Vista	0	1	0	0	1
354980 São José do Rio Preto	0	1	0	0	1
354990 São José dos Campos	0	2	0	0	2
354995 São Lourenço da Serra	0	1	0	0	1
355030 São Paulo	0	35	0	0	35
355070 São Sebastião	0	1	0	0	1
355100 São Vicente	0	2	0	0	2
355240 Sumaré	0	1	0	0	1
355280 Taboão da Serra	0	1	0	0	1
355540 Ubatuba	0	1	0	0	1
355580 Urânia	0	1	0	0	1
355670 Vinhedo	0	1	0	0	1
410040 Almirante Tamandaré	0	0	2	0	2

410420 Campo Largo	0	0	2	0	2
410480 Cascavel	0	0	1	0	1
410690 Curitiba	0	0	8	0	8
410790 Floresta	0	0	1	0	1
410830 Foz do Iguaçu	0	0	3	0	3
410840 Francisco Beltrão	0	0	1	0	1
411070 Irati	0	0	1	0	1
411270 Jataizinho	0	0	1	0	1
411520 Maringá	0	0	3	0	3
411580 Medianeira	0	0	1	0	1
411915 Pinhais	0	0	1	0	1
411990 Ponta Grossa	0	0	1	0	1
412550 São José dos Pinhais	0	1	0	0	1
412570 São Miguel do Iguaçu	0	0	1	0	1
412770 Toledo	0	0	1	0	1
420200 Balneário Camboriú	0	0	1	0	1
420240 Blumenau	0	0	1	0	1
420380 Canoinhas	0	0	1	0	1
420460 Criciúma	0	0	1	0	1
420540 Florianópolis	0	0	3	0	3
420580 Garuva	0	0	1	0	1
420610 Grão Pará	0	0	1	0	1
420650 Guaramirim	0	0	2	0	2
420820 Itajaí	0	0	1	0	1
420880 Jaguaruna	0	0	1	0	1
421190 Palhoça	0	0	2	0	2
421225 Passo de Torres	0	0	1	0	1
421350 Porto Belo	0	0	1	0	1
421575 São Bernardino	0	0	1	0	1
421660 São José	0	0	3	0	3
421720 São Miguel do Oeste	0	0	3	0	3
421870 Tubarão	0	1	1	0	2
421970 Xaxim	0	0	1	0	1
430460 Canoas	0	0	1	0	1
430510 Caxias do Sul	0	0	1	0	1
430610 Cruz Alta	0	0	1	0	1
430790 Farroupilha	0	0	1	0	1
431180 Marau	0	0	2	0	2
431440 Pelotas	0	0	1	0	1
431490 Porto Alegre	0	0	2	0	2
431690 Santa Maria	0	0	1	0	1
431730 Santa Vitória do Palmar	0	0	1	0	1
431775 Santo Antônio do Planalto	0	0	1	0	1
431971 São Valentim do Sul	0	0	1	0	1
432140 Tenente Portela	0	0	1	0	1
432250 Vacaria	0	0	1	0	1
432260 Venâncio Aires	0	0	1	0	1
500100 Aparecida do Taboado	0	0	0	1	1
500270 Campo Grande	0	0	0	1	1
500325 Costa Rica	0	0	0	1	1

500830 Três Lagoas	0	0	0	1	1
510340 Cuiabá	0	0	0	1	1
510790 Sinop	0	1	0	0	1
520110 Anápolis	0	0	0	4	4
520130 Anicuns	0	0	0	1	1
520140 Aparecida de Goiânia	0	0	0	7	7
520549 Cidade Ocidental	0	0	0	1	1
520870 Goiânia	0	0	0	12	12
520880 Goianira	0	0	0	1	1
521190 Jataí	0	0	0	1	1
521308 Minaçu	0	0	0	2	2
521570 Palmeiras de Goiás	0	0	0	1	1
521850 Quirinópolis	0	0	0	1	1
522010 São Luís de Montes Belos	0	0	0	2	2
522140 Trindade	0	0	0	2	2
522185 Valparaíso de Goiás	0	0	0	1	1
521820 Porto Nacional (transf. p/TO)	0	0	0	1	1
530010 Brasília	0	0	0	12	12
Total	81	219	70	55	425

ID_PAIS	COPAISINF	NM_PAIS
2	2	GABAO
3	3	GEORGIA
4	4	IEMEM
5	5	LIBIA
6	6	MARROCOS
7	7	PERU
8	8	SEYCHELLES
9	9	SUICA
10	10	TURCOMENISTAO
11	11	SOMALIA
12	12	BARBADOS
13	13	CAMBOJA
14	14	CAZAQUISTAO
15	15	GUINE
16	16	IRAQUE
17	17	IRLANDA
18	18	ISLANDIA
19	19	ITALIA
20	20	LIECHTENSTEIN
21	21	MEXICO
22	22	MOCAMBIQUE
23	23	SUECIA
24	24	NORUEGA
25	25	POLONIA
26	26	REPUBLICA TCHECA
27	27	SANTA LUCIA
28	28	SUDAO
29	29	TAIWAN (ILHA FORMOSA)
30	30	UZBEQUISTAO
31	31	ANGOLA
32	32	ARGENTINA
33	33	BRUNEI
34	34	COREIA DO SUL
35	35	LUXEMBURGO
36	36	MALDIVAS
37	37	PAISES BAIXOS
38	38	REINO UNIDO
39	39	UCRANIA
40	40	VIETNA
41	41	ALEMANHA
42	42	ANTIGUA E BARBUDA
43	43	BELGICA
44	44	ESPANHA
45	45	ESTADOS UNIDOS
46	46	FRANCA
47	47	KUWEIT
48	48	ROMENIA
49	49	RUSSIA
50	50	SIRIA

**51**	**51**	ZAIRE
**52**	**52**	BAHAMAS
**54**	**54**	ISRAEL
**55**	**55**	JAPAO
**56**	**56**	MONACO
**57**	**57**	PORTO RICO
**58**	**58**	GUADALUPE
**59**	**59**	ANTILHAS HOLANDESAS
**60**	**60**	DJIBUTI
**61**	**61**	BUTAO
**62**	**62**	MYANMAR
**63**	**63**	NAURU
**64**	**64**	TUVALU
**65**	**65**	ALBANIA
**66**	**66**	ARGELIA
**67**	**67**	BELIZE
**68**	**68**	BOLIVIA
**69**	**69**	BOTSUANA
**70**	**70**	ERITREIA
**71**	**71**	EQUADOR
**72**	**72**	HONDURAS
**73**	**73**	LAOS
**74**	**74**	LESOTO
**75**	**75**	LIBANO
**76**	**76**	LIBERIA
**77**	**77**	MALI
**78**	**78**	MONGOLIA
**79**	**79**	NAMIBIA
**80**	**80**	NIUE
**1**	**1**	BRASIL
**81**	**81**	QUIRGUISTAO
**82**	**82**	QUIRIBATI
**83**	**83**	SUAZILANDIA
**84**	**84**	SURINAME
**85**	**85**	TADJIQUISTAO
**86**	**86**	TANZANIA
**88**	**88**	TOGO
**89**	**89**	TONGA
**90**	**90**	TRINIDAD E TOBAGO
**91**	**91**	TUNISIA
**92**	**92**	AUSTRALIA
**93**	**93**	BAHREIN
**94**	**94**	BIELORUSSIA
**95**	**95**	CABO VERDE
**96**	**96**	CANADA
**97**	**97**	CATAR
**98**	**98**	CHADE
**99**	**99**	CHIPRE
**100**	**100**	COSTA RICA
**101**	**101**	DOMINICA

**102**	**102**	EGITO
**103**	**103**	EL SALVADOR
**104**	**104**	ESLOVAQUIA
**105**	**105**	ESTONIA
**106**	**106**	FIJI
**107**	**107**	FILIPINAS
**108**	**108**	GAMBIA
**109**	**109**	GANA
**110**	**110**	GUATEMALA
**111**	**111**	GUIANA FRANCESA
**112**	**112**	GUINE-BISSAU
**113**	**113**	GUINE EQUATORIAL
**114**	**114**	HAITI
**115**	**115**	ILHAS MARSHAL
**116**	**116**	IRA
**118**	**118**	LITUANIA
**119**	**119**	MACEDONIA
**120**	**120**	MALAVI
**121**	**121**	MAURITANIA
**122**	**122**	MOLDOVA
**123**	**123**	NEPAL
**124**	**124**	PANAMA
**126**	**126**	PARAGUAI
**127**	**127**	QUENIA
**128**	**128**	REPUBLICA CENTRO-AFRICANA
**129**	**129**	REPUBLICA DOMINICANA
**130**	**130**	ILHAS SALOMAO
**131**	**131**	SAMOA OCIDENTAL
**132**	**132**	SAN MARINO
**133**	**133**	SENEGAL
**134**	**134**	SERRA LEOA
**135**	**135**	SRI LANKA
**136**	**136**	TURQUIA
**137**	**137**	URUGUAI
**138**	**138**	VENEZUELA
**139**	**139**	ZIMBABUE
**140**	**140**	AFRICA DO SUL
**141**	**141**	ARABIA SAUDITA
**142**	**142**	ARMENIA
**143**	**143**	AUSTRIA
**144**	**144**	AZERBAIJAO
**145**	**145**	BANGLADESH
**146**	**146**	BENIN
**147**	**147**	BOSNIA-HERZEGOVINA
**148**	**148**	BULGARIA
**149**	**149**	BURKINA FASO
**150**	**150**	BURUNDI
**151**	**151**	COLOMBIA
**152**	**152**	CAMAROES
**153**	**153**	CONGO

**154**	**154**	COSTA DO MARFIM
**155**	**155**	CROACIA
**156**	**156**	CUBA
**157**	**157**	EMIRADOS ARABES UNIDOS
**158**	**158**	ESLOVENIA
**159**	**159**	ETIOPIA
**160**	**160**	FINLANDIA
**161**	**161**	GRANADA
**162**	**162**	GRECIA
**163**	**163**	HUNGRIA
**164**	**164**	INDIA
**165**	**165**	INDONESIA
**166**	**166**	JAMAICA
**167**	**167**	JORDANIA
**168**	**168**	LETONIA
**170**	**170**	MADAGASCAR
**171**	**171**	MALASIA
**172**	**172**	MALTA
**173**	**173**	MAURICIO
**174**	**174**	MICRONESIA
**175**	**175**	NICARAGUA
**176**	**176**	NIGER
**177**	**177**	NIGERIA
**178**	**178**	NOVA ZELANDIA
**179**	**179**	OMA
**180**	**180**	PALAU
**181**	**181**	PAPUA NOVA GUINE
**182**	**182**	PORTUGAL
**183**	**183**	SAO CRISTOVAO E NEVES
**184**	**184**	SAO VICENTE E GRANABINAS
**185**	**185**	SAO TOME E PRINCIPE
**186**	**186**	TAILANDIA
**187**	**187**	TAITI
**188**	**188**	UGANDA
**189**	**189**	VANUATU
**190**	**190**	ZAMBIA
**191**	**191**	AFEGANISTAO
**192**	**192**	CHILE
**193**	**193**	REPUBLICA POPULAR DA CHINA
**194**	**194**	SINGAPURA
**195**	**195**	COREIA DO NORTE
**196**	**196**	DINAMARCA
**198**	**198**	TIMOR LESTE
**199**	**199**	GUIANA
**200**	**200**	ILHAS FALKLAND
**201**	**201**	ANDORRA
**202**	**202**	ILHAS COMORES
**203**	**203**	ILHAS COOK
**204**	**204**	SERVIA E MONTENEGRO
**205**	**205**	RUANDA

125 **125** PAQUISTAO

MALÁRIA - Casos confirmados Notificados no Sistema de Informação de Agravos de Notificação - Sinan Net

Casos confirmados por País F. infecção e result.parasitológi
Autoctone Mun Res: Não
Período:2007-2014

País F. infecção	Falciparum	F+FG	Vivax	F+V	Total
110 110	0	1	1	0	2
86 86	0	0	1	0	1
45 45	1	1	0	1	3
38 38	0	2	0	0	2
1 1	630	1882	713	708	3933
68 68	1	3	2	3	9
76 76	0	1	0	0	1
84 84	23	15	1	15	54
95 95	1	0	0	1	2
103 103	0	3	0	0	3
109 109	1	14	1	2	18
113 113	6	61	1	3	71
2 2	0	1	0	0	1
7 7	0	14	1	1	16
11 11	0	0	0	23	23
13 13	0	1	0	0	1
15 15	1	20	2	2	25
22 22	4	73	7	2	86
28 28	1	2	0	0	3
31 31	148	389	31	17	585
32 32	0	0	12	0	12
46 46	2	1	0	2	5
66 66	1	1	0	0	2
77 77	0	1	0	0	1
88 88	0	2	1	0	3
92 92	0	1	0	0	1
97 97	0	1	0	0	1
111 111	136	47	6	48	237
112 112	3	16	0	4	23
114 114	3	20	9	9	41
120 120	0	1	0	0	1
124 124	0	1	0	0	1
126 126	0	0	30	0	30
127 127	0	0	1	2	3
128 128	0	1	0	0	1
133 133	0	3	1	1	5
134 134	0	3	0	0	3
138 138	18	20	4	11	53
139 139	0	1	0	0	1
140 140	31	57	22	10	120
141 141	3	0	0	0	3
145 145	0	0	1	0	1
151 151	0	2	0	0	2

152	152	0	5	0	1	6
153	153	1	17	1	2	21
154	154	3	6	0	0	9
156	156	0	1	0	0	1
157	157	0	1	0	0	1
159	159	0	2	0	0	2
164	164	0	8	0	0	8
165	165	1	4	0	0	5
170	170	0	1	0	0	1
177	177	4	71	1	0	76
185	185	0	1	0	0	1
186	186	0	2	0	0	2
188	188	1	2	0	0	3
190	190	0	2	0	0	2
191	191	0	1	0	0	1
192	192	1	0	0	0	1
199	199	36	30	9	31	106
125	125	0	1	0	0	1
Total		1061	2817	859	899	5636
